# Biosynthesis of trialkyl-substituted aromatic polyketide NFAT-133 involves unusual P450 monooxygenase-mediating aromatization and a putative metallo-beta-lactamase fold hydrolase

**DOI:** 10.1016/j.synbio.2023.05.003

**Published:** 2023-06-03

**Authors:** Ming Yang, Wanlu Li, Lin Zhou, Xiao Lin, Wenyu Zhang, Yaoyao Shen, Hai Deng, Hou-wen Lin, Yongjun Zhou

**Affiliations:** a Research Center for Marine Drugs, Department of Pharmacy, Ren Ji Hospital, School of Medicine, Shanghai Jiao Tong University, Shanghai, 200127, China; b Institute of Marine Drugs, Guangxi Key Laboratory of Marine Drugs, Guangxi University of Chinese Medicine, Nanning, 530200, PR China; c Department of Chemistry, University of Aberdeen, Aberdeen, AB24 3UE, UK

**Keywords:** Thioesterase, Metallo hydrolase, Metallo-beta-lactamase, Aromatic polyketide, Polyketide synthase, P450 monooxygenase

## Abstract

The bacterial trialkyl-substituted aromatic polyketides are structurally featured with the unusual aromatic core in the middle of polyketide chain such as TM-123 (**1**), veramycin A (**2**), NFAT-133 (**3**) and benwamycin I (**4**), which were discovered from *Streptomyces* species and demonstrated with antidiabetic and immunosuppressant activities. Though the biosynthetic pathway of **1**−**3** was reported as a type I polyketide synthase (PKS), the PKS assembly line was interpreted inconsistently, and it remains a mystery how the compound **3** was generated. Herein, the PKS assembly logic of **1**−**4** was revised by site-mutagenetic analysis of the PKS dehydratase domains. Based on gene deletion and complementation, the putative P450 monooxygenase *nft*E_1_ and metallo-beta-lactamase (MBL) fold hydrolase *nft*F_1_ were verified as essential genes for the biosynthesis of **1**−**4**. The absence of *nft*E_1_ led to abolishment of **1**−**4** and accumulation of new products (**5**−**8**). Structural elucidation reveals **5**−**8** as the non-aromatic analogs of **1**, suggesting the NftE_1_-catalyzed aromatic core formation. Deletion of *nft*F_1_ resulted in disappearance of **3** and **4** with the compounds **1** and **2** unaffected. As a rare MBL-fold hydrolase from type I PKSs, NftF_1_ potentially generates the compound **3** through two strategies: catalyze premature chain-offloading as a *trans*-acting thioesterase or hydrolyze the lactone-bond of compound **1** as an esterase.

## 1 Introduction

**Fig. 1:**
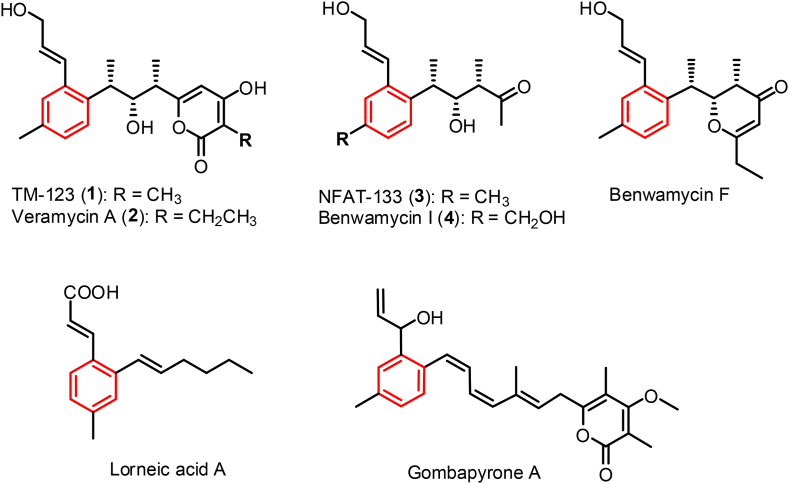
Reported trialkyl-substituted aromatic polyketides derived from type I PKSs in bacterium *Streptomyces*.

Bacterial aromatic polyketides, mostly isolated from *Actinomyces* species, represent a rich molecular source for the discovery of novel therapeutic agents [1]. The majority of them are biosynthesized by type II PKSs, which contain the monofunctional proteins of ketoacyl-synthase (KS), chain length factor, and acyl carrier protein (ACP) to carry out repetitive decarboxylative Claisen-condensation of a malonyl-CoA extending unit with an acyl starter unit to yield a reactive poly-*ß*-ketone backbone followed by aromatic cyclizations [2]. Comparatively, few aromatic polyketides are derived from type I PKSs, which feature a polyketides assembly line of the multifunctional PKS proteins consisted of the core catalytic domains of KS, acyltransferase (AT) and ACP, the accessory domains of ketoreductase (KR), dehydratase (DH) and enoylreductase (ER), and an offloading C-terminal thioesterase (TE) domain [3]. The aromatic polyketides derived from type I PKSs normally contain the terminal benzyl ring derived from the starting building block of PKS assembly [4]. Attractively, there are a group of trialkyl-substituted aromatic polyketides derived from type I PKSs in *Streptomyces*, structurally featuring with a benzene core in the middle of polyketide chain such as TM-123/veramycin B (**1**), veramycin A (**2**), NFAT-133 (**3**), benwamycin I (**4**), lorneic acids, and gombapyrones [[5], [6], [7], [8], [9], [10]] (Fig. 1). Recently, the aromatic cores of lorneic acids and gombapyrones were verified to be formed by unusual cytochrome P450 monooxygenases, which form a benzene ring in the linear polyene chain offloaded from type I PKSs [11].

**Fig. 2:**
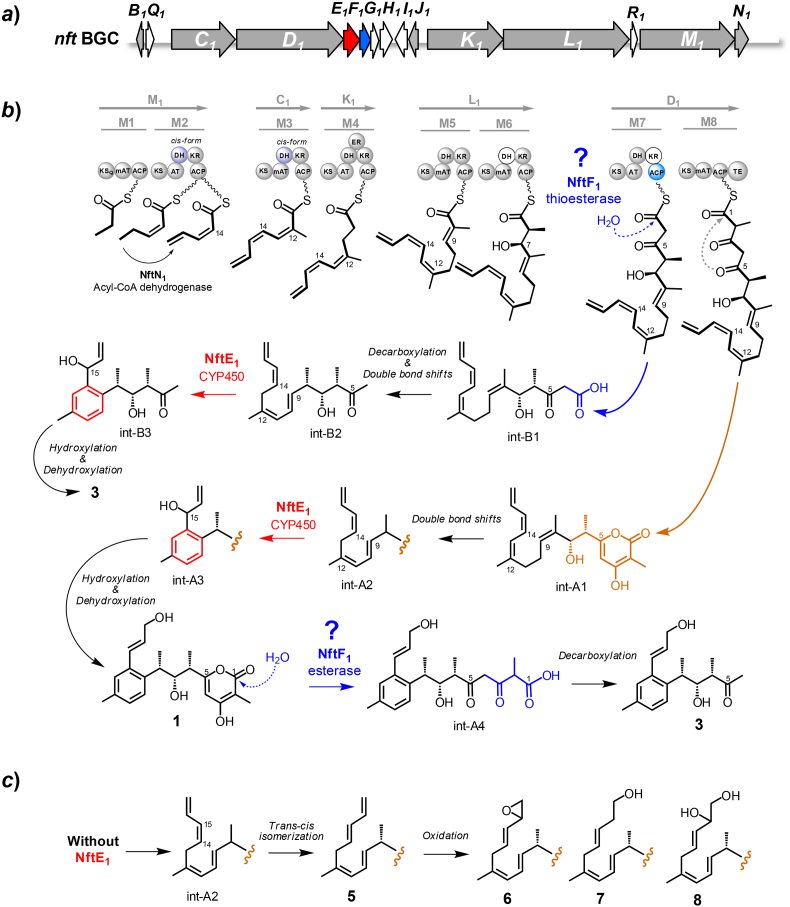
The gene organization of *nft* BGC from *S. conglobatus* (***a***), and the proposed biosynthetic logics of compounds **1** and **3** (***b***) and the mutant compounds **5**−**8** (***c***). The question marks are used to indicate the two possible working-mechanisms of NftF_1_ in producing compound **3**.

The biosynthetic pathway of **1**−**3** was investigated through genetic mutation, metabolite characterization and isotope feeding experiments in two *Streptomyces* species which contain cognate biosynthetic gene cluster of **1**−**3** (*nft* BGC) [8,12]. However, the PKS assembly line was interpreted inconsistently, and it remains a mystery how the compound **3** is generated in the biosynthetic pathway. The structure of compound **3** appears as a truncated portion of the compound **1** being lost three carbons in the terminal *α*-pyrone ring of compound **1** (Fig. 1). This raises a hypothesis that compound **3** could be derived from a chain prematurely offloaded from ACP7 by a *trans*-acting TE, for instance a MBL-fold hydrolase encoded by *nft*F_1_ (Fig. 2). Conventionally, type I PKSs offload chain by an *ɑ*/*ß* hydrolase-type TE domain integrated at the C-terminus of PKS assembly line via thioester hydrolysis or O–C macrocyclization [13]. So far, there are not documented MBL-fold TEs from type I PKSs, and only two cases of MBL-fold hydrolases were reported in the type II PKSs of actinorhodin and murayaquinone, in which ActIV and MrqD were characterized as MBL-fold TEs for chain release based on genetic mutation and biochemical analysis with an artificial substrate [14,15]. Interestingly, these cognate genes are discovered frequently in type II PKS BGCs and annotated as ring cyclase/aromatase such as RslC2, AlnR and Ssfy2 from the biosynthetic pathways of rishirilides, alnumycin, and tetracycline SF2575 [[16], [17], [18]].

In this study, the PKS assembly line of **1**−**3** was revised in *Streptomyces conglobatus*, based on site-directed mutagenesis of the PKS genes. Structure elucidation of the mutant metabolites suggested an unusual P450 monooxygenase-mediated aromatization occurred in the polyketide biosynthesis of **1**−**3**. Moreover, the production of **3** was identified to be controlled by a MBL-fold hydrolase homolog, which could perform premature chain-offloading as a new-type TE in type I PKSs or hydrolyze the pyrone ring of compound **1** as an esterase.

## 2 Materials and methods

### 2.1 Chemical reagents and kits

The primers (Table S1) and Plasmid DNA Extraction Kit were ordered from Shanghai Generay Biotech (China). DNA sequencing was provided by Shanghai Sangon Biotech (China). DNA fragments were assembled by using 2 × Ezmax-Muli CloneMix Plus (Shanghai Tolo Biotech, China). PCR amplifications were carried out by using KOD OnePCR Master Mix (TOYOBO) for gene cloning and by using 2 × Flash PCR MasterMix (CoWin Biotech, China) for colony screening. Restriction endonucleases and T4 DNA ligase were purchased from New England Biolabs. Genomic DNA was prepared by using 10% Chelex 100 resin (Bio-Rad) solution. Other chemical reagents used were purchased from Sigma-Aldrich.

### 2.2 Strains and plasmids

The RJ2 strain was used as starting strain in the study, which was generated by abolishing the conglobatin production from *S. conglobatus* ATCC 31005 [19]. *E. coli* DH10B strain was used for plasmid construction. *E.coli* ET12567 strain containing pUZ8002 plasmid was used for conjugation with *Streptomyces*. *E. coli* BL21 (DE3) plysS strain was used for protein expression. Genetic mutations in RJ2 strain were created by using the *E. coli*−*Streptomyces* shuttle plasmid pRJ2 [20]. Gene complementation was performed by using the plasmids derived from pRJ5 that contains a phage *phiC*31 integration element [21]. Plasmid pET-29a was used for protein expression in *E. coli*. All the plasmids constructed in this study was listed in Table S2.

### 2.3 Media and culture conditions

TSBY medium (3% tryptone soy broth, 0.5% yeast extract, 10% sucrose, 0.1% antifoam) was used to culture the mycelium of *Streptomyces*. SFM agar medium (2% soyean flour, 2% D-mannitol, 2% agar, pH7.4) was used for conjugation and mutant screening of *Streptomyces*. The SGC medium (3% soybean flour, 5% glucose, 0.5% CaCO_3_, and 0.1% (v/v) antifoam) was used for liquid fermentation. Large-scale fermentation of *Streptomyces* was carried out in a 500 mL conical flask fitted with a metal spring, with 150 mL of SGC medium inoculated with 1% (v/v) of 3-day TSBY culture, and then incubated at 30 °C, 220 rpm for 5 days. For small-scale fermentation, 250 mL conical flask containing 50 ml medium was used. *E. coli* strains were grown at 37 °C in the Luria-Bertani (LB) broth (1% tryptone, 0.5% yeast extract, 0.5% NaCl) or the solid medium (LB plus 1.5% agar) supplemented corresponding antibiotics.

### 2.4 Isolation of compounds

For isolation of compounds **1**–**4**, a 12-L SGC culture broth of RJ2 strain was extracted five times with an equal volume of ethyl acetate. The combined organic phase was concentrated under vacuum evaporation to yield 12 g syrup, which was subjected to a vacuum liquid chromatography with silica gel (200–300 mesh) in a stepwise elution of CH_2_Cl_2_–CH_3_OH (from 100/1 to 0/1, v/v). Guided by HPLC–MS analysis, the fractions containing target compounds were combined for further separation on an ODS chromatography column (Santai Technologies, Inc., Spherical C18, 31.2 × 257.4 mm, 15 μm, 100 Å) by using MPLC (medium pressure preparative liquid chromatography) under the elution conditions of 20 mL/min and 20–100% CH_3_CN/H_2_O (0.1% formic acid) for 6 h. The target fractions were separated by using preparative HPLC fitted with a semipreparative column (Waters Xbridge C18, 10 × 250 mm, 5 μm) with an elution condition of 60% CH_3_OH/H_2_O (0.1% formic acid) and 3 mL/min to yield 20.4 mg of **1**, 2.5 mg of **2**, 29.6 mg of **3**, and 5.5 mg of **4**.

For isolation of compounds **5**–**8**, a 24-L SGC culture broth of Δ*nft*E_1_ strain was extracted with equal volume ethyl acetate for 5 times. The combined extracts were concentrated via vacuum evaporation to yield 18 g syrup, which was separated through a Sephadex LH-20 column chromatography by using the elution of CH_2_Cl_2_/CH_3_OH (1/1, v/v) to give 17 fractions. With the guidance of HPLC–MS analysis, the target products were detected in the fractions 7–10, which were combined and further separated by the MPLC same as above. The mobile phase of CH_3_CN and H_2_O (0.1% formic acid, v/v) was eluted at 25 mL/min with a gradient condition of 10–80% CH_3_CN for 3 h and 80–100% CH_3_CN for 30 min. According to HPLC–MS analysis, all the 65 fractions were pooled into the 16 fractions. Compound **5** was directly obtained as clearing white crystal (32 mg). Compound **6** (5.9 mg), **7** (19.4 mg), and **8** (37.5 mg) were separated from these fractions by using the same preparative HPLC as above under the condition of 3.0 mL/min and 60% CH_3_OH/H_2_O (0.1% FA, v/v).

### 2.5 Mutant construction and gene complementation

To generate each of the deletion mutations: *nft*E_1_−I_1_, *nft*C_1_-DH3 (H972Y), *nft*K_1_*-*DH4 (H969Y), *nft*D_1_-DH7 (H964Y), Δ*nft*E_1_, Δ*nft*F_1_, Δ*nft*M_1_, and Δ*nft*G_1_, the two DNA fragments used for homologous recombination were prepared through PCR amplification with the genomic DNA of RJ2 as template and the related primers (see primers and descriptions in Table S1). By using Gibson assembly method, the two PCR fragments were assembled with the *E. coli*–*Streptomyces* shuttle plasmid pRJ2 linearized by *Xba*I and *Eco*RI. The resulting plasmids (Table S2) were individually introduced into the RJ2 strain by conjugation. The target mutant strains were screened by using colony PCR with the related primers (Table S1) from the hygromycin-sensitive colonies prepared after two rounds of propagation on the SFM plates without antibiotics added. The mutant strains were confirmed by sequencing the PCR products.

For mutant complementation, the target genes were amplified by PCR using the genomic DNA of RJ2 as template and the corresponding primers (Table S1). The synthesized fragment of constitutive strong promoter KasO*p [22] and a gene fragment were assembled with an integrative vector pRJ5 (*Nsi*I and *Eco*RV) by Gibson assembly. The resulting plasmids (Table S2) were transformed into the corresponding mutant strains by conjugation. The exconjugants were selected based on hygromycin-resistance and sequencing of the PCR product.

### 2.6 Analytical methods

For routine HPLC–MS analysis, 7 mL of 5-d fermentation broth was extracted with an equal volume of ethyl acetate. Then 5 mL of the extract was collected and dried over by nitrogen blowing. The samples were redissolved into 800 μL of CH_3_OH and centrifuged at 12000 rpm for 6 min before injecting 20 μL supernatant for analysis. The HPLC–MS analysis was performed on a Waters HPLC coupled with a Waters Acquity QDa detector and a Waters Xbridge C18 column (250 mm × 4.6 mm, 5 μm). Samples were eluted with the mobile phases of CH_3_CN and H_2_O (0.1% formic acid, v/v) at a flow rate of 0.7 mL/min with a gradient elution of 30–100% CH_3_CN over 30 min. The mass spectrometer was run in positive ionization mode, scanning from *m*/*z* 200 to 1250 with capillary (0.80 kV), cone (15.00 V), and source temperature (120 °C). The HRMS spectra were recorded in a Waters XeVO G2-XS Q TOF mass spectrometer conducted in positive ionization mode, scanning from *m*/*z* 100 to 1200. NMR spectra were recorded on Bruker 600 MHz or Bruker NEO 700 MHz instruments. The compounds **2** and **3** were analyzed in CD_3_CN-*d*_*3*_ and CDCl_3_, respectively, and the other compounds were detected in DMSO-*d*_6_. Chemical shifts (*δ*) were obtained in reference to tetramethylsilane (TMS) at 0.00 ppm.

### 2.7 X-ray crystallographic analysis

Crystals data was obtained on Bruker D8 VENTURE diffractometer (Bruker Corp., Karlsruhe, Germany). The crystal structures were solved by direct methods using the SHELXT-2015 software package and refined by the full matrix least-squares method. In the structure refinements, nonhydrogen atoms were refined anisotropically, and the hydrogen atom positions were geometrically idealized and allowed to ride on their parent atoms. The crystallographic data of **5** was deposited in Cambridge Crystallographic Data Center (CCDC number: 2246505).

### 2.8 NftE_1_ and NftF_1_ protein expression and enzymatic assay

For constructing protein expression plasmids, the DNA fragment of *nft*E_1_ or *nft*F_1_ was amplified from the genomic DNA of RJ2 with the corresponding primers (Table S1). The C-terminal 6 × His tag was introduced by the antisense primer. Each of the DNA fragments was introduced at *Nde*I and *Xho*I sites of pET29a through Gibson assembly. The resulting plasmids (Table S2) were transferred into *E. coli* BL21 (DE3) plysS for protein expression.

For protein expression fermentation, 1-mL overnight culture of the target transformant was inoculated into 500-mL LB medium containing 50 μg/mL kanamycin and incubated at 37 °C and 220 rpm. Protein expression was induced by adding 0.2 mM Isopropyl-β-d-thiogalactopyranoside once the OD_600_ reached 0.4–0.6. After further cultivation for 15 h at 22 °C, the cells were harvested by centrifugation (11,325×*g*, 5 min) and resuspended in buffer A (50 mM Tris-HCl, 300 mM NaCl, pH 7.2) for sonication. The cell lysate was centrifuged at 34,925×*g* for 25 min, and the supernatant was filtrated through a 0.22-μm filter before loaded onto a His-Bind affinity column (1-mL bed volume). The target proteins were separated by stepwise increases of the concentration of imidazole (10–500 mM). Based on SDS-PAGE (12.5%) analysis, the target fractions were concentrated and desalted by using Amicon Ultra-4 concentrators (Millipore, 30-kDa cutoff) and PD10 column (GE Healthcare). Protein concentrations were measured by NanoDrop 1000 spectrophotometer.

The NftE_1_ enzymatic assay was conducted in a 50 μL reaction system consisted of 1 mM compound **5** or **6** or **7** or **8**, 100 μg/mL ferredoxin (Fdx, spinach), 0.2 U/mL ferredoxin-NADP^+^ reductase (FdR, spinach), 10 U/mL glucose dehydrogenase, 10 mM glucose and 1 mM NADPH in 50 mM Tris-HCl buffer (pH 7.4). The modified NftE_1_ assay was carried out by using the alternative Fdx and FdR derived from cyanobacterium *Synechococcus elongatus* [23] with the recipe of 1 mM substrate, 10 μM Fdx, 10 μM FdR, 1 mM NADPH, 10 μM NftE_1_ in 50 mM PBS Buffer (pH7.5). The assay of NftF_1_ carried out in a 50 μL reaction system consisted of 1 mM Zn^2+^ or Mn^2+^ or Cu^2+^ or Mg^2+^, 10 μM NftF_1_, 0.5 mM compound **1** in 50 mM Tris-HCl buffer (pH6.8). All the reactions were incubated at 30 °C for 5 h, and halted by adding 500 μL of CH_3_CN before injecting 20 μL of the supernatant for HPLC–MS analysis.

### 2.9 Chemical complementation

Chemical complementation of Δ*nft*M_1_ strain was performed by adding 15-μL DMSO solution of compound **1**, **5** or **7** at time points of 2 d and 3 d during a 5-d fermentation. The concentration of stock solution was 0.2 M, and the final concentration of each compound added in the culture broth was 0.6 mM. As a negative control, the DMSO solvent was used in same condition. The fermentation was performed with 10-mL SGC medium in a 50-mL conical flask. The broth was extracted for HPLC–MS analysis after the 5-d fermentation.

## 3 Results and discussions

### 3.1 Discovery of compounds 1−4 and identification of the *nft* BGC from *S. conglobatus*

During screening of new metabolites from the mutant strain RJ2, which was generated by abolishing conglobatin production from *S. conglobatus* ATCC 31005 [19], we isolated four new components (compounds **1**−**4**) from a 12-liter fermentation extract. The structures of **1**−**4** were elucidated based on comprehensive analysis with the 1D and 2D NMR (Fig. 1 and Fig. S1). The structures of **1** and **2** bear a 2-pyrone ring and a 1, 2, 4-trisubstituted benzyl moiety, being structurally identical to TM-123/veramycin B (**1**) and veramycin A (**2**) that were recently discovered from *S. pactum* ATCC 27456 and *S.* sp. ST157608 [8,12] (Tables S3 and 4). The compound **3** is structurally identical to the trialkyl-substituted aromatic polyketide NFAT-133 reported from several *Streptomyces* species, which possesses antidiabetic and immunosuppressant activities [6,8,24] (Table S5). The compound **4** is the hydroxylated analog of **3**, being structurally identical to benwamycin I discovered from *S.* sp. KIB-H1471 [7] (Table S6).

Given the structural similarity of the trialkyl-substituted aromatic moiety in compounds **1**−**4** and the reported lorneic acids [25], we speculated a similar mechanism of the intramolecular benzene ring formation occurred in their biosynthesis. A genome mining was then performed toward *S. conglobatus* using the sequence of P450 monooxygenase homolog Orf1-257, which putatively carries out an oxidative aromatization of the polyolefinic precursor in the lorneic acids biosynthetic pathway [25]. The analysis revealed a BGC of type I PKS from *S. conglobatus* (GenBank OP948907), which contains a P450 monooxygenase homologous gene *nft*E_1_ showing 46% protein sequence identity to Orf1-257. The BGC has the same gene organization to the *nft* BGC and 77–91% identities of encoded proteins to the ones encoded in the *nft* BGC (Fig. S2), which was verified to account for the biosynthesis of compounds **1** and **3** in *S. pactum* [12].

### 3.2 Revising the PKS assembly line of 1−3 based on site-directed mutagenetic analysis of the DH domains in NftC_1_, NftK_1_ and NftD_1_

**Fig. 3:**
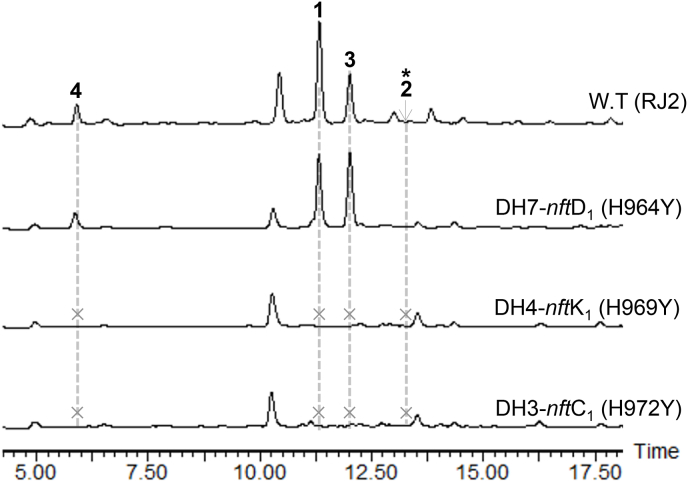
HPLC analysis of the metabolites of DH domain site-directed mutagenesis strains: DH3-*nft*C_1_ (H972Y), DH4-*nft*K_1_ (H969Y), and DH7-*nft*D_1_ (H964Y). The HPLC is displayed by using 250 nm. The asterisks mean the detected production of compound **2** by target mass extraction (see Fig. S5). The crosses mean that the target compounds were not detected.

Considering that the PKS assembly logic of NFAT-133 was interpreted inconsistently [8,12], we performed site-directed mutagenetic analysis of the DH domains in NftC_1_, NftK_1_ and NftD_1_, corresponding to the DH3, DH4 and DH7 in the PKS assembly line proposed by in the work (Fig. 2b). By replacing the His residues in their active sites with a Tyr in the conservative motif HX_3_GX_4_P of DHs (Fig. S3), the mutant strains DH3-*nft*C_1_ (H972Y), DH4-*nft*K_1_ (H969Y) and DH7-*nft*D_1_ (H964Y) were generated. According to HPLC–MS analysis of the fermentation extracts (Fig. 3), the DH3 and DH4 mutations resulted in complete abolishment of compounds **1**−**4**, while DH7 mutation did not affect the production of **1**−**4**. The results indicate that the DH domain in NftC_1_ should be active during the PKS assembly. This is contradicted to the previous speculation that the naturally silent DH domain in NftC_1_ led to the presence of C-7 hydroxyl group in the module-6 of PKS assembly line [8]. Similarly, the results support that the 10.13039/501100000276DH domain in NftK_1_ should be active too, and the NftK_1_ encoded module could serve as module-4 in the PKS assembly line (Fig. 2b). As expected, the activity of DH7 is not necessary in the PKS owing to that the KR7 domain should be inactive with a Y1532N mutation at the key active site of Tyr (Fig. S4).

Moreover, the double bound geometries are predicted by recognizing the diagnostic motifs of established KR types [26] (Fig. S4), in which KR4, KR5 and KR6 contain the LDD motif of B-type KRs correlating to *trans*-double bonds. Given that KR2 and KR3 lack the characteristic motifs of B-type KRs, we speculate that the *cis*-double bonds could be generated by KR2-DH2 and KR3-DH3 to facilitate the downstream aromatization. Taken together, these analyses allowed us to propose a revised PKS assembly line for **1**−**3** biosynthesis (Fig. 2b).

### 3.3 Identification of the essential genes from the *nft*E_1_−I_1_ gene cassette at the center of *nft* BGC

**Fig. 4:**
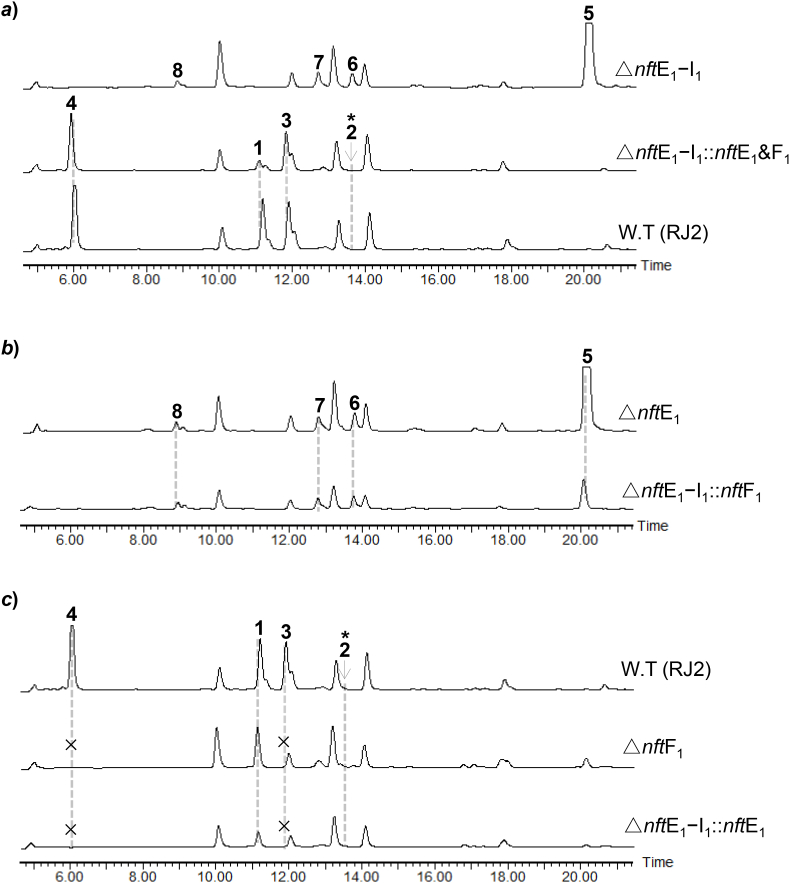
HPLC analysis of the metabolites from the mutant strains Δ*nft*E_1_−I_1_ (***a***), Δ*nft*E_1_ (***b***) and Δ*nft*F_1_ (***c***), and the complementary strains. The HPLC is displayed by using 250 nm. The asterisks mean the detected production of compound **2** by target mass extraction (see Fig. S5). The crosses mean that the target compounds were not detected.

To functionally identify the five non-PKS genes *nft*E_1_−I_1_ at the center of *nft* BGC (Fig. 2a), we first constructed a mutant by deleting the whole genes cassette in the staring strain RJ2. HPLC–MS analysis of the fermentation culture revealed that the resulting mutant Δ*nft*E_1_−I_1_ completely lost the capacity of producing **1**−**4**, and meanwhile four new components (compounds **5**−**8**) were detected (Fig. 4a). Next, to discriminate the redundant genes, complementation of the Δ*nft*E_1_−I_1_ mutant was performed. Surprisingly, the introduction of *nft*E_1_ and *nft*F_1_ genes was able to restore the production of **1**−**4** in Δ*nft*E_1_−I_1_ strain along with the disappearance of **5**−**8** (Fig. 4a), revealing the redundant genes of *nft*G_1_, *nft*H_1_ and *nft*I_1_. We then performed individual gene deletion towards *nft*E_1_ and *nft*F_1_. Interestingly, the resulting Δ*nft*E_1_ mutant produced the same metabolite profiles of Δ*nft*E_1_−I_1_ strains (Fig. 4b). The results indicate that the P450 monooxygenase-encoding gene *nft*E_1_ should involve in a common step for the biosynthesis of **1**−**4**, very likely the aromatic cyclization of a linear polyene chain offloaded from PKS. The proposal was supported by the recent report about the unusual cytochrome P450 monooxygenases LonP and GbnP, showing 46/91 and 46/97 identity%/coverage% to NftE_1_, which were verified to carry out the benzene ring formation in the biosynthesis of lorneic acids and gombapyrones [11]. Furthermore, the Δ*nft*F_1_ mutant lost the capacity of producing compounds **3** and **4** and still kept the production of **1** and **2** (Fig. 4c), indicating that *nft*F_1_ should specifically serve for generating compounds **3** and **4**.

The functions of *nft*E_1_ and *nft*F_1_ were further confirmed by individual gene complementation of the Δ*nft*E_1_−I_1_ mutant. As expected, introduction of *nft*F_1_ gene did not change the metabolic profile of the Δ*nft*E_1_−I_1_ strain (Fig. 4b), and introduction of *nft*E_1_ restored the production of **1** and **2** along with the absence of **3** and **4** in the culture of Δ*nft*E_1_−I_1_ :: *nft*E_1_ strain (Fig. 4c). Additionally, Zhou et al. recently reported that deletion of the putative decarboxylase gene *nft*G resulted in significantly reduced amount of compound **3** [27]. However, in-frame deletion of the cognate gene *nft*G_1_ in our strain showed no influence to the production of **1**−**4** (Fig. S6), suggesting that *nft*G_1_ should be redundant or an alternative factor encoded by the gene outside of *nft* BGC could bear the same activity of NftG_1_ in the RJ2 strain.

### 3.4 Structural characterization of the compounds 5−8 produced by Δ*nft*E_1_ mutant strain

To characterize the structures of compounds **5**−**8** by spectral analysis, a 12-liter liquid fermentation of Δ*nft*E_1_ strain was performed to isolate 32 mg of **5** (white crystal), 5.9 mg of **6** (yellow amorphous powder), 19.4 mg of **7** (pale yellow amorphous powder), and 37.5 mg of **8** (white amorphous powder) though column chromatography and preparative HPLC.

**Fig. 5:**
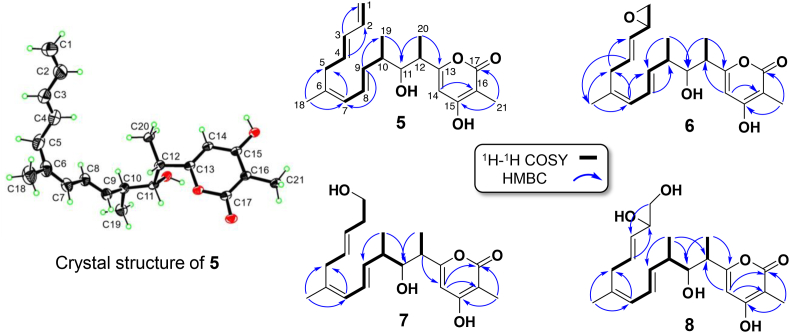
ORTEP drawing of **5**, and the ^1^H–^1^H COSY and key HMBC correlations of **5**–**8**.

Compound **5** was determined with the molecular formula C_21_H_28_O_4_ by HR-ESIMS (*m*/*z* 367.1883 [M + Na]^+^, calcd for C_21_H_28_O_4_Na, 367.1880, error 0.8 ppm), displaying 8° of unsaturation. The ^13^C NMR and DEPT spectra of **5** revealed 21 carbon resonances, including five quaternary carbons (*δ*_C_ 165.4, 165.2, 164.9, 134.3 and 96.7), ten methine carbons (*δ*_C_ 137.1, 135.4, 132.1, 131.7, 126.2, 125.8, 99.2, 74.5, 40.9, 40.8), two methylene carbons (*δ*_C_ 115.7, 35.0), and four methyl carbons (*δ*_C_ 23.5, 17.1, 10.8, 8.4) (Table S7). The ^1^H–^1^H COSY of **5** reveals four spin systems (Fig. 5). The presence of *α*-pyrone ring in **5** is confirmed through the HMBC correlations between H-14 and C-16 as well as between H-21 and C-15/C-17 (Table S7, Fig. 5). A long aliphatic chain of 15 carbons is established by the HMBC correlations from H-3 to C-1, from H-4 to C-2, from H-18 to C-5/C-7, from H-7 to C-5, from H-9 to C-7/C-19, from H-19 to C-11, and from H-20 to C-11/C-13 (Table S7, Fig. 5). The positions of substituted methyl and aliphatic chain at the *α*-pyrone ring were confirmed based on the HMBC correlations between H-20 and C-13 as well as between H-21 and C-15/C-17 (Table S7, Fig. 5). Consequently, compound **5** was established as a methyl substituted *α*-pyrone ring linking with a 15-carbons polyene chain, which is identical to the planar structure of TM-125 discovered during our investigation by Zhou et al. from another Δ*nft*E mutant generated in *S. pactum* [12]. Moreover, the absolute structure of **5** was first determined in the work by using single-crystal X-ray diffraction (Fig. 5).

The compounds **6**−**8** were determined with the molecular formulas C_21_H_28_O_5,_ C_21_H_30_O_5_, and C_21_H_30_O_6_, corresponding to the unsaturation degrees of 8, 7 and 7, respectively, based on the HR-ESIMS analysis: **6** (*m*/*z* 383.1841 [M + Na]^+^, calcd for C_21_H_28_O_5_Na, 383.1829, error 3.1 ppm), **7** (*m*/*z* 385.1983 [M + Na]^+^, calcd for C_21_H_30_O_5_Na, 385.1985, error −0.5 ppm), and **8** (*m*/*z* 401.1928 [M + Na]^+^, calcd for C_21_H_30_O_6_Na, 401.1935, error 1.5 ppm). The 1D and 2D NMR data suggests that compounds **6**−**8** should be the structural derivatives of **5**, differing by the terminal epoxide ring in **6**, the C-1 hydroxyl in **7**, and the 1, 2-*ortho* hydroxyls in **8** (Fig. 5). Compared to **5**, the upfield shifts of C-1 (Δ*δ* −51.2) and of C-2 (Δ*δ* −56.8) were observed in **6** (Table S8), and one more oxygen should be possessed by **6** according to the determined molecular formula, suggesting a C-1−C-2 epoxy ring in **6**. The C-1 hydroxyl in **7** could be deduced from the downfield shift resonance of C-1 (*δ*_C_ 60.9) and C-2 (*δ*_C_ 35.9) (Table S9). Compared to **7**, the downfield shift of C-2 (Δ*δ* +35.1) along with one more oxygen in the determined molecular formula suggest the 1, 2-*ortho* hydroxyls in **8** (Table S10). These elucidations were further confirmed by 2D NMR analysis of **6**−**8** (Fig. 5, Tables S7–10).

### 3.5 Exploration of the NftE_1_-mediate polyketide aromatic cyclization

The non-aromatic structure of compounds **5**−**8** produced by the Δ*nft*E_1_ mutant suggests that the P450 monooxygenase homologous gene *nft*E_1_ indeed involved in the aromatization process during the **1**−**4** biosynthesis (Fig. 2b). To investigate the *in vitro* activity of NftE_1_, we overexpressed the soluble protein of *C*-His_6_-fused NftE_1_ in *E. coli* BL21 (DE3) and purified the homogeneous protein by using Ni-NTA chromatography (Fig. S7). However, no new product was generated from the assay consisting of 10 μM NftE_1_, 1 mM compound **5** or **6** or **7** or **8**, 100 μg/mL ferredoxin (Fdx, spinach), 0.2 U/mL ferredoxin-NADP^+^ reductase (FdR, spinach), 10 U/mL glucose dehydrogenase, 10 mM glucose, and 1 mM NADPH in 50 mM Tris.HCl Buffer (pH 7.4) at 30 °C for 5 h. During our investigation, Yang et al. confirmed that the cytochrome P450 LonP/Orf1-257 in lorneic acids biosynthesis catalyzes the biotransformation of an acyclic polyene substrate to the benzene ring [11]. As such, we re-investigated the activity of NftE_1_ by using the alternative Fdx and FdR derived from cyanobacterium *Synechococcus elongatus* [23]. However, the modified assay still did not provide desirable products. We speculate that the authentic substrate for NftE_1_ could be the intermediate int-A2 (Fig. 2b), which could be converted into compound **5** upon the absence of NftE_1_ by receiving a *trans*-*cis* isomerization at C14–C15 double (Fig. 2c). The proposal may explain the unable restoration of compound **1** through chemical complementation of the Δ*nft*M_1_ strain with each of compounds **5** and **7** (data not shown).

### 3.6 Functional identification of *nft*F_1_ gene in producing 3 and 4

The *nft*F_1_ gene, showing homologous to MBL-fold hydrolase, was originally annotated as the conventional resistance gene of secondary metabolite BGCs since that metallo-hydrolases usually confer host resistance by hydrolyzing *β*-lactam antibiotics [28]. Surprisingly, gene deletion mutation and complementation revealed that *nft*F_1_ gene was essential for the production of compounds **3** and **4**, and uncorrelated to compounds **1** and **2** (Fig. 4c). Owing to the truncated structure feature of **3** compared to **1**, we hypothesized that compound **3** could be derived from either a heptaketide chain prematurely offloaded from ACP7 or an NftF_1_-hydrolyzed product of compound **1** (Fig. 2b). With the recombinant protein of *C*-His_6_-fused NftF_1_ expressed in *E. coli* BL21 (DE3) (Fig. S7), we first tested the hydrolysis ability of NftF_1_ towards the lactone ring of **1**. However, no new product was detected in the assay consisting of 10 μM NftF_1_, 0.5 mM compound **1**, 50 mM Tris.HCl buffer (pH6.8), and 1 mM divalent metal ions of Zn^2+^ or Mn^2+^ or Cu^2+^ or Mg^2+^. Moreover, we evaluated the hydrolytic activity of NftF_1_ towards compound **1** by feeding, with **1**, the fermentation of the Δ*nft*M_1_ strain which lost the production of **1**−**4** due to deletion of the PKS gene *nft*M_1_. However, no restoration of compound **3** was detected in the fermentation fed with compound **1** (Fig. S8). As such, it is rather likely that NftF_1_ contains TE activity to specifically offload a nascent chain from ACP7 for producing the compound **3** (Fig. 2b).

Protein BLAST analysis of NftF_1_ reveals a widespread MBL-fold hydrolase homolog from the BGCs of type II PKS, which were generally annotated as aromatic ring cyclases due to their non-similarities to any typical TE. Protein sequence alignment of the selected homologs from the documented type II PKS BGCs enables us to recognize the conserved THxHxDH motif and metal-binding sites from MBL family (Fig. S9). Of them, two MBL-fold hydrolases were functionally characterized with TE activities in type II PKSs, including ActIV (GenBank: CAC44204.1) and MrqD (GenBank: AQW35062.1) which show 38% identity (90% query-cover) and 31% identity (95% query-cover) to NftF_1_, respectively. ActIV was characterized as a bifunctional cyclase-thioesterase via *in vitro* reconstitution of actinorhodin biosynthesis pathway, and it was also demonstrated with TE activity towards a model substrate of type II PKS, anthraquinone-2-carboxylic acid-*N*-acetylcysteamine [14]. MrqD was suggested to function as a dedicated product releasing TE in murayaquinone biosynthesis based on interpretations of the gene deletion and site-directed mutagenetic analysis [15]. Thus, these bioinfomatic informations imply the TE activity of NftF_1_. However, more experimental evidences are required to exclude another possible mechanism, in which, with yet unidentified cofactor, NftF_1_ hydrolyzes the lactone-bond of **1** to yield compound **3** (Fig. 2b). Further investigation will be performed to determine the exact mechanism of NftF_1_ in producing the compound **3** such as chemically synthesis of the mimic substrate of *N*-acetylcysteaminyl thioester for analyzing the TE activity of NftF_1_ or screening of the cofactors of NftF_1_ in hydrolytic degradation of compound **1**.

## 4 Conclusion

The PKS assembly line of trialkyl-substituted aromatic polyketides **1**−**4** is revised based on site-directed mutagenetic analysis of the three DH domains. The *nft*E_1_ and *nft*F_1_ genes were identified to be essential for **1**−**4** biosynthesis through gene deletion and complementation analysis of the *nft*E_1_−I_1_ gene cassette at the center of *nft* BGC. Structure elucidation of the new products **5**−**8** accumulated in the Δ*nft*E_1_ mutant suggests that the unusual benzene core formation in **1**−**4** should be controlled by the P450 monooxygenase NftE_1_. Gene deletion and complementation reveal that *nft*F_1_ gene is essential to produce **3** and **4**. Protein BLAST analysis reveals that NftF_1_ belongs to MBL-fold hydrolase family. The homologs of NftF_1_ are widespread in type II PKSs, in which two homologs were demonstrated with TE activities. We speculate that NftF_1_ could serve as a *trans*-acting TE to mediate a premature chain-offloading from ACP7 yielding the precursor chain of compound **3**. Alternatively, with yet unidentified cofactors, NftF_1_ is capable to hydrolyze the lactone-bond of **1** for yielding the compound **3**.

## Funding

This work received financial support from the 10.13039/501100001809National Natural Science Foundation of China (Nos. 32070070, 32211530074 and 31929001), and the innovative research team of high-level local universities in Shanghai. H. D. thanks Royal Society-NSFC international exchange grant (IEC\NSFC\211349).

## CRediT authorship contribution statement

**Ming Yang:** Data curation, Formal analysis, Investigation, Visualization, Writing – original draft. **Wanlu Li:** Data curation, Formal analysis, Investigation, Visualization. **Lin Zhou:** Investigation. **Xiao Lin:** Investigation. **Wenyu Zhang:** Writing – review & editing. **Yaoyao Shen:** Investigation. **Hai Deng:** Writing – review & editing. **Hou-wen Lin:** Funding acquisition, Resources, Supervision. **Yongjun Zhou:** Conceptualization, Funding acquisition, Methodology, Project administration, Resources, Supervision, Validation, Writing – original draft, Writing – review & editing.

## Declaration of competing interest

Authors declare that they have no conflict of interest.
