# Supplementary material for: Biosynthesis of trialkyl-substituted aromatic polyketide NFAT-133 involves unusual P450 monooxygenase-mediating aromatization and a putative metallo-beta-lactamase fold hydrolase
Source: Synth Syst Biotechnol. 2023 Jun 3;8(3):349–56. doi: 10.1016/j.synbio.2023.05.003 (PMC10265476; doi:10.1016/j.synbio.2023.05.003)

**SUPPORTING INFORMATION**

**Biosynthesis of trialkyl-substituted aromatic polyketide NFAT-133 involves unusual P450 monooxygenase-mediating aromatization and a putative metallo-beta-lactamase fold hydrolase**

**Section-I. Tables**

**Table S1.** Primers used in this study

**Table S2.** Plasmids constructed in this study

**Table S3.** Comparison of experimental and reported ^1^H and ^13^C NMR data of **1**

**Table S4.** Comparison of experimental and reported ^1^H and ^13^C NMR data of **2**

**Table S5.** Comparison of experimental and reported ^1^H and ^13^C NMR data of **3**

**Table S6.** Comparison of experimental and reported ^1^H and ^13^C NMR data of **4**

**Table S7.** ^1^H and ^13^C NMR data for **5** in DMSO-*d*_6_

**Table S8.** ^1^H and ^13^C NMR data for **6** in DMSO-*d*_6_

**Table S9.** ^1^H and ^13^C NMR data for **7** in DMSO-*d*_6_

**Table S10.** ^1^H and ^13^C NMR data for **8** in DMSO-*d*_6_

**Section-II. Figs**

**Fig. S1.** ^1^H-^1^H COSY and key HMBC correlations of **1** and **2**

**Fig. S2.** Comparison of the *nft* BGCs derived from *S. conglobatus* and *S. pactum*

**Fig. S3.** Protein sequence alignment of the DH domains of the *nft* BGC from *S. conglobatus*.

**Fig. S4.** Protein sequence alignment of the KR domains of the *nft* BGC from *S. conglobatus*.

**Fig. S5.** HPLC–MS analysis of the production of compound **2** in mutant strains.

**Fig. S6.** HPLC–MS analysis of the culture extract of Δ*nft*G_1_ mutant strain.

**Fig. S7.** SDS-PAGE analysis of the recombinant proteins NftE_1_ and NftF_1_.

**Fig. S8.** HPLC analysis of the culture extract of Δ*nft*M_1_ mutant strain fed with compound **1** and DMSO.

**Fig. S9.** Protein sequence alignment of NftF_1_, Chd (PDB 6UXU) and its homologous proteins from the reported BGCs of type II PKS.

**Fig. S10.** NMR spectra of **1** (related to Table S3)

**Fig. S11.** NMR spectra of **2** (related to Table S4)

**Fig. S12.** NMR spectra of **3** (related to Table S5)

**Fig. S13.** NMR spectra of **4** (related to Table S6)

**Fig. S14.** NMR spectra of **5** (related to Table S7)

**Fig. S15.** NMR spectra of **6** (related to Table S8)

**Fig. S16.** NMR spectra of **7** (related to Table S9)

**Fig. S17.** NMR spectra of **8** (related to Table S10)

**Section-I. Tables**

**Table S1.** Primers used in this study.

| **Primers Sequence (5’ to 3’)** **Descriptions** | | |
| --- | --- | --- |
| **DH3-*nft*C_1_ (H972Y) site-directed mutagenesis by generating pRJ277** | | |
| L-DH4-S | ATCCCCGGGGACCTGCAGGTCGACTGCCACGTGATCCTGGAG  (25 bp overlapping with the *Xba*I side of pRJ2) | Left arm of homologous recombination |
| L-DH4-A | ATGCCGAAGACCACGTAGTCTCGGATCCACGGCTGGGAG  (34 bp overlapping with the right arm) |  |
| R-DH4-S | AGCCGTGGATCCGAGACTACGTGGTCTTCGGCATCCCCATC | Right arm of homologous recombination |
| R-DH4-A | TATCACGAGGCCCTTTCGTCTTCAAGGCGCCCGAGGAGAACAGCAC  (26 bp overlapping with the *Eco*RI side of pRJ2) |  |
| T-DH4-S | ACGGTGGACTGGTCGGCGTTCTAC | Screening of the mutation |
| T-DH4-A | AGCCCGTCTCGTCGTCGAGGAC |  |
| **DH4-*nft*K_1_ (H969Y) site-directed mutagenesis by generating pRJ278** | | |
| L-DH5-S | ATCCCCGGGGACCTGCAGGTCGACTGGACATCGGCTTCTCCTC  (25 bp overlapping with the *Xba*I side of pRJ2) | Left arm of homologous recombination |
| L-DH5-A | TGCCGAAGACCATGTAGTCACGGGTCCACGGCTGCGCGTC  (28 bp overlapping with the right arm) |  |
| R-DH5-S | TGGACCCGTGACTACATGGTCTTCGGCACGGTGCTG | Right arm of homologous recombination |
| R-DH5-A | TATCACGAGGCCCTTTCGTCTTCAAGCACGTCCCGGAAGTTCAG  (26 bp overlapping with the *Eco*RI side of pRJ2) |  |
| T-DH5-S | TCACGGTGGACTGGCCGGAAC | Screening of the mutation |
| T-DH5-A | AGCACCAGCTCCTCCAGCACCTC |  |
| **DH7-*nft*D_1_ (H964Y) site-directed mutagenesis by generating pRJ279** | | |
| L-DH6-S | ATCCCCGGGGACCTGCAGGTCGACTACCAACGTGCACGTCATCCTG  (25 bp overlapping with the *Xba*I side of pRJ2) | Left arm of homologous recombination |
| L-DH6-A | TGCGCCCCGAGAACGACGTAATCGGCGATCCATGGCTGCGTG  (37 bp overlapping with the right arm) |  |
| R-DH6-S | AGCCATGGATCGCCGATTACGTCGTTCTCGGGGCGCAGATG | Right arm of homologous recombination |
| R-DH6-A2 | TATCACGAGGCCCTTTCGTCTTCAAGACCGTCCACCGAGGACAGCACGTC  (26 bp overlapping with the *Eco*RI side of pRJ2) |  |
| T-DH6-S | ATGTCGACTGGACGGCCTACTAC | Screening of the mutation |
| T-DH6-A | TTGTCGGTGAGGACCACCTG |  |
| **Deletion of the *nft*E_1_-I_1_ gene cassette (Δ*nft*E_1_-I_1_) by generating pRJ54** | | |
| Lmd-SS | ATCCCCGGGGACCTGCAGGTCGACTCGTCTGCGTGCCGTCGTTC  (24 bp overlapping with the *Xba*I side of pRJ2) | Left arm of homologous recombination |
| Lmd-AA | AGCCCTGATCACCACACCACACCGTGATCTGCTCCACCGCTTC  (36 bp overlapping with the right arm) |  |
| Rmd-SS | TGGAGCAGATCACGGTGTGGTGTGGTGATCAGGGCT | Right arm of homologous recombination |
| Rmd-A | TATCACGAGGCCCTTTCGTCTTCAAGTGTTGTCGACTGCTCTCATTCC  (25 bp overlapping with the *Eco*RI side of pRJ2) |  |
| T-RJ13-S | ACTTCCTCAACGAGTGCAAGC | The Δ*nft*E_1_-I_1_ mutant screening |
| T-RJ13-A | TATATCGCTCGGGCAGTGGATTC |  |
| **Complementation of the Δ*nft*E_1_-I_1_ mutant** | | |
| RJ13-CS | AATCGTGCCGGTTGGTAGGATCCACATATGAACCCTCCCGACTCGAACGAC | Cloning of *nft*E_1_-F_1_ gene based on the pRJ5 (*Nde*I, *Eco*RV) to produce pRJ62 |
| RJ13-CA | AAACAGCTATGACATGATTACGAATTCGATATCATGCCGGCACCCTGGTTTCC |  |
| **In-frame deletion of *nft*G_1_ (Δ*nft*G_1_) by generating pRJ11** | | |
| dd-L-S | ATCCCCGGGGACCTGCAGGTCGACTCCACGAGATCAAGGTCATG  (25 bp overlapping with the *Xba*I side of pRJ2) | Left arm of homologous recombination |
| dd-L-A | ATGTAGGCGAGCATCGAGTAGACCATGTGCGACAGAGGACCGT |  |
| dd-R-S | TGTCGCACATGGTCTACTCGATGCTCGCCTACATCCTG | Right arm of homologous recombination |
| dd-R-A | TATCACGAGGCCCTTTCGTCTTCAAGATCGCTGAGGTACATC  (29 bp overlapping with the *Eco*RI end of pRJ2) |  |
| cpy-3S | ATCGGGTTCAACGGGGAACTG | The Δ*nft*G_1_ mutant screening |
| cpy-2A | ATCGCAGGACTTCAGGATGTAG |  |
| **In-frame deletion of *nft*E_1_ (Δ*nft*E_1_) by generating pRJ141** | | |
| L-cpy3-S | ACCGGGACTGATCAAGGCGAATACTATCAAGAAGAGCGACTTCGCGGAGCTGGTG  (25 bp overlapping with the *Xba*I side of pRJ2) | Left arm of homologous recombination |
| L-cpy3-A | TTGTTCATCAGCTCCACCGTGATCTGCTCCAC  (31 bp overlapping with the right arm) |  |
| R-cpy3-S | TGGAGCAGATCACGGTGGAGCTGATGAACAACGACC | Right arm of homologous recombination |
| R-cpy3-A | TATCACGAGGCCCTTTCGTCTTCAAGGAAACAGCTATGACATGATTAC  (29 bp overlapping with the *Eco*RI end of pRJ2) |  |
| T-cpy3-S | ACTTCCTCAACGAGTGCAAG | The Δ*nft*E_1_ mutant screening |
| T-cpy3-A | GACATGACTTTCTCCTTGTTCAC |  |
| **Complementation of the Δ*nft*E_1_ mutant** | | |
| KasOp-S | TGTAAAACGACGGCCAGTGCCAAGCTTGGGCTG  (49 bp overlapping with the *Nsi*I side of pRJ5) | Cloning of the *nft*E_1_ gene ORF and the *KasO**P promoter based on pRJ5 (*Nsi*I, *Eco*RV) to produce pRJ148 |
| KasO-cgp3-A | TTCGAGTCGGGAGGGTTCATAACTCCCCCAGTCCTG  (42 bp overlapping with the second part of the gene) |  |
| cgp3-KasO-S | AGCGTGCAGGACTGGGGGAGTTATGAACCCTCCCGACTCGAAC |  |
| RJ13-CAA | AACAGCTATGACATGATTACGAATTCGATATCACATGAAATTCCGCGGTCAGCTG  (30 bp overlapping with the *Eco*RV side of pRJ5) |  |
| CRJ13-S | TGCTCGAAGTCAACACCTAC | Identification of the presence of *nft*E_1_ |
| CRJ13-A | ATGACCTTGATCTCGTGGATC |  |
| **In-frame deletion of *nft*F_1_ (Δ*nft*F_1_) by generating pRJ142** | | |
| L-cpy4-S | ACCGGGACTGATCAAGGCGAATACTTCAATGGTGGTTCTCATTCCTTGGTTACG  (25 bp overlapping with the *Xba*I side of pRJ2) | Left arm of homologous recombination |
| L-cpy4-A | AGTCAAACTCGGTGAAGGGAACAGACATGACTTTCTCCTTGTTCACATG  (33 bp overlapping with the right arm) |  |
| R-cpy4-S | AAGTCATGTCTGTTCCCTTCACCGAGTTTGACTTCGGCGAGTTCTC | Right arm of homologous recombination |
| R-cpy4-A | TATCACGAGGCCCTTTCGTCTTCAAGTCGTAGTGCTCCGACAG  (26 bp overlapping with the *Eco*RI side of pRJ2) |  |
| T-cpy4-S | ATCCACGAGATCAAGGTCATGCTG | The Δ*nft*F_1_ mutant screening |
| T-cpy4-A | TTCAGATCCACCATCTGGTGCAG |  |
| **Complementation of the Δ*nft*F_1_ mutant** | | |
| KasOp-S | Same as above | Cloning of the *nft*F_1_ gene ORF and the *KasO**p promoter based on pRJ5 (*Nsi*I, *Eco*RV) to produce pRJ149 |
| KasO-4-A | TGGTGAAGGGAACAGACATAACTCCCCCAGTCCTGCAC  (43 bp overlapping with the second part of the gene) |  |
| 4-KasO-S | ACAGCGTGCAGGACTGGGGGAGTTATGTCTGTTCCCTTCACCAC |  |
| RJ13-CA | AAACAGCTATGACATGATTACGAATTCGATATCATGCCGGCACCCTGGTTTCC  (30 bp overlapping with the *Eco*RV side of pRJ5) |  |
| T-KasO-S | TTGTAAAGTCGTGGCCAGGAG | Identification of the presence of *nft*F_1_ or its mutations |
| PIB139-A | TGAGTTAGCTCACTCATTAGGCAC |  |
| **In-frame deletion of *nft*M_1_ (Δ*nft*M_1_) by generating pRJ14** | | |
| cpyA-L-S | ATCCCCGGGGACCTGCAGGTCGACTGATCTTCGACCATCCGACAC  (25 bp overlapping with the *Xba*I side of pRJ2) | Left arm of homologous recombination |
| cpyA-L-A | ATGAGGTTCTCGACGTCCAAGGCAACGGCTCGGTCGTTCATC  (38 bp overlapping with the right arm) |  |
| cpyA-R-S | AACGACCGAGCCGTTGCCTTGGACGTCGAGAACCTCATC | Right arm of homologous recombination |
| cpyA-R-A | TATCACGAGGCCCTTTCGTCTTCAAGACGATGGAGACAGGCGAGAC  (26 bp overlapping with the *Eco*RI side of pRJ2) |  |
| cpyA-T-S | TCATTCGGGAACGGGGAGATC | Screening of the mutation |
| cpy-7A | ACGATCTCGACGCTGTCCTCAAG |  |
| **NftE_1_ protein expression** | | |
| P-cgp3-S | ACTTTAAGAAGGAGATATACATATGAACCCTCCCGACTCGAACGACG  (22 bp overlapping with *Nde*I side of pET29a) | Cloning of *nft*E_1_ gene with C-terminal 6×His tag based on pET-29a (*Nde*l, *Xho*l) to produce pRJ148 |
| P-cgp3-A | TCAGTGGTGGTGGTGGTGGTGCTCAGTGGTGGTGGTGGTGGTGGCTGCCGACCGCGACCGC  (22 bp overlapping with *Xho*l side of pET29a) |  |
| **Protein expression of NftF_1_** | | |
| P-cgp4-S | ACTTTAAGAAGGAGATATACATATGTCTGTTCCCTTCACCACCGGTGTG  (22 bp overlapping with *Nde*I side of pET29a) | Cloning of *nft*F_1_ gene with C-terminal 6×His tag based on pET-29a (*Nde*l, *Xho*l) to produce pRJ149 |
| P-cgp4-A | TCAGTGGTGGTGGTGGTGGTGCTCAGTGGTGGTGGTGGTGGTGTGCCGGCACCCTGGTTTCCAGGAC(22 bp overlapping with *Xho*l side of pET29a) |  |

**Table S2.** Plasmids constructed in this study.

| Plasmids | Resistance | Descriptions |
| --- | --- | --- |
| pRJ11 | hygR, ampR | to generate *nft*G_1_ in-frame deletion |
| pRJ14 | hygR, ampR | to generate *nft*M_1_ in-frame deletion |
| pRJ54 | hygR, ampR | to generate *nft*E_1_-I_1_ in-frame deletion |
| pRJ62 | hygR | cloning of *nft*E_1_-F_1_ under *ermE*p* |
| pRJ141 | hygR, ampR | to generate *nft*E_1_ in-frame deletion |
| pRJ142 | hygR, ampR | to generate *nft*F_1_ in-frame deletion |
| pRJ148 | hygR | cloning of *nft*E_1_ under *KasO**p |
| pRJ149 | hygR | cloning of *nft*F_1_ under *KasO**p |
| pRJ266 | kanaR | NftE_1_ protein express, based on pET-29a (NdeI, Xhol) |
| pRJ268 | kanaR | NftF_1_ protein express, based on pET-29a (NdeI, Xhol) |
| pRJ277 | hygR, ampR | to generate *nft*C_1_*-*DH3 mutation (H972Y) |
| pRJ278 | hygR, ampR | to generate *nft*K_1_*-*DH4 mutation (H969Y) |
| pRJ279 | hygR, ampR | to generate *nft*D_1_*-*DH7 mutation (H964Y) |
| Abbreviation: amp, ampicillin; kana, kanamycin; hyg, hygromycin | | |

| **Table S3.** Comparison of experimental and reported ^1^H and ^13^C NMR data of **1** | | | | |
| --- | --- | --- | --- | --- |
| No. | Experimental *δ*_C_ (150 MHz) | Literature^a^  *δ*_C_ (125 MHz) | Experimental *δ*_H,_ mult. (600 MHz. *J* in Hz) | Literature^a^  *δ*_H,_ mult. (500 MHz. *J* in Hz) |
| 1 | 61.8 | 62.4 | 4.09, m | 4.2, d (6) |
| 2 | 133.0 | 131.2 | 6.11, dt (15.6, 5.4) | 6.13, dt (6, 16) |
| 3 | 127.0 | 128.1 | 6.72, brd (15.6) | 6.82, d (16) |
| 4 | 135.0 | 135.49 |  |  |
| 5 | 128.3 | 127.1 | 7.19, brs | 7.20, s |
| 6 | 135.2 | 135.5 |  |  |
| 7 | 127.1 | 128.2 | 7.03, dd (7.95, 1.2) | 7.02, d (8.0) |
| 8 | 126.1 | 126.9 | 7.11, d (7.95) | 7.15, d (8.0) |
| 9 | 139.3 | 138.8 |  |  |
| 10 | 39.2 | 38.3 | 2.97, dq (6.9, 6.9) | 3.09, m |
| 11 | 74.9 | 75.7 | 3.79, dd (6.4, 6.4) | 3.96, dd (6, 8) |
| 12 | 40.8 | 41.7 | 2.31, dq (6.7, 6.7) | 2.48, m |
| 13 | 165.2 | 165.5 |  |  |
| 14 | 99.5 | 99.7 | 5.94 | 5.89 |
| 15 | 165.4 | 167.5 |  |  |
| 16 | 96.7 | 97.8 |  |  |
| 17 | 165.5 | 166.3 |  |  |
| 18 | 20.7 | 19.6 | 2.24, s | 2.27, s |
| 19 | 17.9 | 16.8 | 1.18, d (6.9) | 1.27, d (7) |
| 20 | 11.8 | 11.8 | 0.98, d (6.9) | 1.14, d (7) |
| 21 | 8.5 | 6.8 | 1.73, s | 1.82, s |
| *^a^* ACS Chem. Biol. 2020, 15, 12, 3217–3226 | | | | |

| **Table S4.** Comparison of experimental and reported ^1^H and ^13^C NMR data of **2** | | | | |
| --- | --- | --- | --- | --- |
| No. | Experimental *δ*_C_ (150 MHz) | Literature^a^  *δ*_C_ (175 MHz) | Experimental *δ*_H,_ mult. (600 MHz. *J* in Hz) | Literature^a^  *δ*_H,_ mult. (700 MHz. *J* in Hz) |
| 1 | 63.3 | 63.8 | 4.17, m | 4.23, ddd (5.6, 2.7, 1.6) |
| 2 | 133.4 | 132.8 | 6.14, dt (15.5, 5.4) | 6.13, dt (15.6, 5.6) |
| 3 | 128.5 | 129.5 | 6.80, dd (15.5, 1.8) | 6.86, dt (15.6, 1.6) |
| 4 | 136.8 | 136.9 |  |  |
| 5 | 128.4 | 128.5 | 7.22, d (1.8) | 7.20, d (1.6) |
| 6 | 136.6 | 136.9 |  |  |
| 7 | 129.1 | 129.6 | 7.05, dd (7.9, 1.8) | 7.02, d (7.9, 1.6) |
| 8 | 128.5 | 128.3 | 7.14, d (7.9) | 7.13, d (7.9) |
| 9 | 140.1 | 140.2 |  |  |
| 10 | 38.9 | 39.8 | 3.06, m | 3.07, dq (7.8, 6.9) |
| 11 | 76.2 | 77.1 | 3.86, t (6.3) | 3.99, dd (7.8, 5.6) |
| 12 | 42.7 | 43.1 | 2.45, m | 2.46, dq (7.0, 5.6) |
| 13 | 166.4 | 167.2 |  |  |
| 14 | 100.5 | 101.3 | 5.97, s | 5.88, s |
| 15 | 165.4 | 167.6 |  |  |
| 16 | 105.1 | 105.3 |  |  |
| 17 | 166.5 | 168.6 |  |  |
| 18 | 20.9 | 21.1 | 2.28, s | 2.26 s |
| 19 | 16.4 | 18.5 | 1.23, d (6.9) | 1.27, d (6.9) |
| 20 | 13.7 | 13.0 | 1.10, d (6.9) | 1.12, d (7.0) |
| 21 | 17.2 | 17.3 | 2.35, q (7.4) | 2.36, q (7.4) |
| 22 | 12.8 | 12.8 | 1.02, t (7.4) | 1.02, t (7.4) |
| *^a^* Org. Chem. Front., 2022, 9, 1604–1615 | | | | |

| **Table S5.** Comparison of experimental and reported ^1^H and ^13^C NMR data of **3** | | | | |
| --- | --- | --- | --- | --- |
| No. | Experimental *δ*_C_ (150 MHz) | Literature^a^  *δ*_C_ (125 MHz) | Experimental *δ*_H,_ mult. (600 MHz. *J* in Hz) | Literature^a^  *δ*_H,_ mult. (500 MHz. *J* in Hz) |
| 1 | 63.7 | 63.7 | 4.33, brd (5.4) | 4.35, d (5.4) |
| 2 | 131.7 | 131.5 | 6.17, dt (15.6, 5.4) | 6.19, dt (15.6, 5.4) |
| 3 | 128.8 | 128.8 | 6.92, d (15.7) | 6.93, d (15.6) |
| 4 | 136.1 | 136.0 |  |  |
| 5 | 128.0 | 127.9 | 7.23, d (1.6) | 7.24, s |
| 6 | 135.9 | 135.8 |  |  |
| 7 | 128.6 | 128.6 | 7.03-7.06, m | 7.05, d (8.0) |
| 8 | 126.6 | 126.5 | 7.03-7.06, m | 7.07, d (8.0) |
| 9 | 138.6 | 138.5 |  |  |
| 10 | 37.4 | 37.3 | 3.09, p (7.0) | 3.11, dp (7.8, 6.9) |
| 11 | 74.7 | 74.5 | 4.10, dd (7.9, 3.9) | 4.11, dd (7.8, 4.0) |
| 12 | 48.6 | 48.5 | 2.46, m | 2.48, dq (4.0, 7.2) |
| 13 | 214.3 | 214.2 |  |  |
| 14 | 29.3 | 29.3 | 2.11, s | 2.13, s |
| 15 | 21.1 | 21.0 | 2.32, s | 2.33, s |
| 16 | 17.7 | 17.5 | 1.30, d (6.8) | 1.32, d (6.9) |
| 17 | 10.7 | 10.7 | 1.05, d (7.2) | 1.08, d (7.2) |
| ***^a^*** *J Antibiot (Tokyo).* 2016, 69: 69-71 | | | | |

| **Table S6.** Comparison of experimental and reported ^1^H and ^13^C NMR data of **4** | | | | |
| --- | --- | --- | --- | --- |
| No. | Experimental *δ*_C_ (150 MHz) | Literature^a^ *δ*_C_ (150 MHz) | Experimental *δ*_H,_ mult. (600 MHz. *J* in Hz) | Literature^a^  *δ*_H,_ mult. (600 MHz. *J* in Hz) |
| 1 | 61.6 | 62.2 | 4.15, dd (4.9, 1.8) | 4.14, brs |
| 2 | 132.9 | 133.2 | 6.16, dt (15.6, 4.9) | 6.16, dt (15.7,5.0) |
| 3 | 125.9 | 126.6 | 6.89, dt (15.7, 2.0) | 6.89, d (15.7) |
| 4 | 135.1 | 135.7 |  |  |
| 5 | 124.6 | 125.2 | 7.37, d (1.9) | 7.37, brs |
| 6 | 140.1 | 140.4 |  |  |
| 7 | 125.9 | 126.7 | 7.18, dd (8.0, 1.9) | 7.18, dd (8.0,1.2) |
| 8 | 126.7 | 127.3 | 7.25 (d, 8.0) | 7.25, d (8.0) |
| 9 | 140.6 | 141.1 |  |  |
| 10 | 38.9 | 38.7 | 2.99, m | 2.99, m |
| 11 | 74.1 | 74.8 | 4.17, m | 4.20, m |
| 12 | 49.7 | 50.3 | 2.18, m | 2.18, m |
| 13 | 210.4 | 211.8 |  |  |
| 14 | 28.0 | 28.5 | 2.04, s | 2.04, s |
| 15 | 62.8 | 63.4 | 4.46, s | 4.45, brs |
| 16 | 19.4 | 19.7 | 1.21, d (6.8) | 1.21, d (6.8) |
| 17 | 8.5 | 9.1 | 0.81, d (6.9) | 0.80, d (7.0) |
| ***^a^*** *J Nat Prod*. 2020, 83, 111-117. | | | | |

| Table S7. ^1^H and ^13^C NMR data for 5 in DMSO-*d*_6_ | | | | | |
| --- | --- | --- | --- | --- | --- |
| No. | *δ*_C_ (150 MHz) | *δ*_H,_ mult.  (600 MHz. *J* in Hz) | COSY | HMBC | NOESY |
| 1 | 115.7 | 4.97, dd (10.2, 1.9)  5.10, dd (17.1, 2.0) | 2 |  |  |
| 2 | 137.1 | 6.29, m | 1 |  |  |
| 3 | 131.7 | 6.06, dd (15.2, 10.5) | 4 | 1 |  |
| 4 | 132.2 | 5.66, dt (14.5, 6.9) | 3, 5 | 2 |  |
| 5 | 34.9 | 2.88, dt (14.8, 7.1) | 4 |  |  |
| 6 | 134.3 |  |  |  |  |
| 7 | 126.2 | 5.83, d (10.8) | 8 | 5 |  |
| 8 | 125.8 | 6.24, m | 7, 9 |  |  |
| 9 | 135.4 | 5.48, dd (15.1, 8.8) | 8, 10 | 7, 19 |  |
| 10 | 40.9 | 2.18, p (7.1) | 9, 19 |  |  |
| 11 | 74.5 | 3.45, m |  |  | 11-OH、15-OH |
| 12 | 40.8 | 2.55, qd (6.7, 3.9) | 20 |  |  |
| 13 | 165.4 |  |  |  |  |
| 14 | 99.2 | 5.96, s |  | 13, 16 |  |
| 15 | 165.2 |  |  |  |  |
| 16 | 96.7 |  |  |  |  |
| 17 | 164.9 |  |  |  |  |
| 18 | 23.5 | 1.70, d (1.3) |  | 5, 6, 7 |  |
| 19 | 17.1 | 0.99, d (6.6) | 10 | 11 |  |
| 20 | 10.8 | 1.05, d (6.9) | 12 | 11, 13 |  |
| 21 | 8.4 | 1.73, s |  | 15, 16, 17 |  |
| 15-OH |  | 11.06, s |  |  |  |
| 11-OH |  | 4.84, d (6.7) |  |  |  |

| **Table S8.** ^1^H and ^13^C NMR data for **6** in DMSO-*d*_6_ | | | | | |
| --- | --- | --- | --- | --- | --- |
| No. | *δ*_C_ (150 MHz) | *δ*_H,_ mult.  (600 MHz. *J* in Hz) | COSY | HMBC | NOESY |
| 1 | 64.5 | 3.60, dd (11.8, 7.5)，  3.49, dd (11.8, 3.8) | 2 | 1 |  |
| 2 | 80.3 | 4.90, td (7.1, 3.6) | 3, 1 |  | 1 |
| 3 | 127.0 | 5.47, ddd (15.8, 6.9, 2.3) | 2 |  |  |
| 4 | 133.9 | 5.76, ddd (15.8, 8.8, 3.4) | 5 | 18 |  |
| 5 | 35.5 | 3.06, dd (16.4, 8.8)  2.54, t (3.0) | 4 | 3, 18 |  |
| 6 | 134.8 |  |  |  |  |
| 7 | 126.4 | 5.81, d (11.1) |  | 5, 9, 18 |  |
| 8 | 126.3 | 6.00, dd (15.1, 11.1) | 9 | 10 |  |
| 9 | 135.6 | 5.40, dd (15.1, 9.5) | 10, 8 |  |  |
| 10 | 41.8 | 2.00, m | 8, 19 |  |  |
| 11 | 76.7 | 3.40, s |  |  |  |
| 12 | 44.2 | 2.64, p (7.2) | 20 | 13, 11 |  |
| 13 | 166.0 |  |  |  |  |
| 14 | 96.5 | 6.10, s |  | 12, 13, 16 |  |
| 15 | 165.1 |  |  |  |  |
| 16 | 100.7 |  |  |  |  |
| 17 | 164.6 |  |  |  |  |
| 18 | 25.8 | 1.78, s |  | 5, 6, 7 |  |
| 19 | 16.4 | 0.88, d (6.7) | 10 | 9, 10, 11 |  |
| 20 | 17.2 | 1.13, d (7.2), | 12 | 13, 11 |  |
| 21 | 8.8 | 1.81, s |  | 15, 17 |  |

| Table S9. ^1^H and ^13^C NMR data for 7 in DMSO-*d*_6_ | | | | | |
| --- | --- | --- | --- | --- | --- |
| No. | *δ*_C_ (150 MHz) | *δ*_H,_ mult.  (600 MHz. *J* in Hz) | COSY | HMBC | NOESY |
| 1 | 60.9 | 3.38 | 2 |  |  |
| 2 | 35.9 | 2.11, q (6.8) | 1, 3 |  |  |
| 3 | 135.1 | 5.44 | 2 |  |  |
| 4 | 128.4 | 5.35, dt (15.2, 6.5) | 5 |  |  |
| 5 | 35.2 | 2.79, q (6.7, 5.3) | 4 |  |  |
| 6 | 135.0 |  |  |  |  |
| 7 | 125.7 | 5.78, d (10.8) | 8 | 5, 6 |  |
| 8 | 125.7 | 6.25, dd (15.1, 10.8) | 7, 9 |  |  |
| 9 | 128.3 | 5.44 | 8, 10 |  |  |
| 10 | 40.8 | 2.18 | 9, 19, 11 |  |  |
| 11 | 74.5 | 3.45 | 10 |  |  |
| 12 | 40.8 | 2.54 | 20 | 14 |  |
| 13 | 165.7 |  |  |  |  |
| 14 | 99.6 | 5.94, s |  | 16, 17 |  |
| 15 | 165.2 |  |  |  |  |
| 16 | 96.4 |  |  |  |  |
| 17 | 165.1 |  |  |  |  |
| 18 | 23.3 | 1.68, d (1.3) |  | 5, 7 |  |
| 19 | 17.0 | 0.98, d (6.6) | 10 | 9, 11 |  |
| 20 | 10.9 | 1.05, d (6.9) | 12 | 11 |  |
| 21 | 8.5 | 1.72, s |  | 15. 17 |  |

| **Table S10.** ^1^H and ^13^C NMR data for **8** in DMSO-*d*_6_ | | | | |
| --- | --- | --- | --- | --- |
| No. | *δ*_C_ (150 MHz) | *δ*_H,_ mult.  (600 MHz. *J* in Hz) | COSY | HMBC |
| 1 | 66.2 | 3.23-3.15, m |  | 2, 3 |
| 2 | 72.0 | 3.85, q (5.8) |  |  |
| 3 | 132.5 | 5.44-5.38, m |  |  |
| 4 | 127.1 | 5.47, dt (15.7, 6.4) |  | 5 |
| 5 | 34.8 | 2.76, dt (10.9, 5.6) | 4 | 18 |
| 6 | 134.8 |  |  |  |
| 7 | 125.8 | 5.75, d (10.8) |  | 5, 6, 8, 18 |
| 8 | 125.7 | 6.22, dd (15.1, 10.8) | 7 |  |
| 9 | 135.2 | 5.44-5.38, m | 8 |  |
| 10 | 40.9 | 2.13, m (7.0) | 9, 11, 19 |  |
| 11 | 74.5 | 3.40, dd (7.8, 4.3) |  |  |
| 12 | 40.8 | 2.50, q (1.9) |  |  |
| 13 | 165.3 |  |  |  |
| 14 | 99.4 | 5.91, s |  | 12, 16, 17 |
| 15 | 165.2 |  |  |  |
| 16 | 96.5 |  |  |  |
| 17 | 165.2 |  |  |  |
| 18 | 23.3 | 1.64, s |  | 5, 6, 7 |
| 19 | 17.0 | 0.94, d (6.6) | 10 | 9, 11, 12 |
| 20 | 10.8 | 1.01, d (6.9) | 12 | 11, 13 |
| 21 | 8.4 | 1.68, s |  | 15, 17, 16 |

**Fig. S1. ^1^H-^1^H COSY and key HMBC correlations of 1 and 2**

**
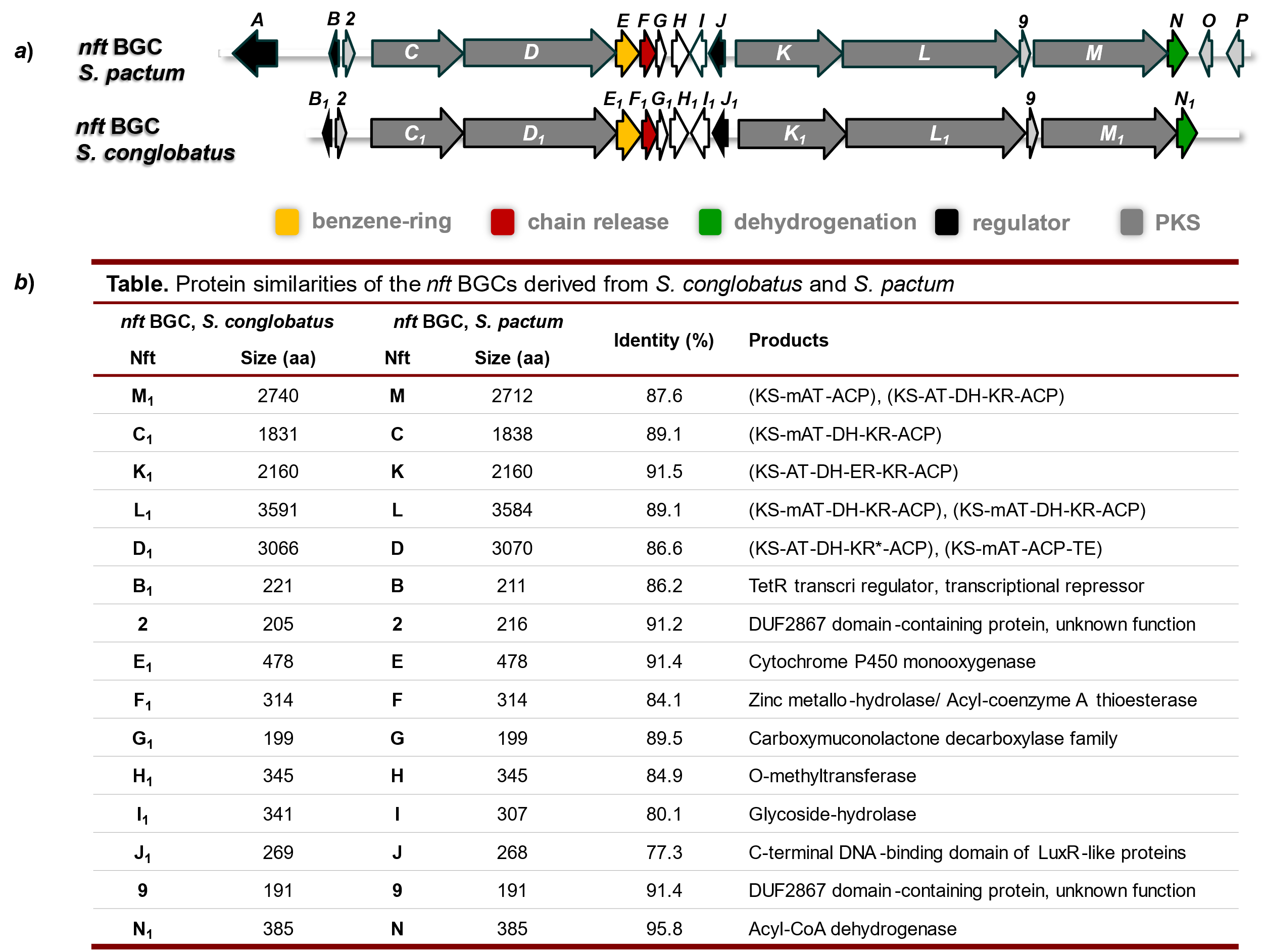
**

**Fig. S2.** **Comparison of the *nft* BGCs derived from *S. conglobatus* and *S. pactum*.** Gene organization of the *nft* BGCs derived from *S. conglobatus* and *S. pactum* (***a***) and the similarities of proteins encoded by the two BGCs (***b***).


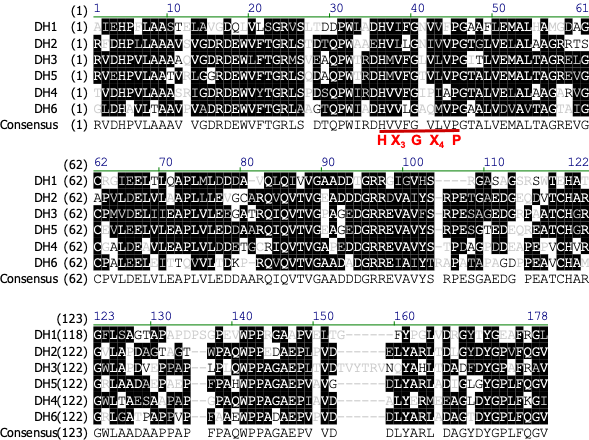


**Fig. S3. Protein sequence alignment of the DH domains of the nft BGC from *S. conglobatus*.** The conservative HX_3_GX_4_P motif in DHs is labeled in red.


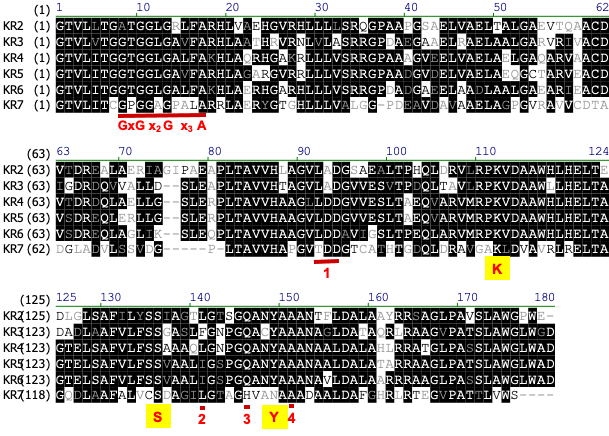


**Fig. S4.** **Protein sequence alignment of the KR domains of the *nft* BGC from *S. conglobatus*.**

The motifs diagnostic of established KR types: A1 (1 no LDD; 2 W, 3 no H); A2 (1 no LDD; 2 W, 3 H); B1 (1 LDD; 4 no P); B2 (1 LDD; 4 P) (*Chem Biol* 2007, 14:898-908).

The KR4, KR5 and KR6 should belong to B1 type; KR7 lacks the Y conservative active site; The KR2 and KR3 lack the conserved sequence motifs of B1 type KRs;

**
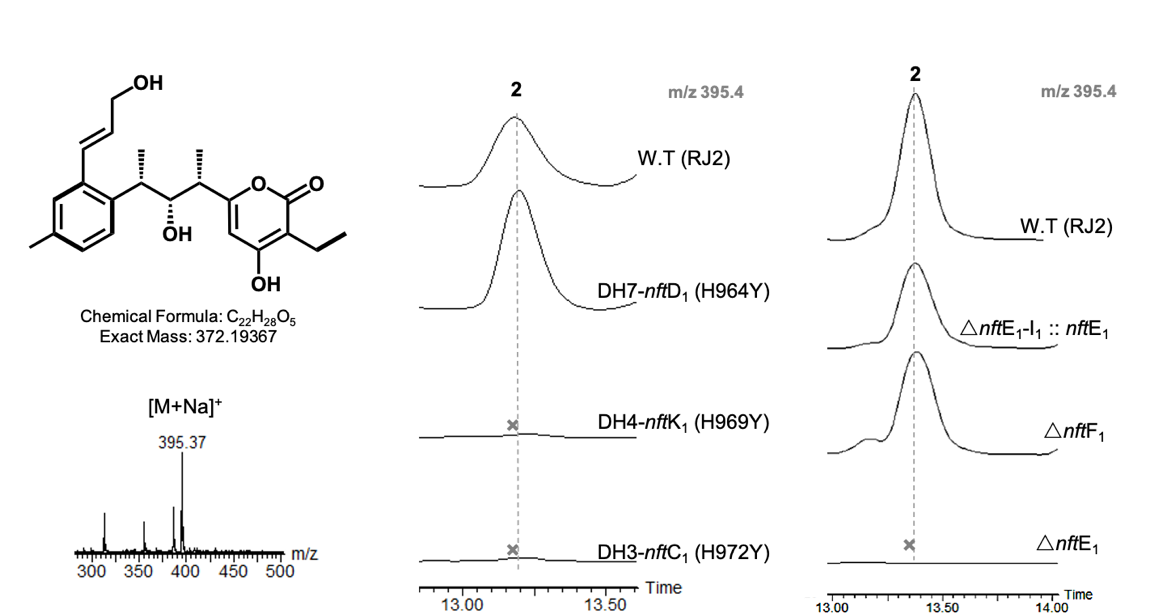
**

**Fig. S5. HPLC–MS analysis of the production of compound 2 in mutant strains.** The crosses mean that the target compounds were not detected.


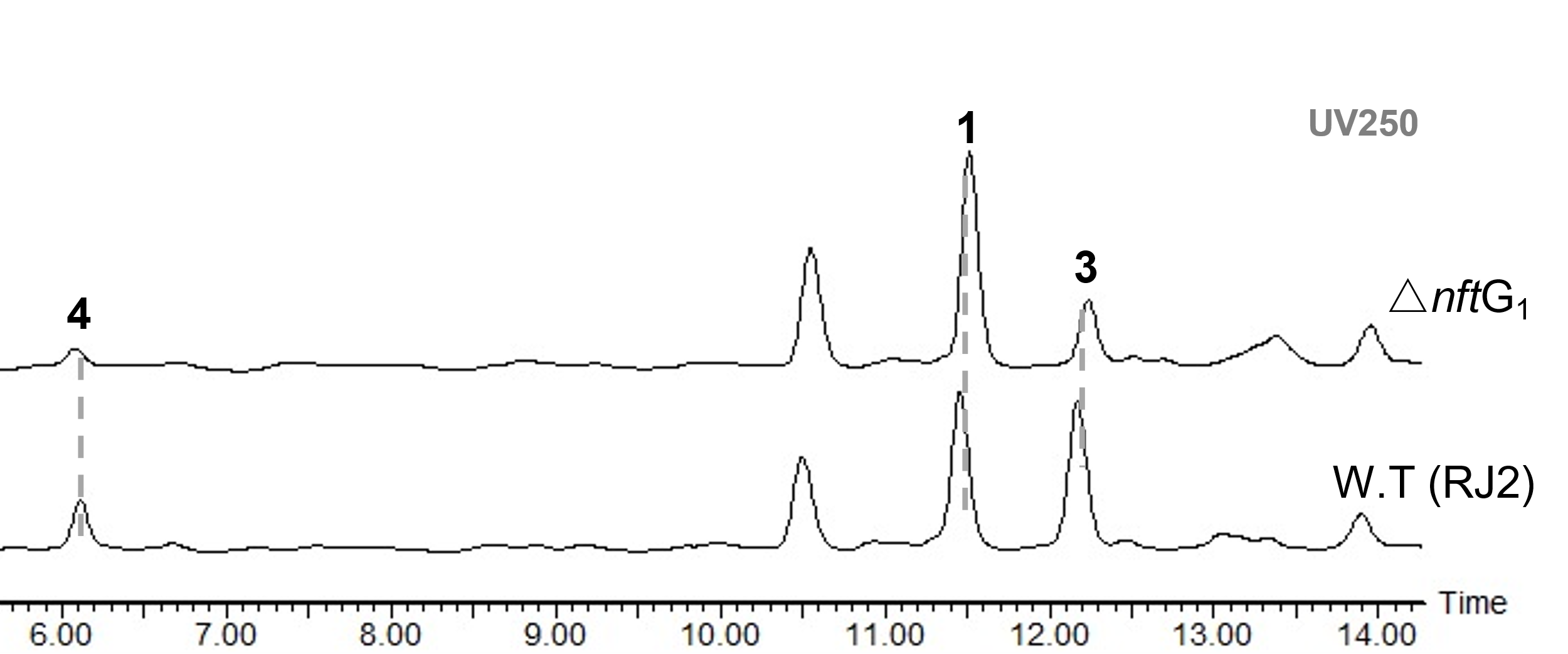


**Fig. S6. HPLC–MS analysis of the culture extract of Δ*nft*G_1_ mutant strain.**


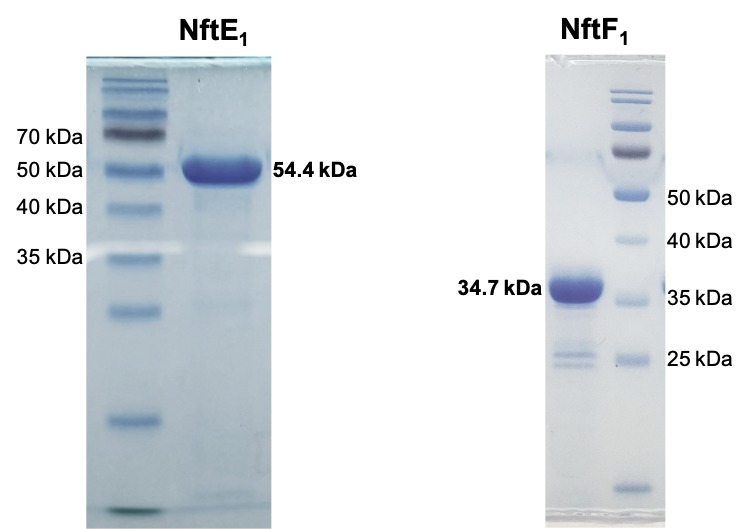


**Fig. S7****. SDS-PAGE analysis of the recombinant proteins NftE_1_ and NftF_1_.**

**
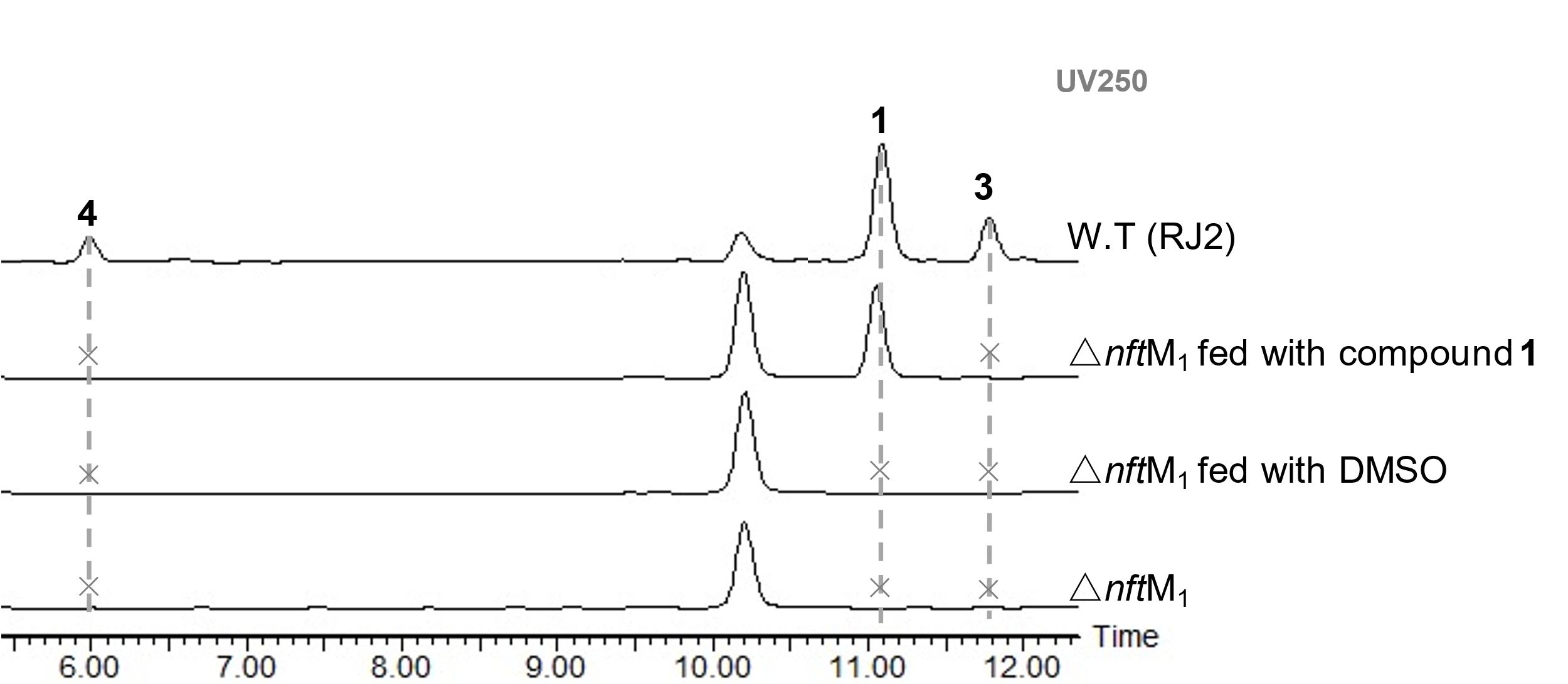
**

**Fig. S8. HPLC analysis of the culture extract of Δ*nft*M_1_ mutant strain fed with compound 1 and DMSO.** The crosses mean that the target compounds were not detected.

**
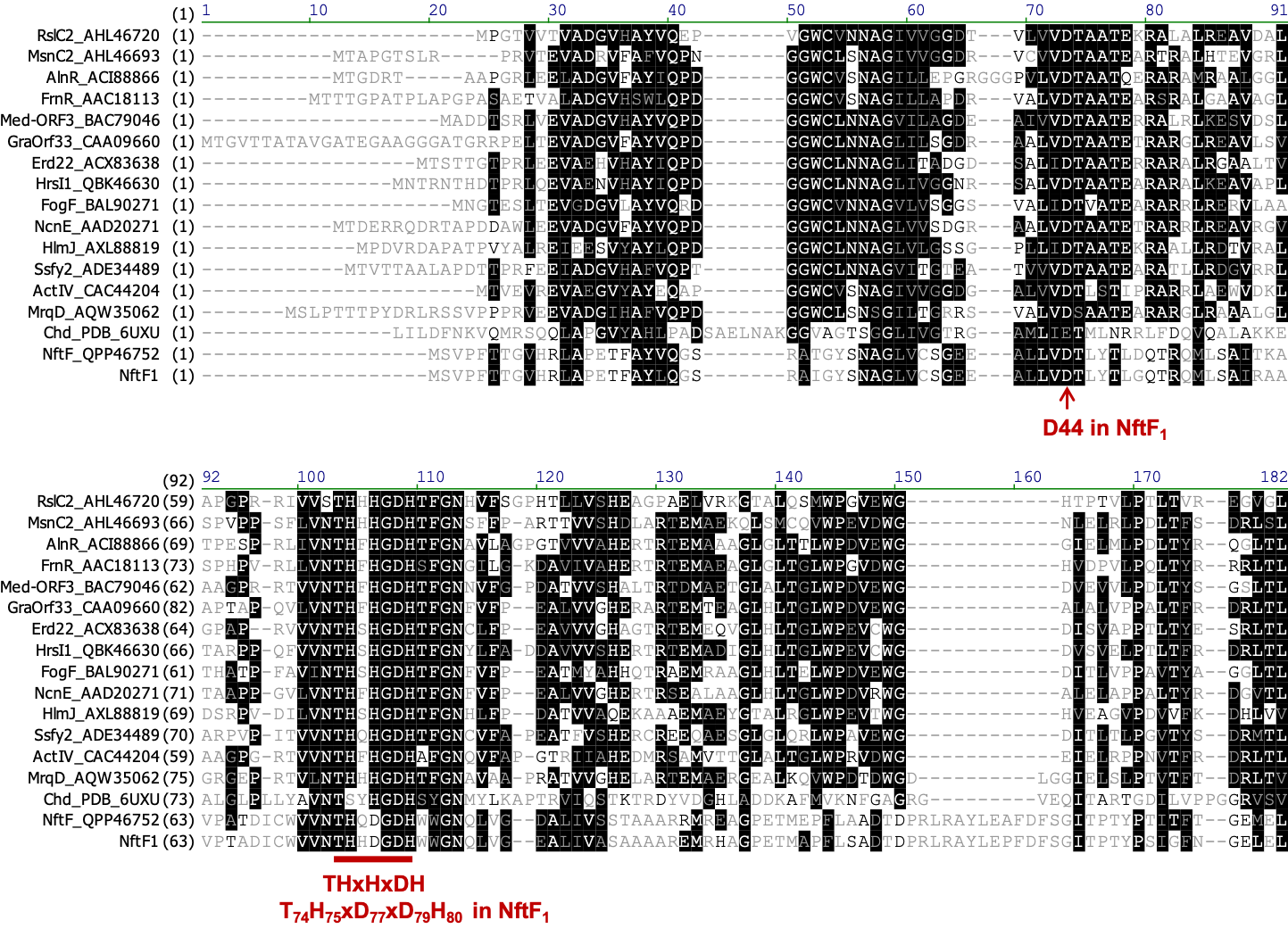

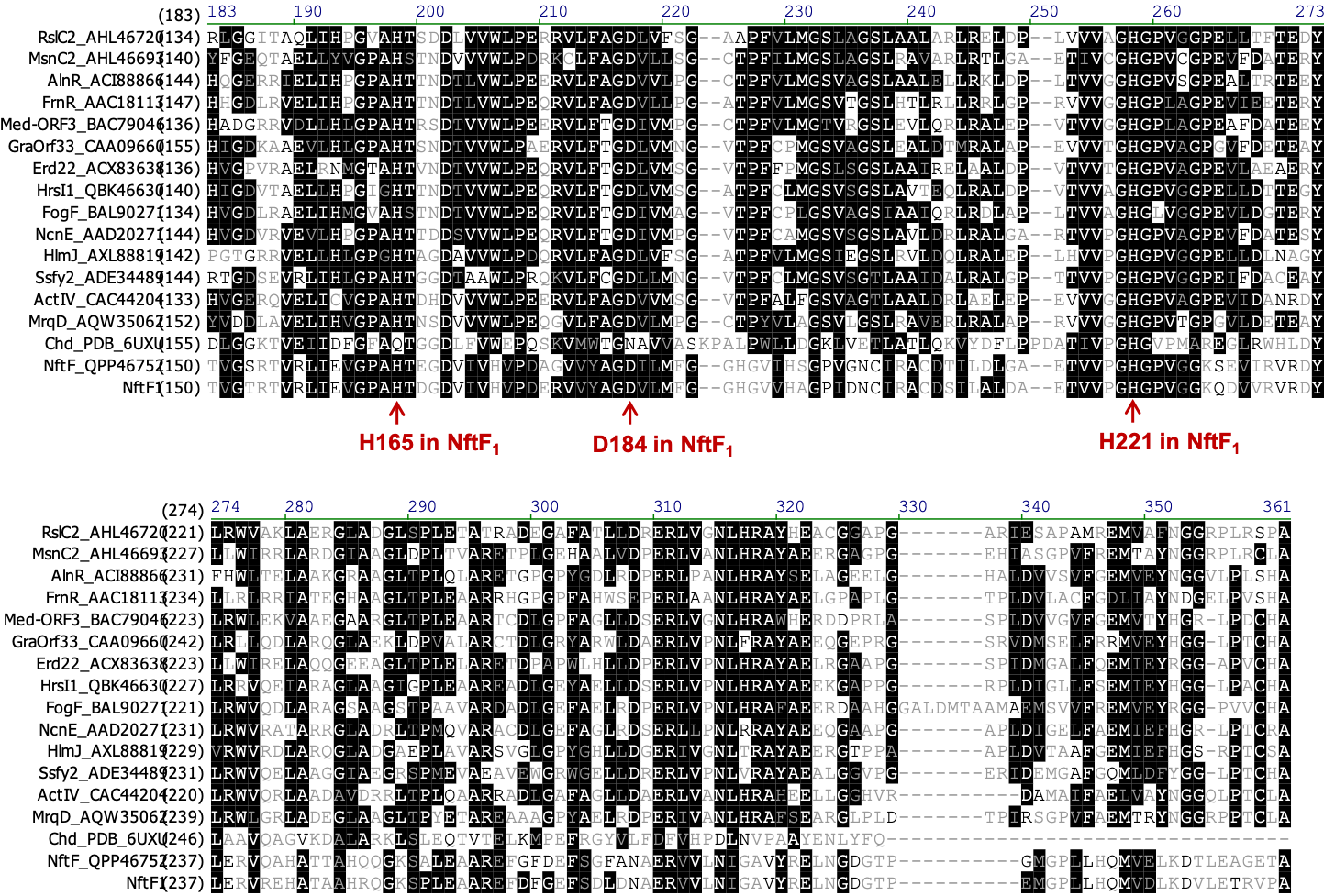
**

**Fig. S9. Protein sequence alignment of NftF_1_, Chd (PDB 6UXU) and its homologous proteins from the reported BGCs of type II PKS**.

The conservative THxDxDH motif in MBL hydrolase and the mutation sites in NftF_1_ are labeled.

**Fig. S10. NMR spectra of 1. (related to Table S3)**

1. ^1^H-NMR of **1**.


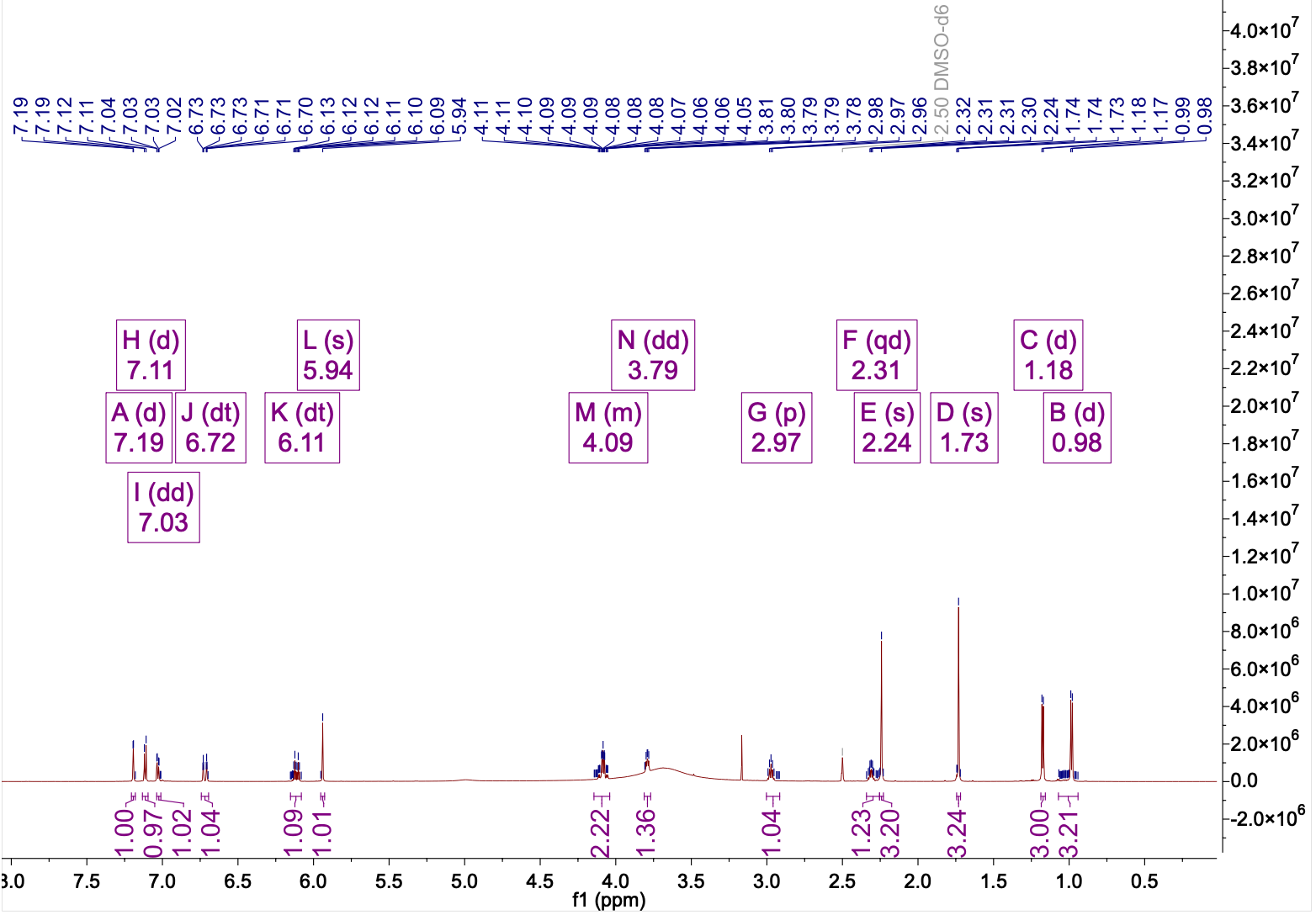


1. ^13^C-NMR of **1**


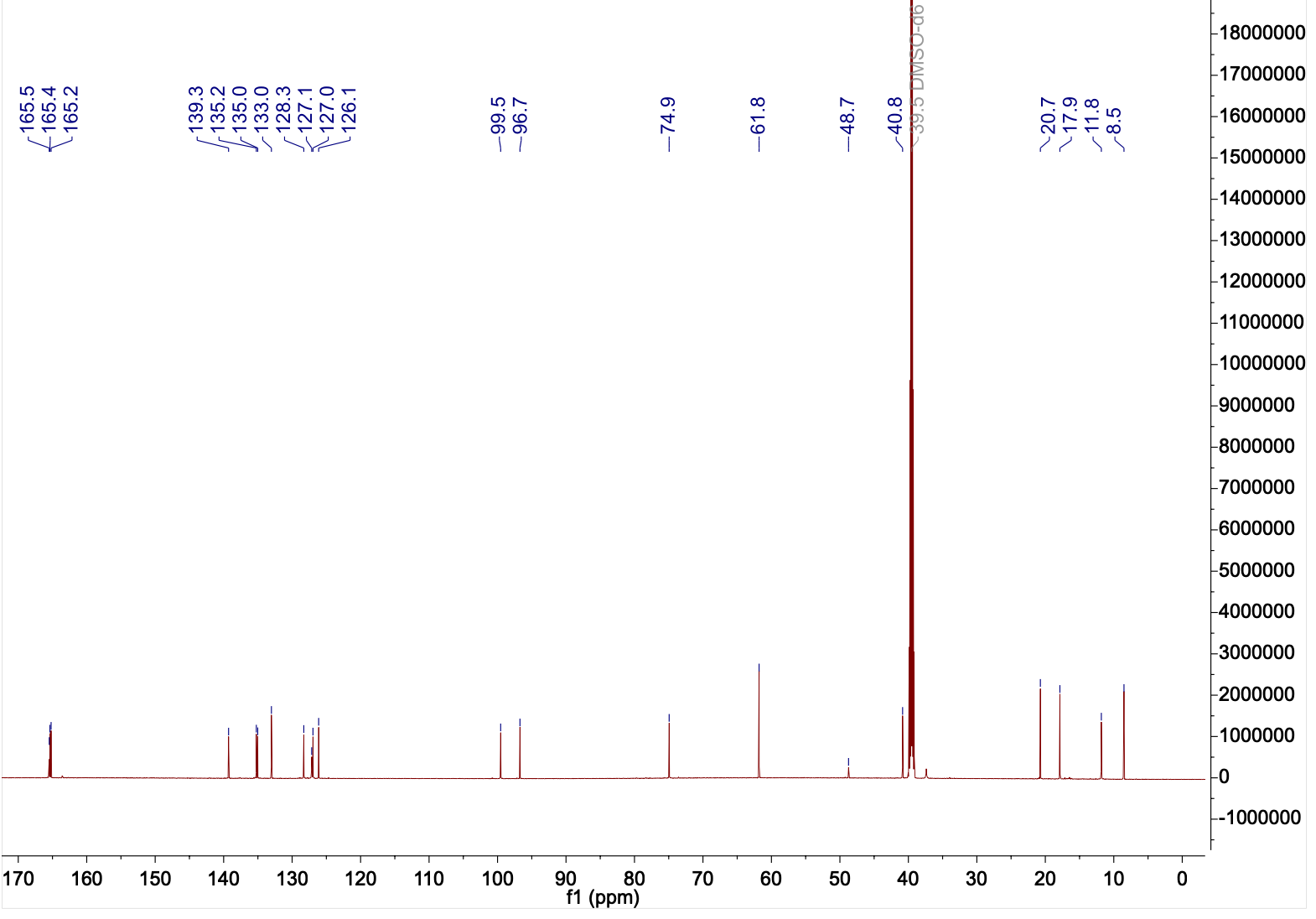


**Fig. S11.** NMR spectra of **2** (related to Table S4)

1. ^1^H-NMR of **2**


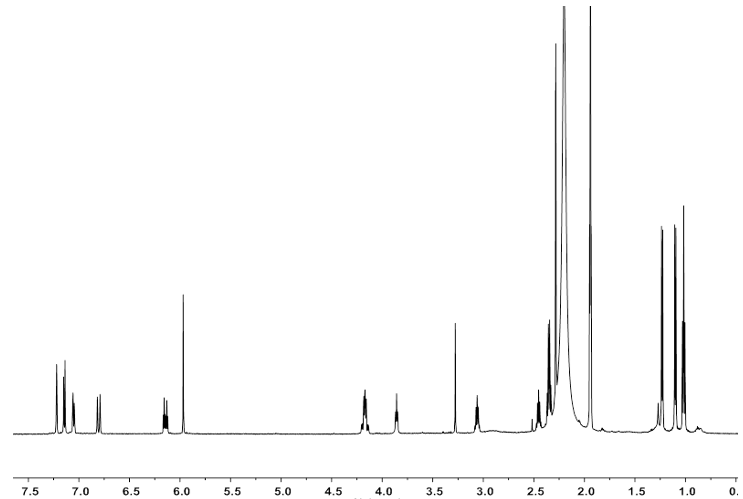


**b.** ^13^C-NMR of **2**


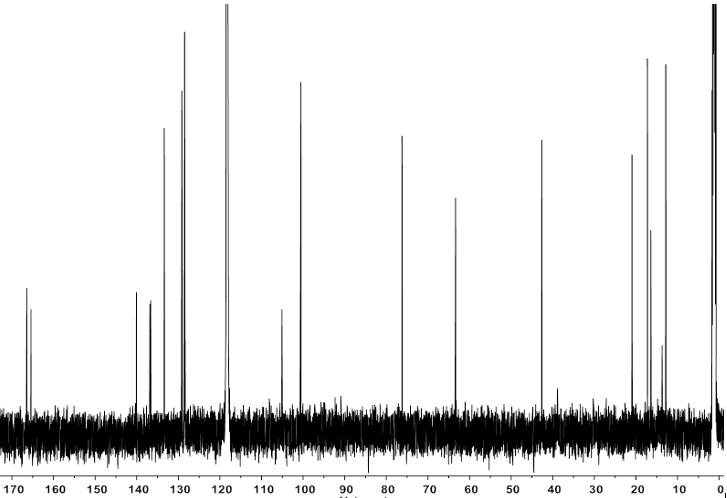


**Fig. S12.** NMR spectra of **3**. (related to Table S5)

**a.** ^1^H-NMR of **3**


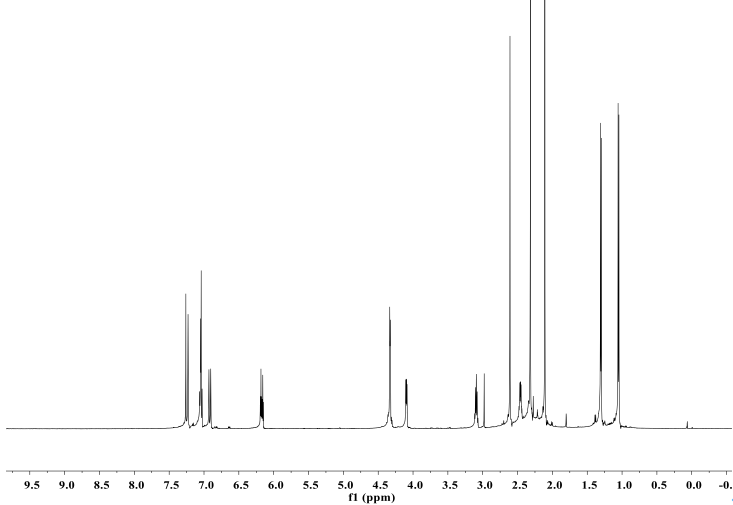


**b.** ^13^C-NMR of **3**


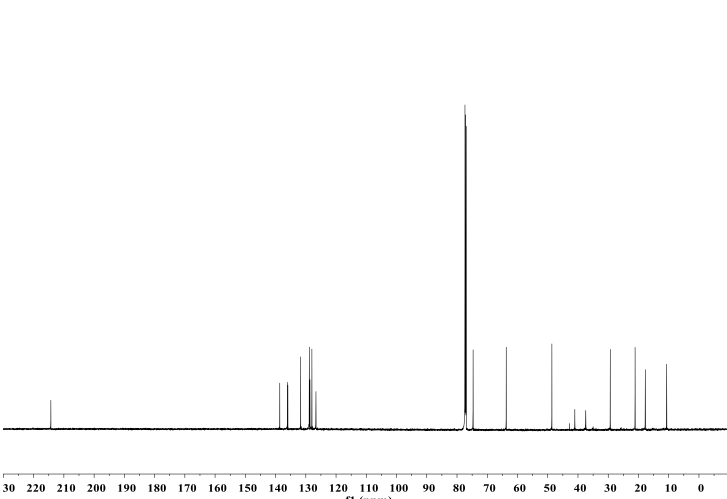


**Fig. S13.** NMR spectra of **4**. (related to Table S6)

1. ^1^H-NMR of **4**


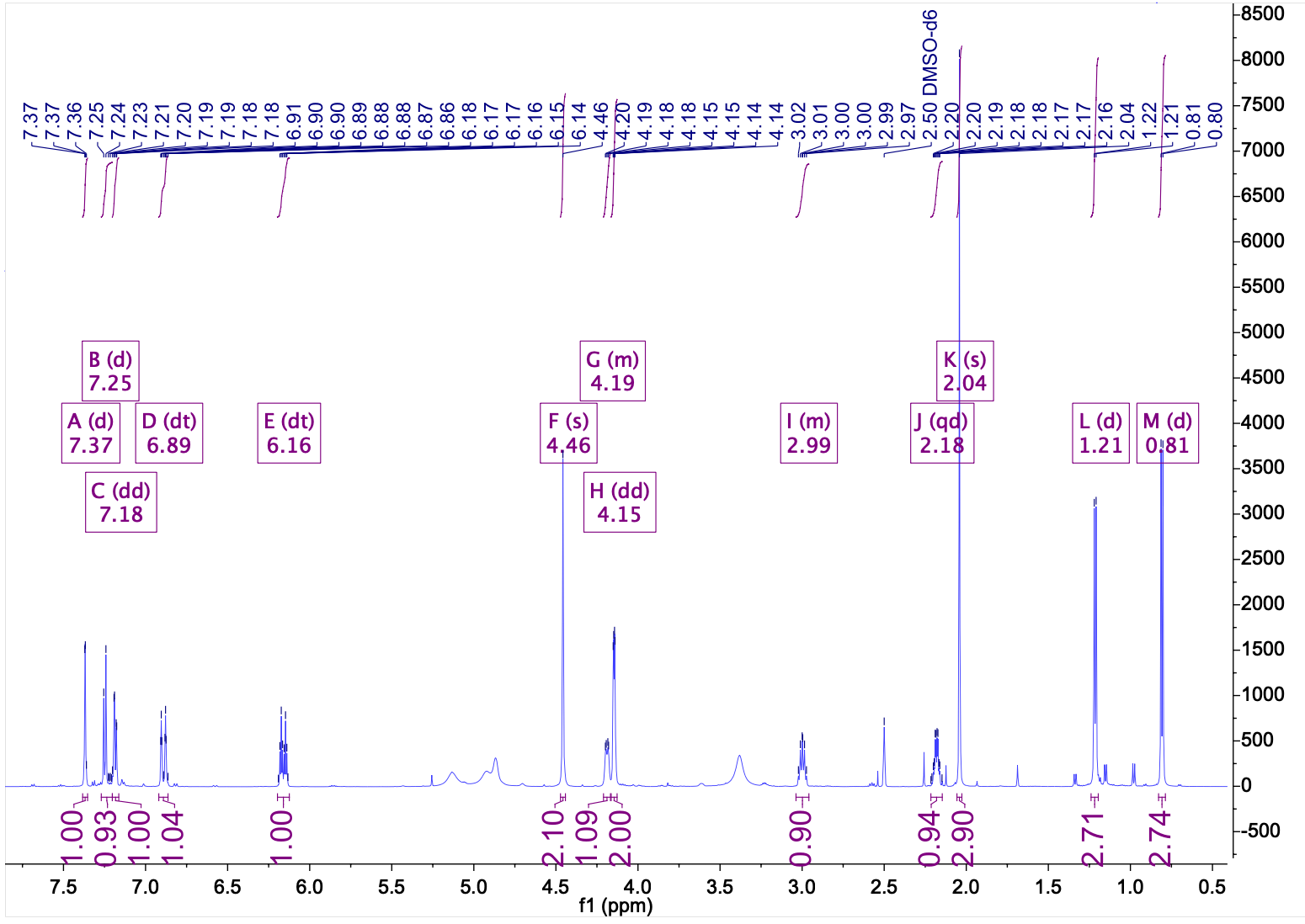


**b.** ^13^C-NMR of **4**


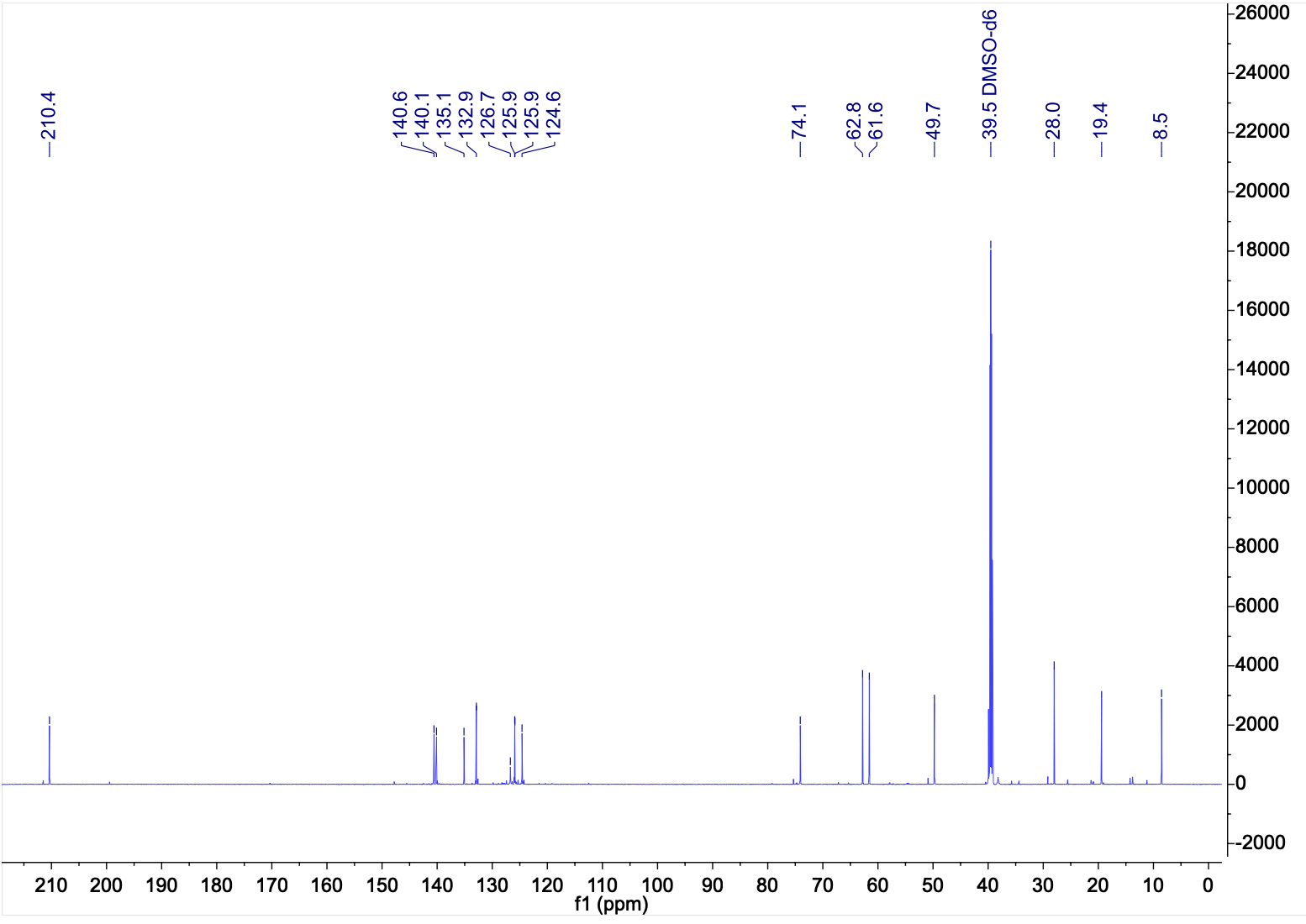


**Fig. S14.** NMR spectra of **5** (related to Table S7)

**a.** ^1^H-NMR of **5**


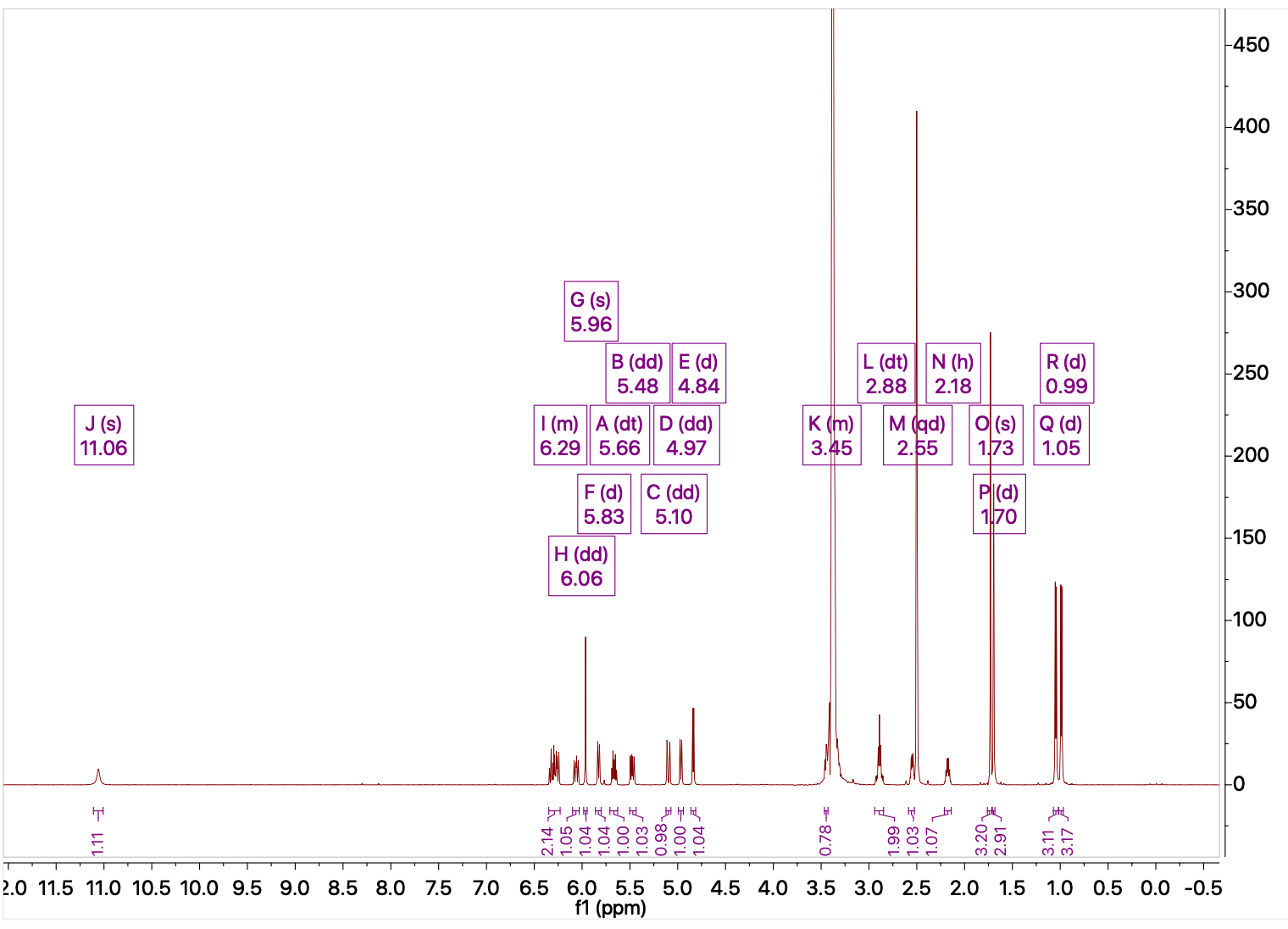


**b.** ^13^C-NMR of **5**


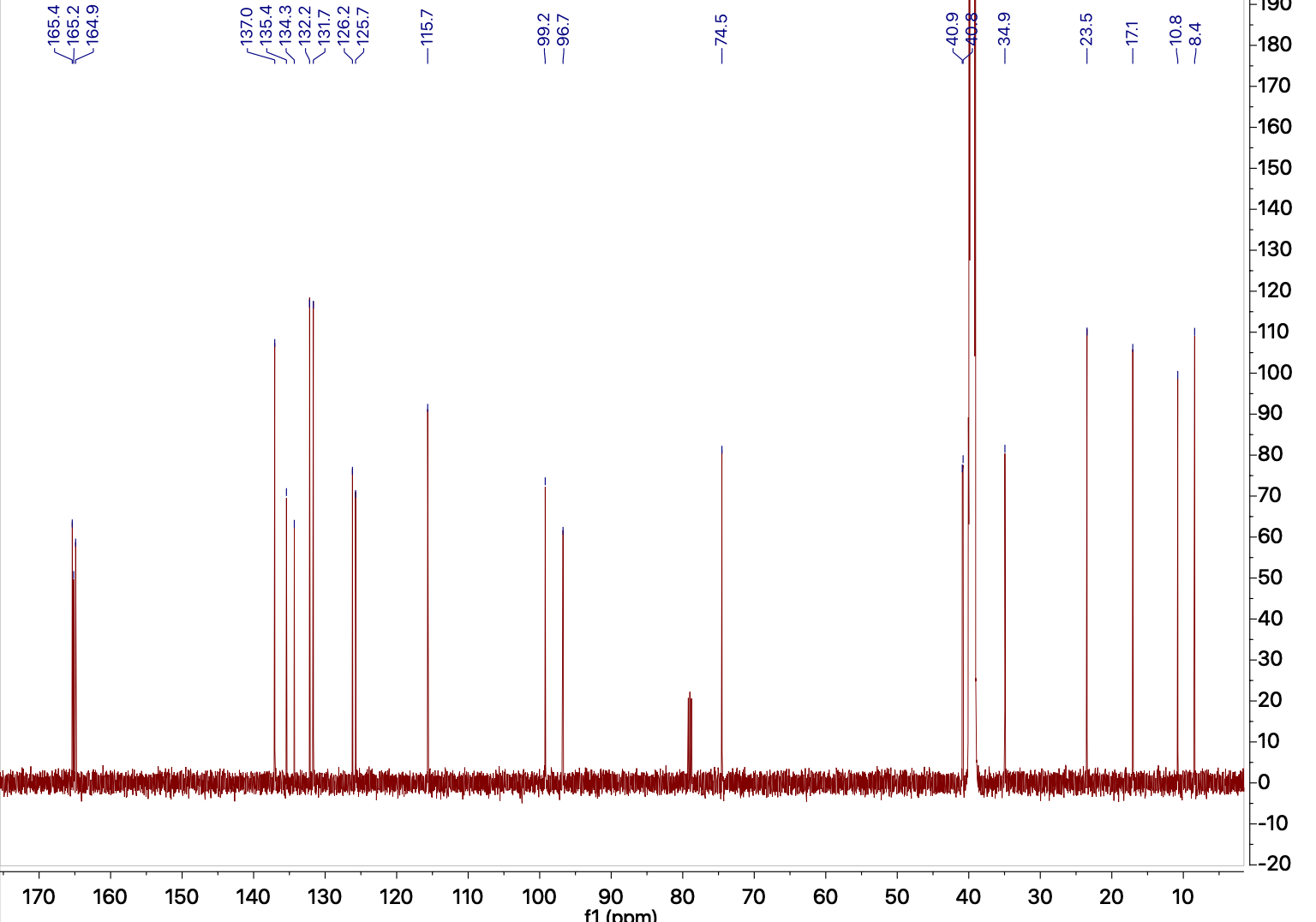


**c.** DEPT-NMR of **5**


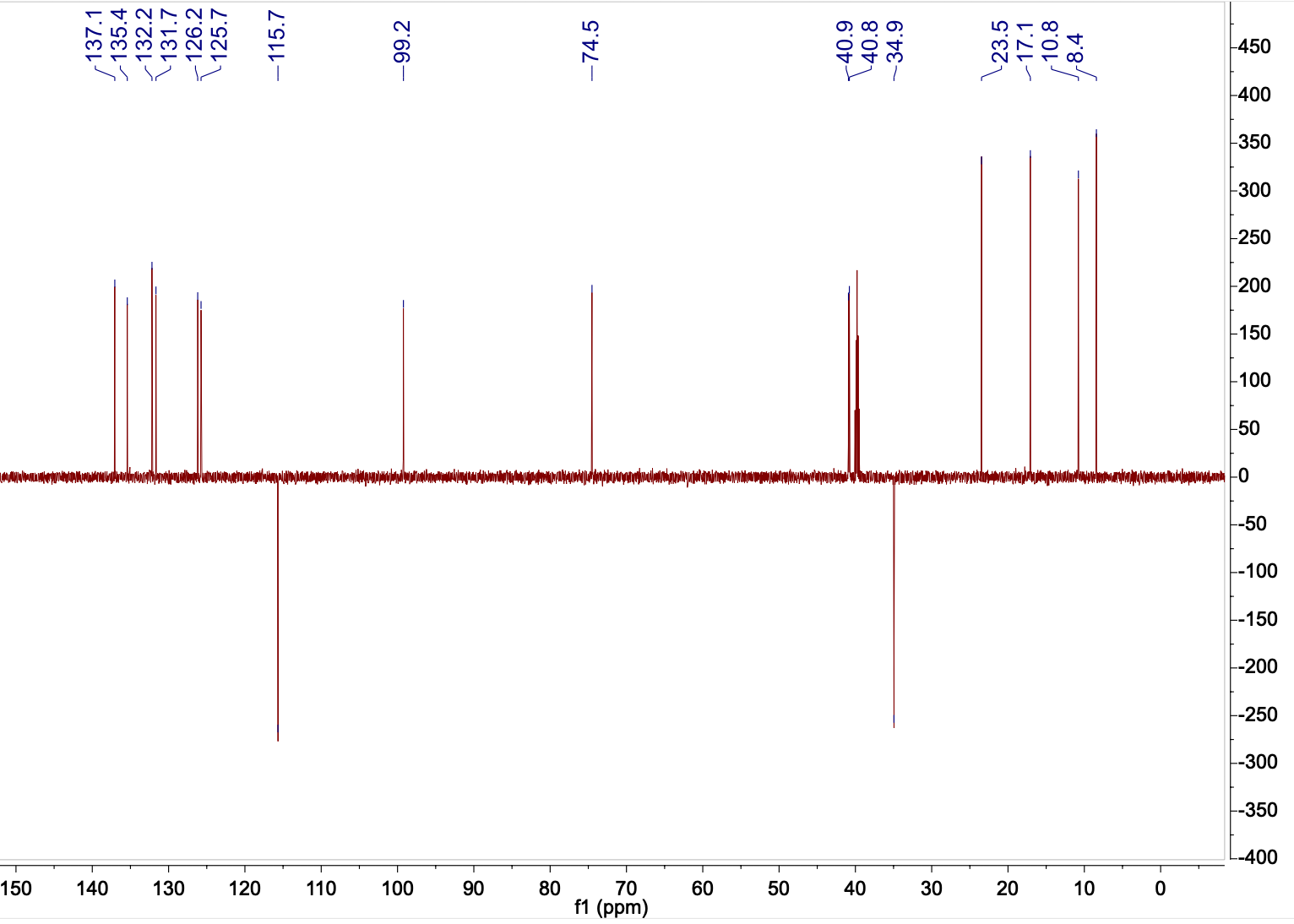


**d.** COSY of **5**


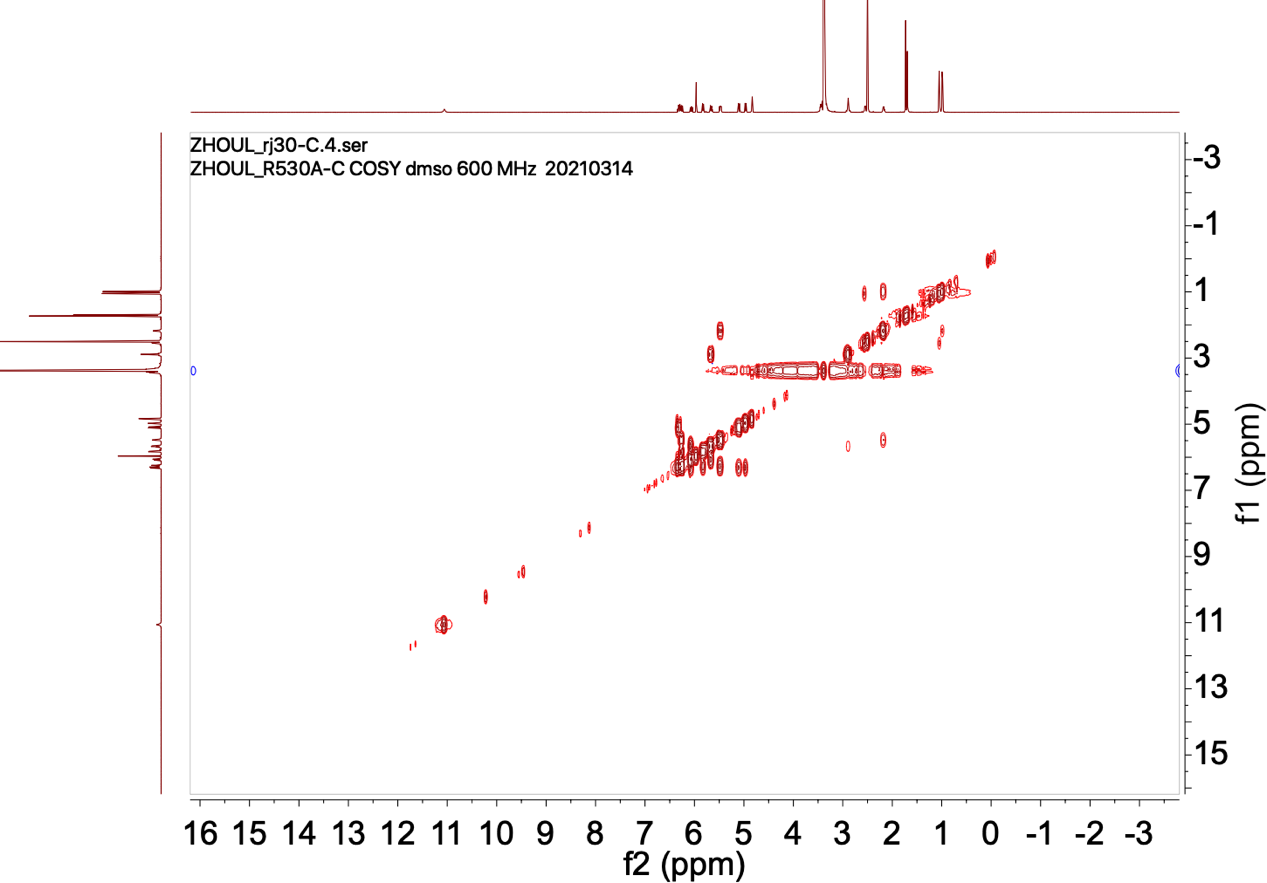


**e.** HSQC of **5**


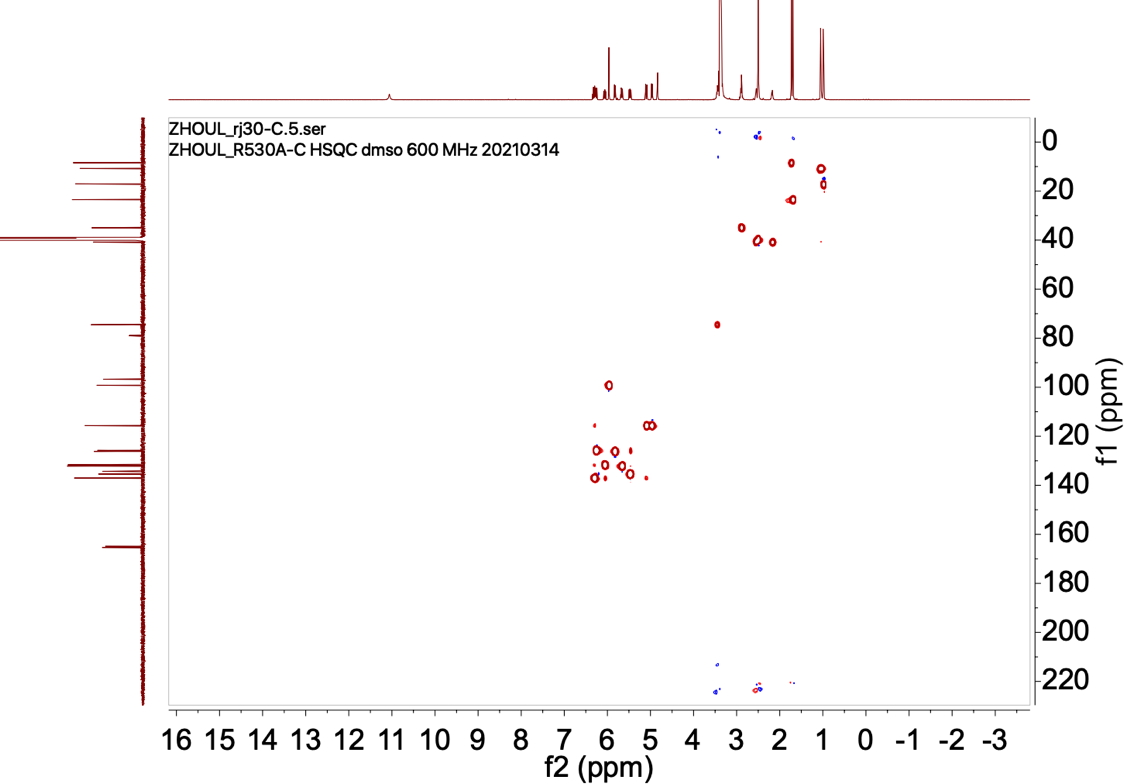


**f.** HMBC of **5**


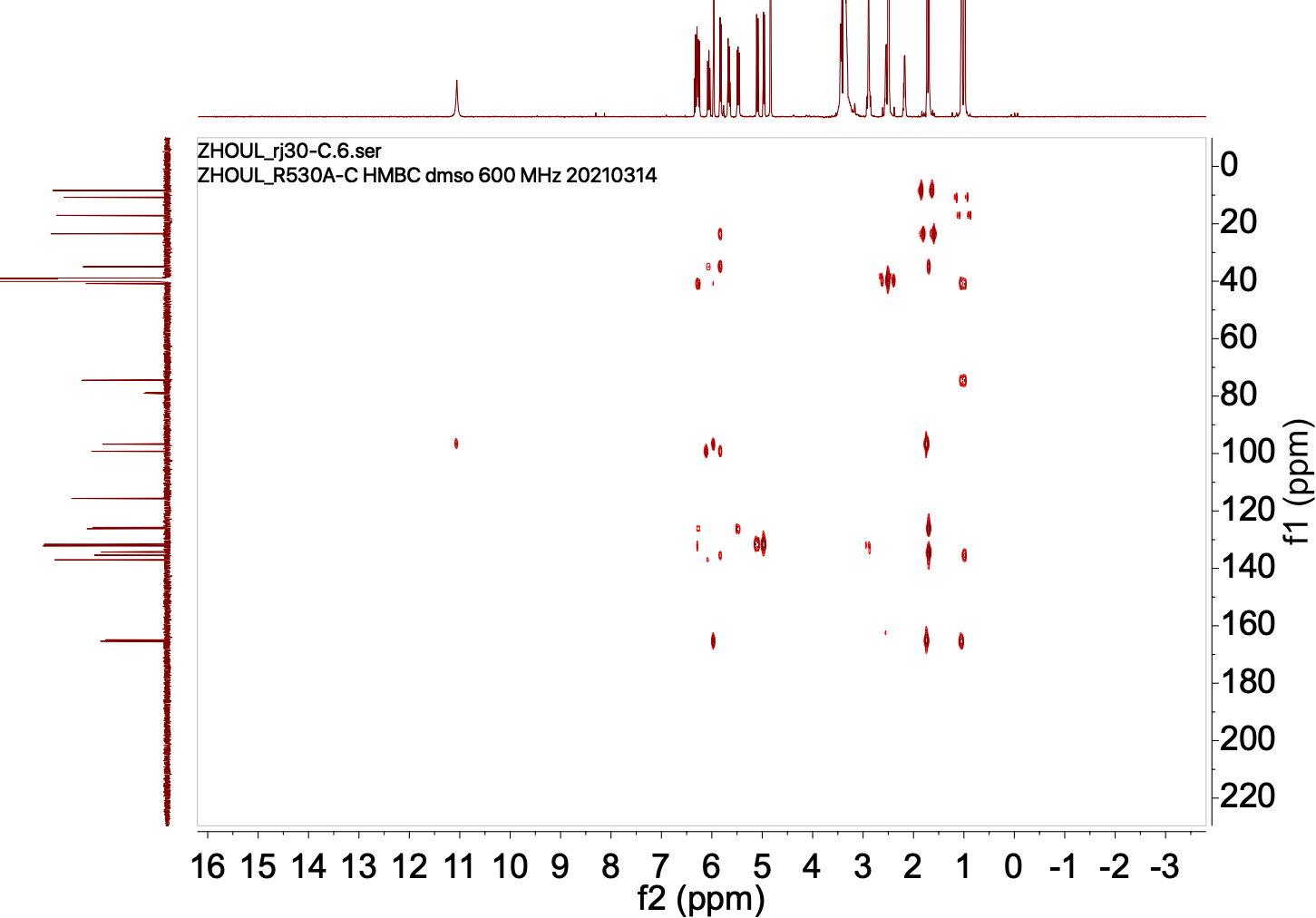


**g.** NOESY of **5**


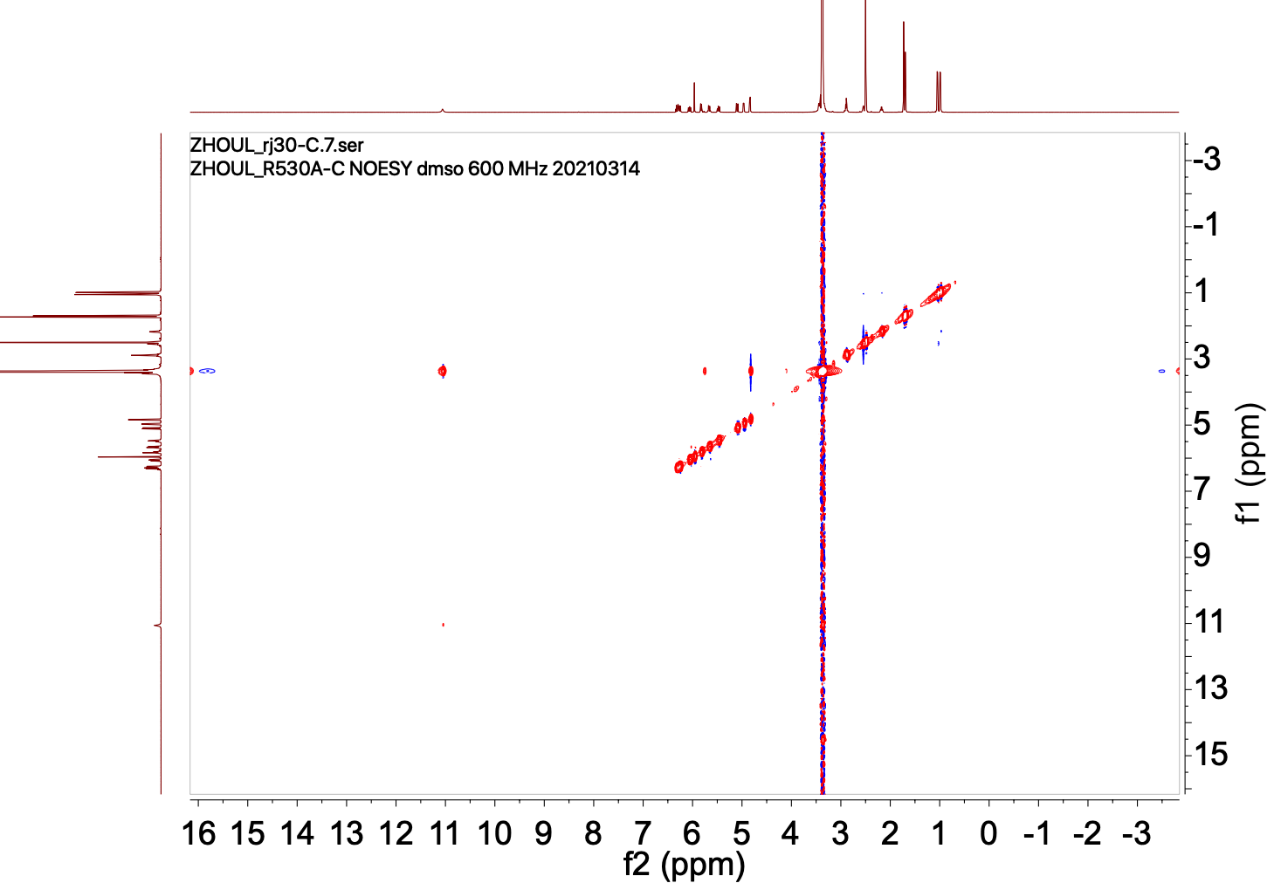


**Fig. S15.** NMR spectra of **6**. (related to Table S8)

**a.** ^1^H-NMR of **6**


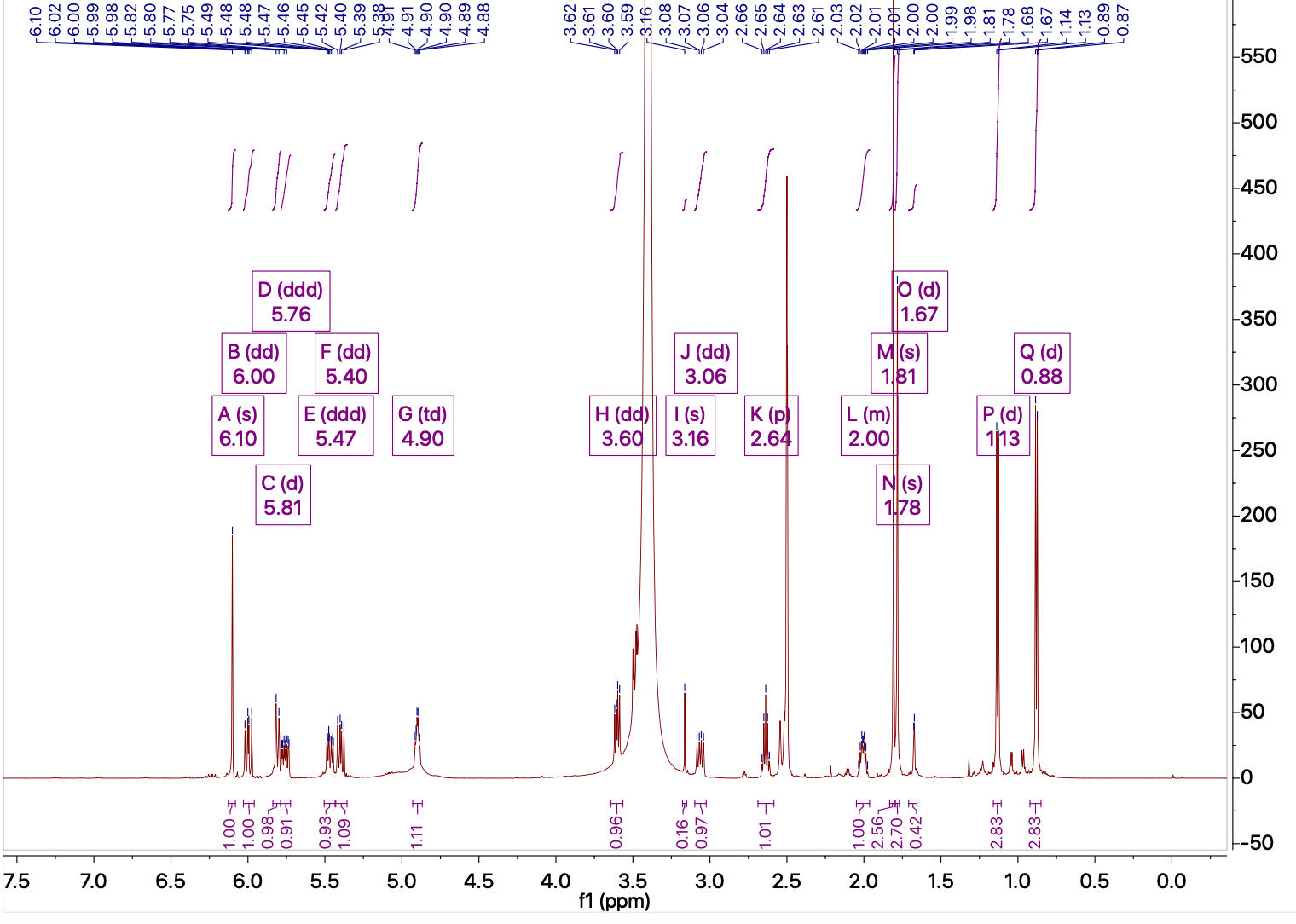


**b.** ^13^C-NMR of **6**


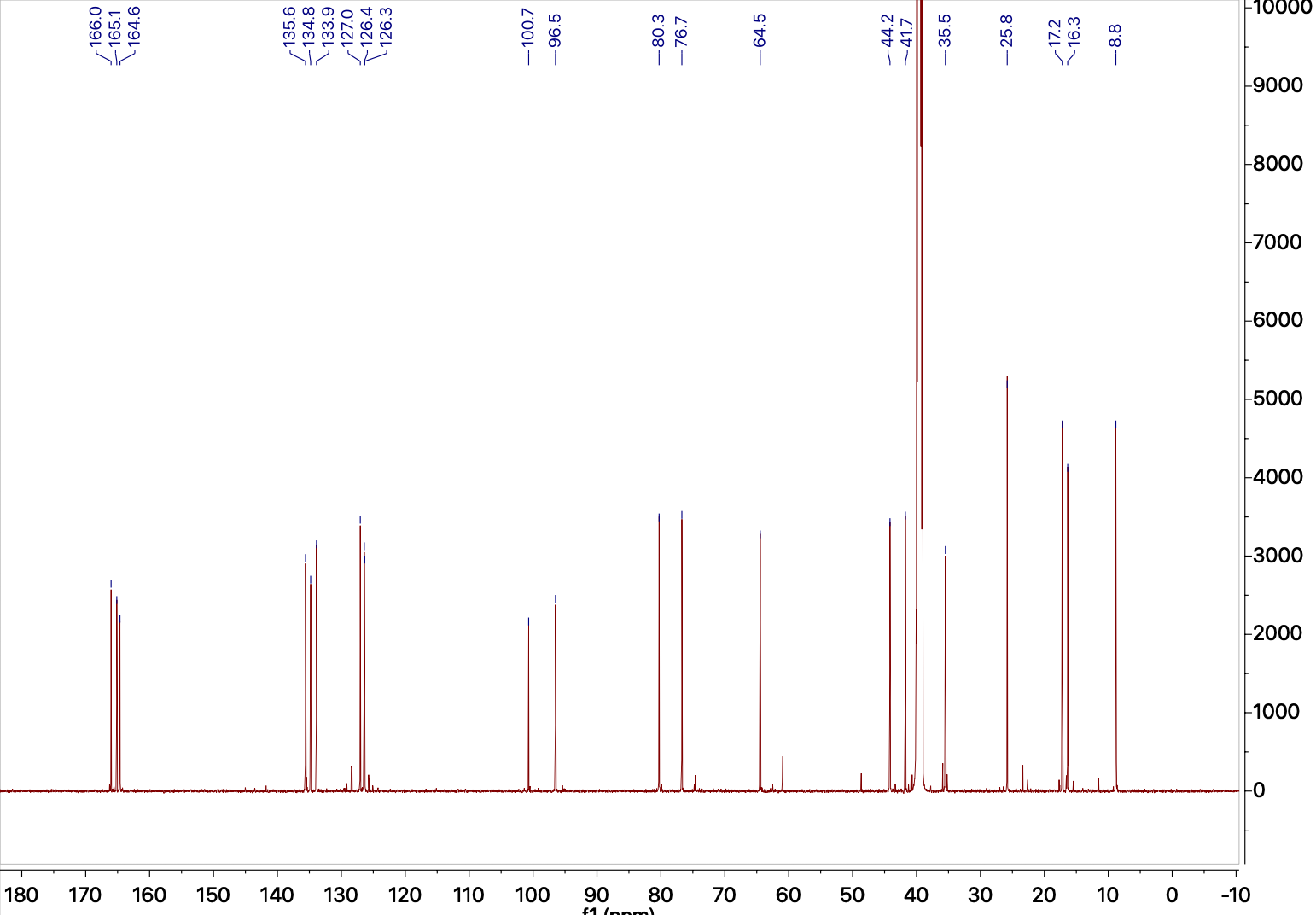


**c.** DEPT-NMR of **6**


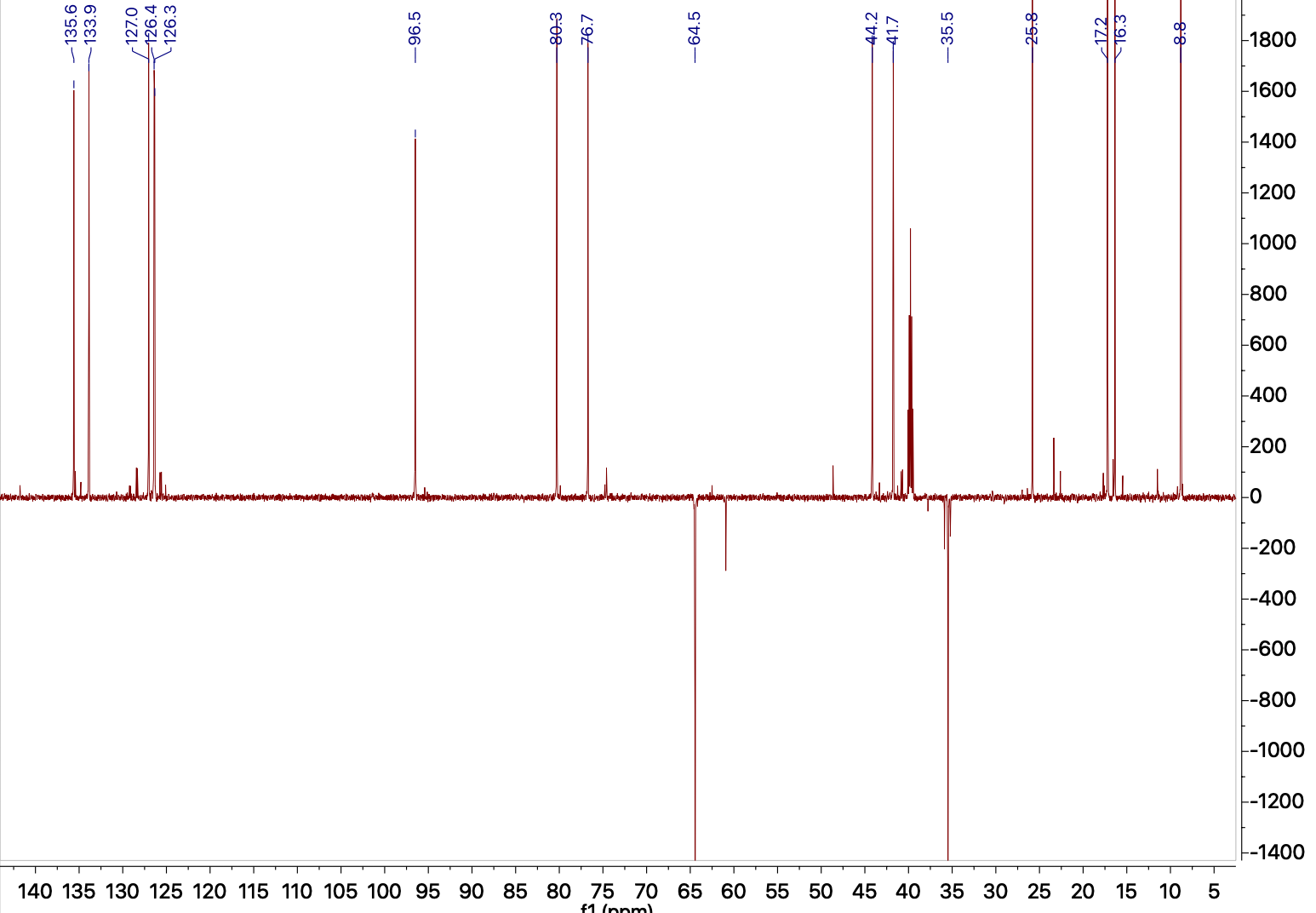


**d.** COSY of **6**


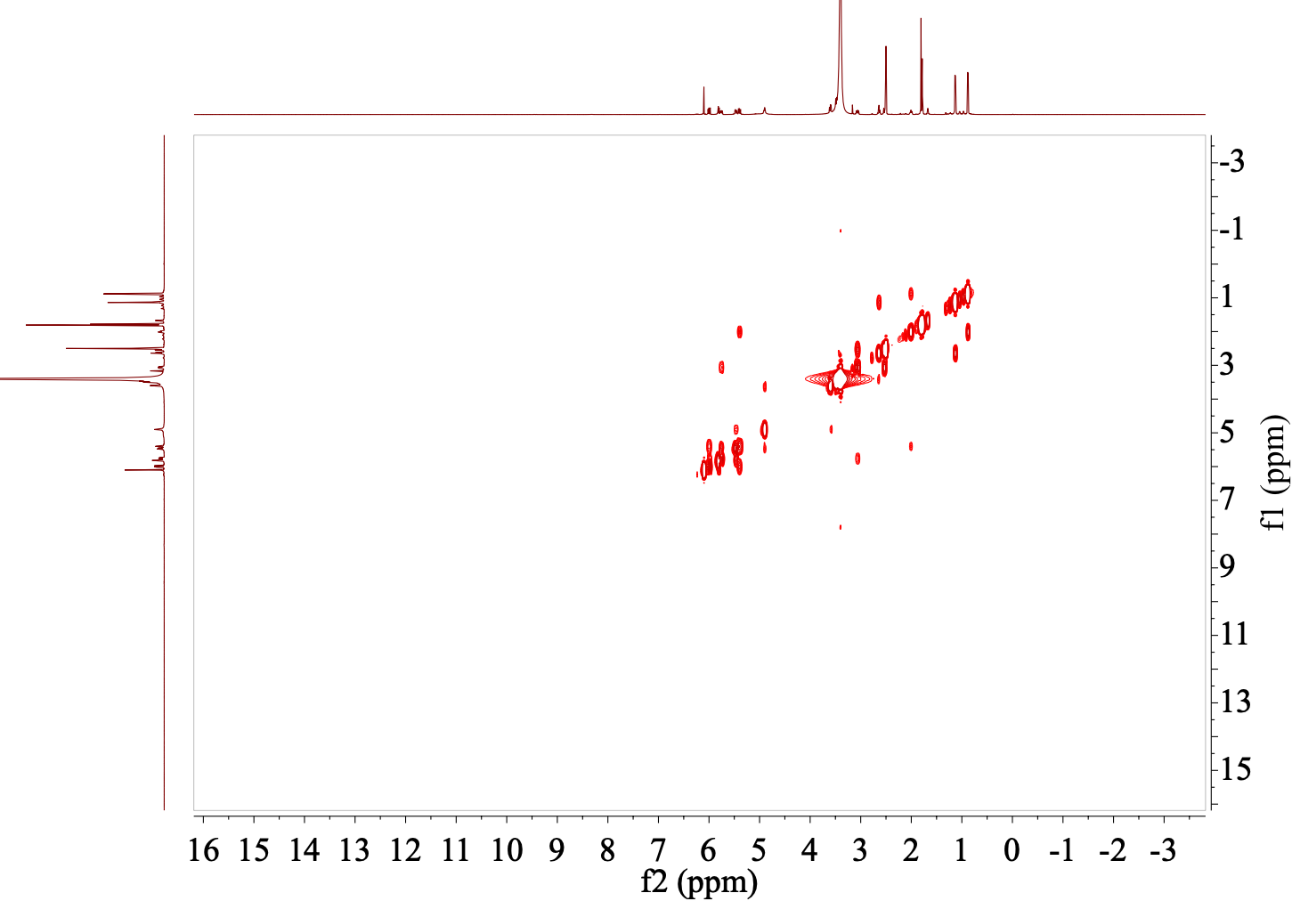


**e.** HSQC of **6**


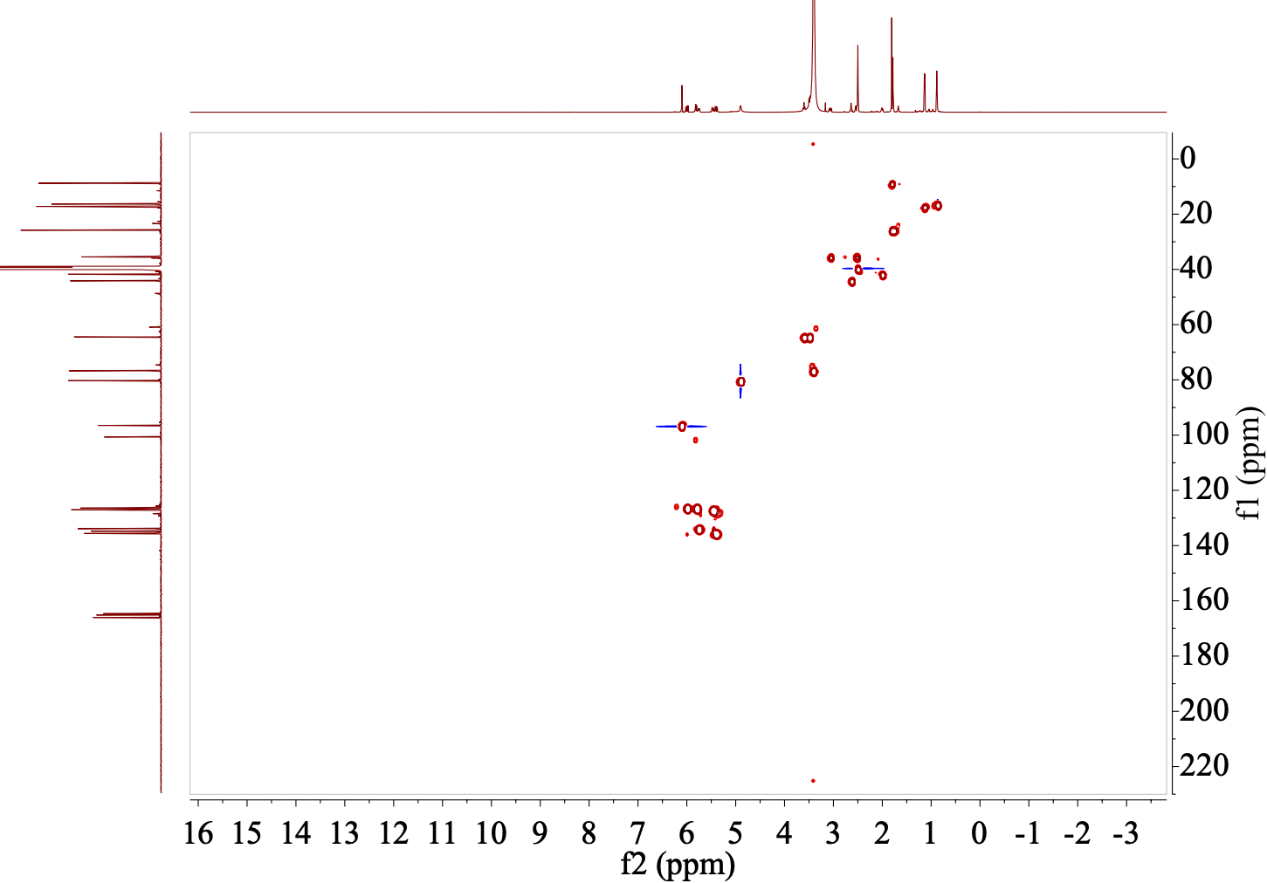


**f.** HMBC of **6**


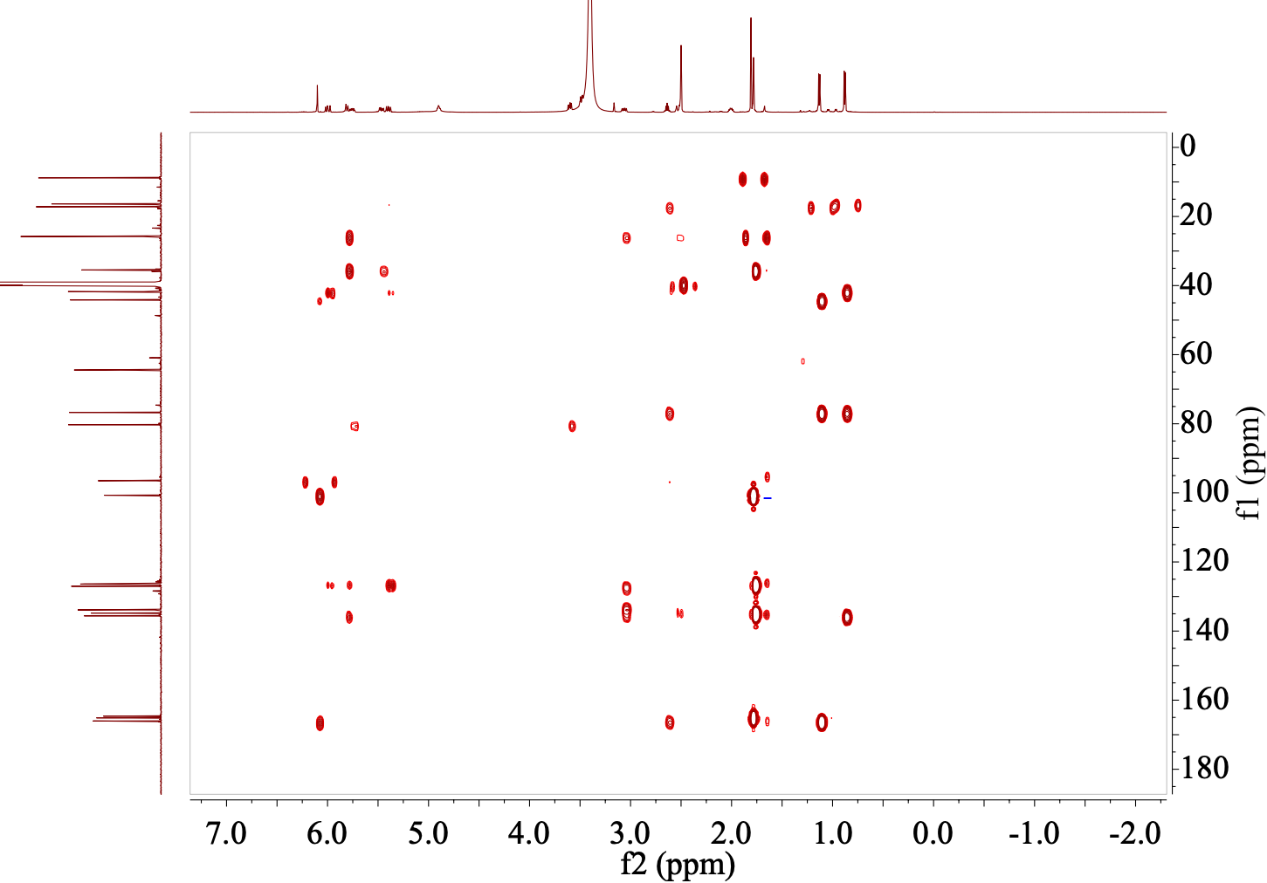


**g.** NOESY of **6**


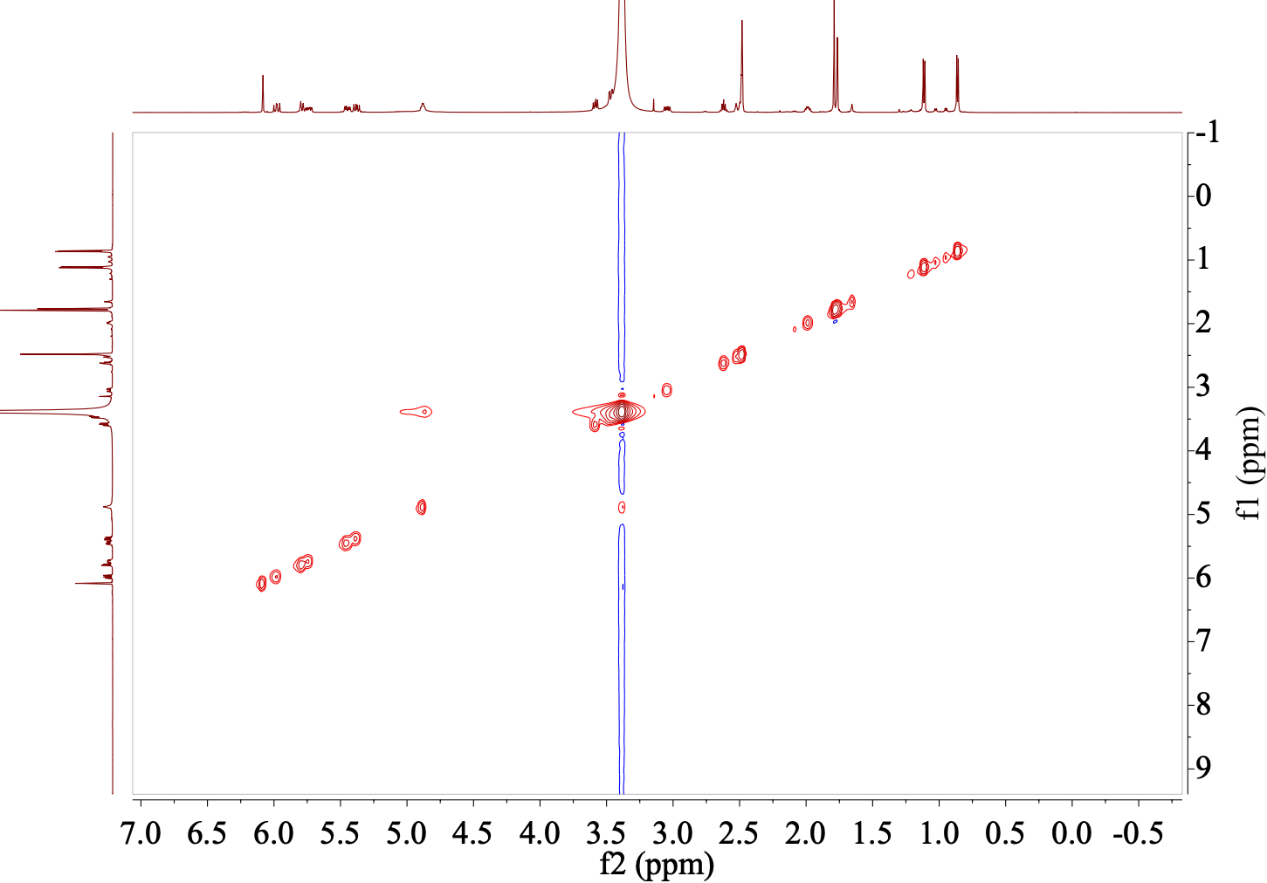


**Fig. S16.** NMR spectra of **7**. (related to Table S9)

**a.** ^1^H-NMR of **7**


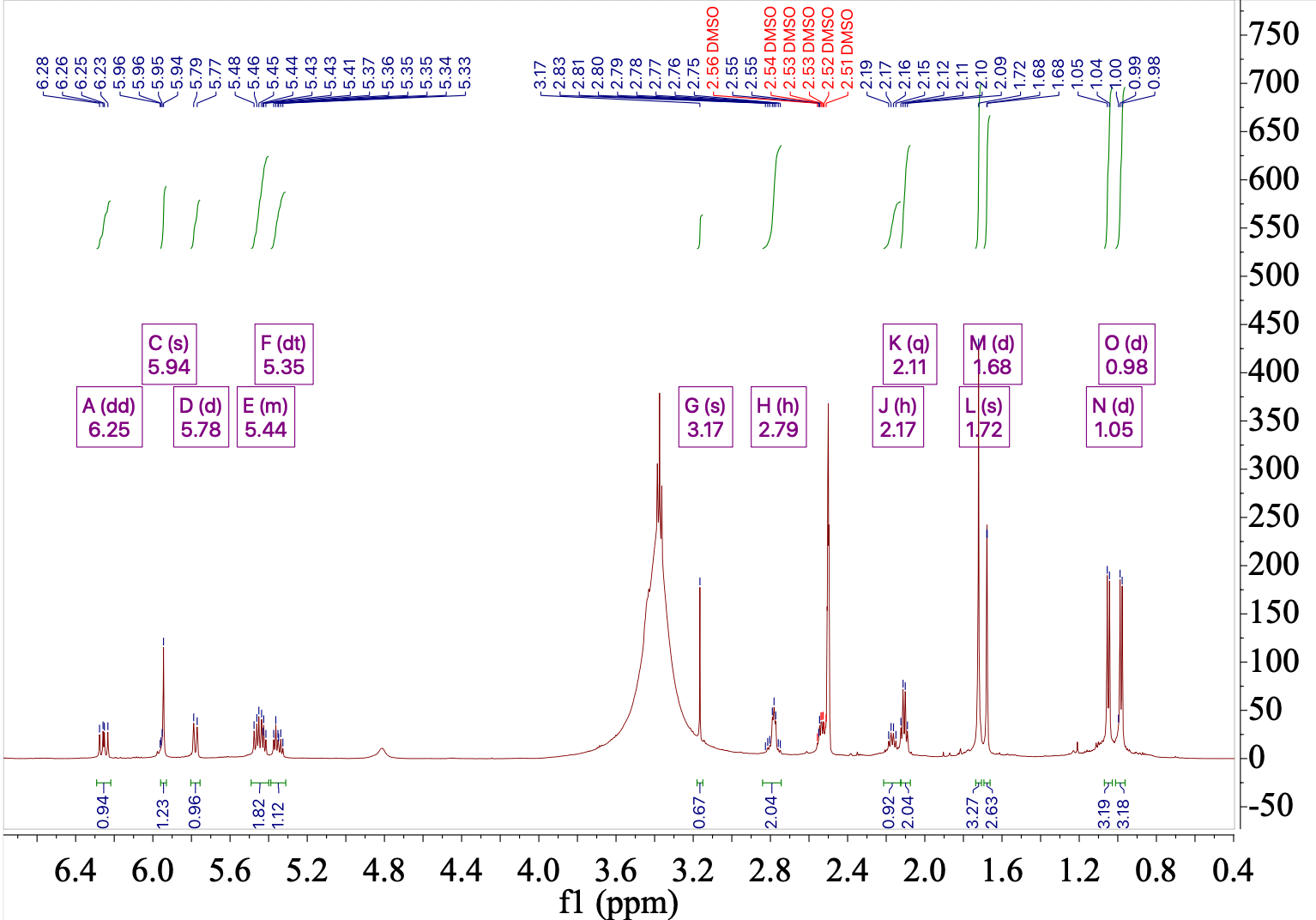


**b.** ^13^C-NMR of **7**


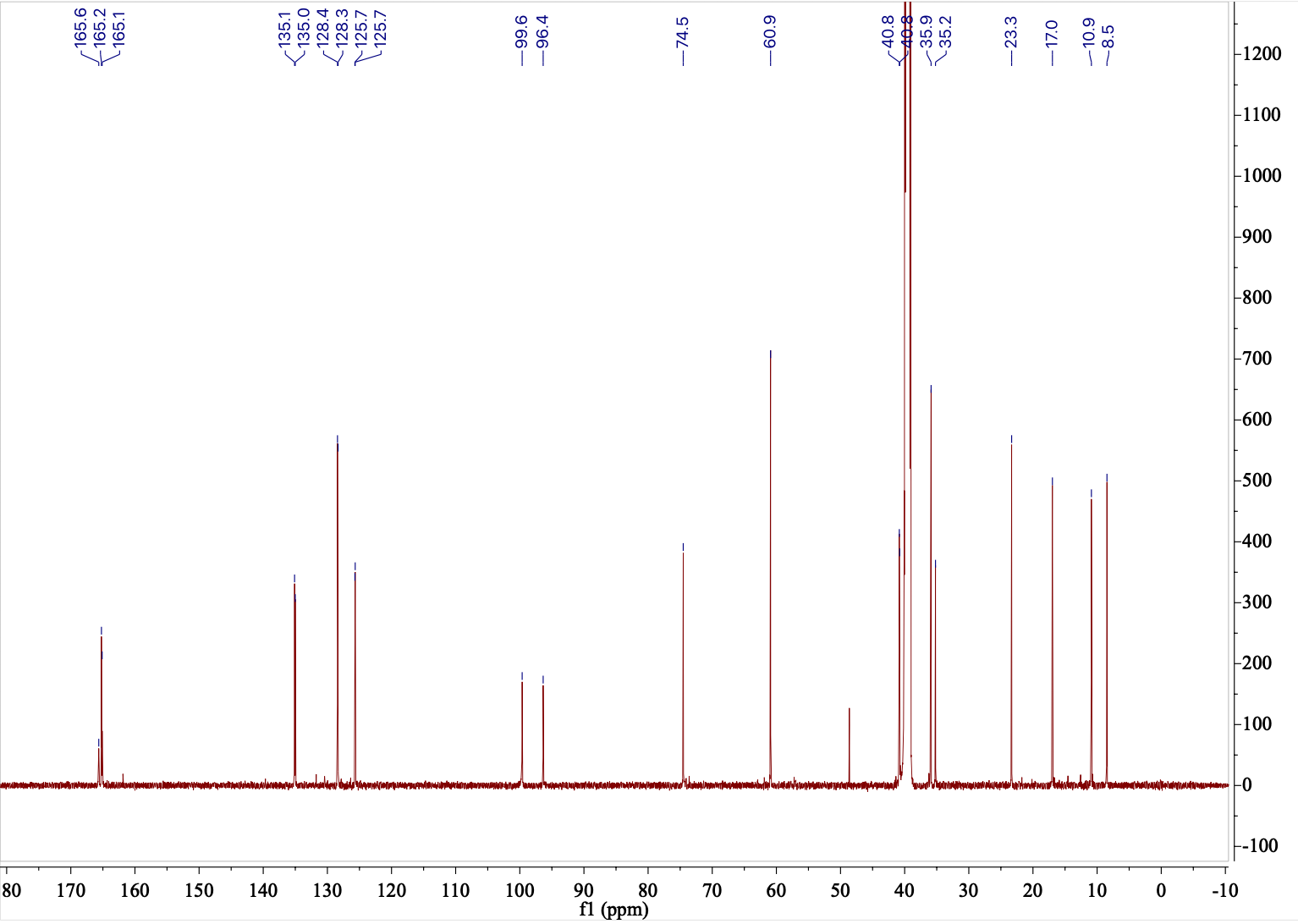


**c.** DEPT-NMR of **7**


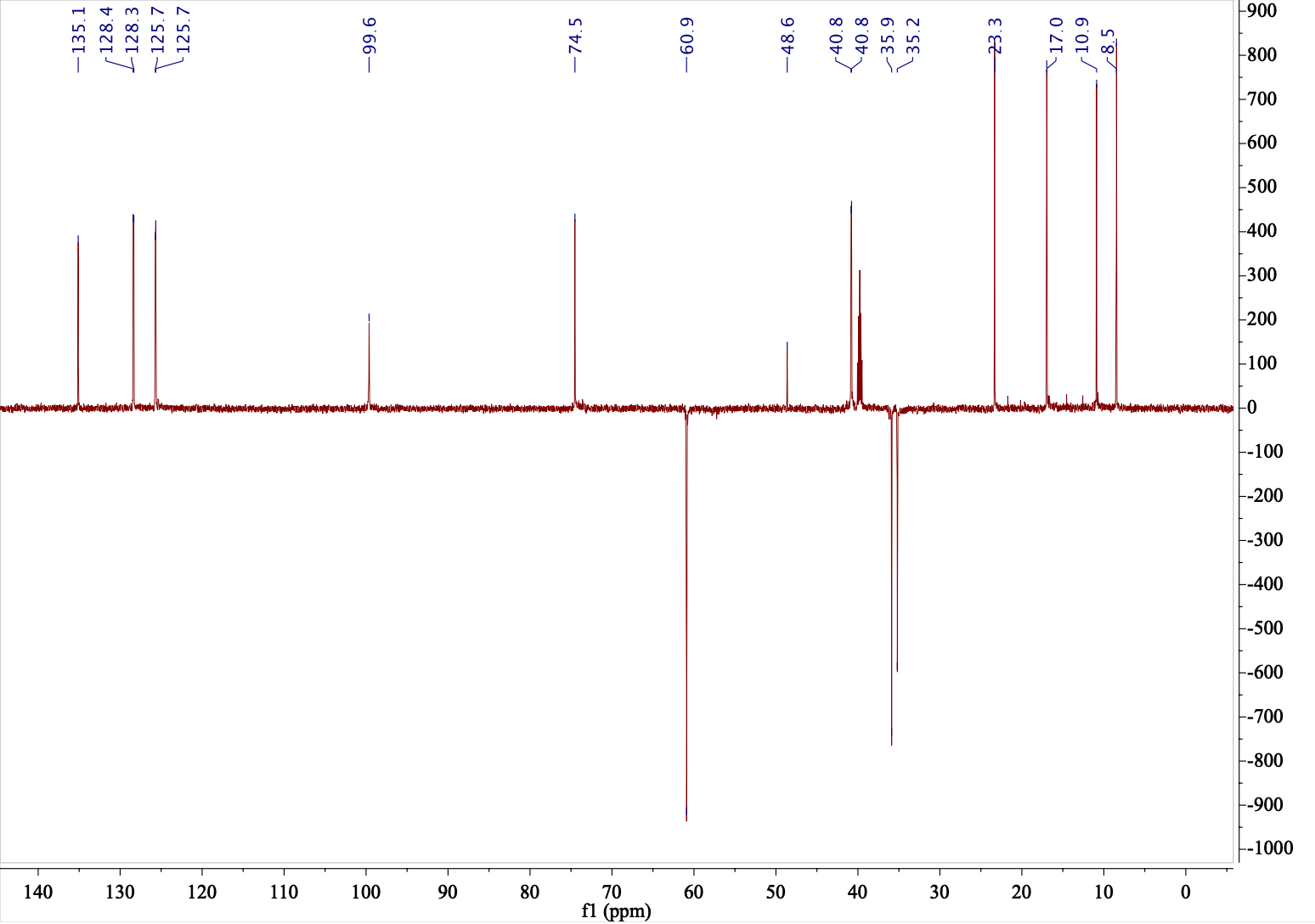


**d.** COSY of **7**


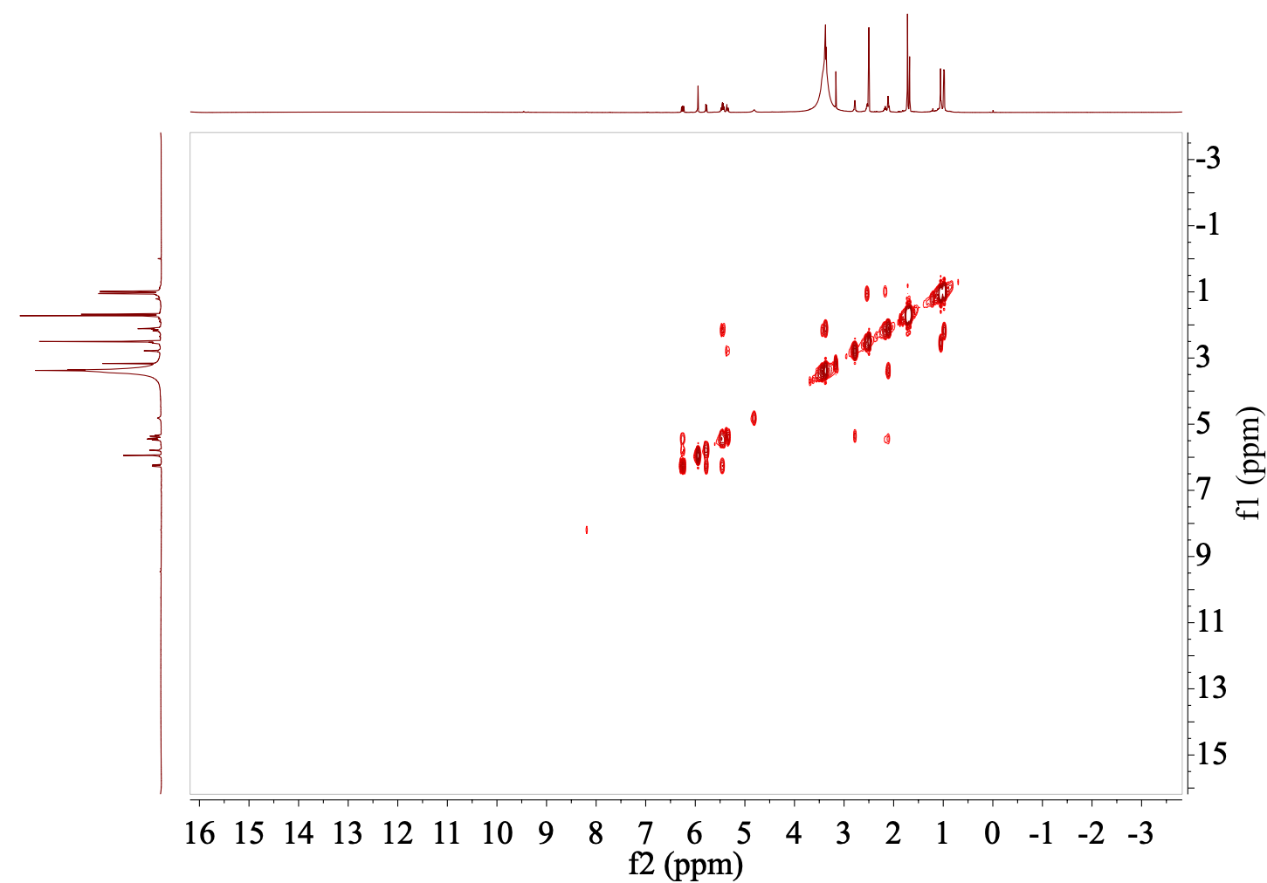


**e.** HSQC of **7**


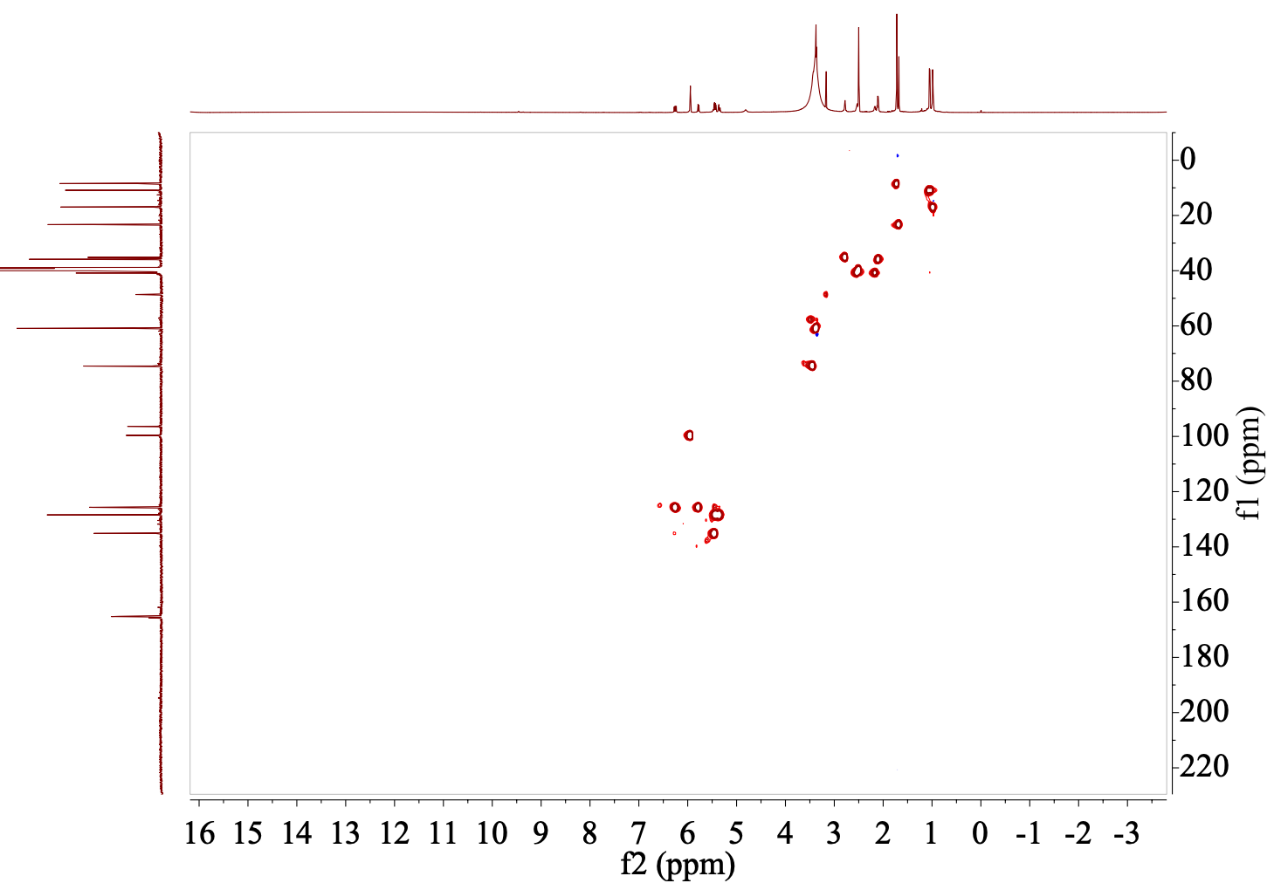


**f.** HMBC of **7**


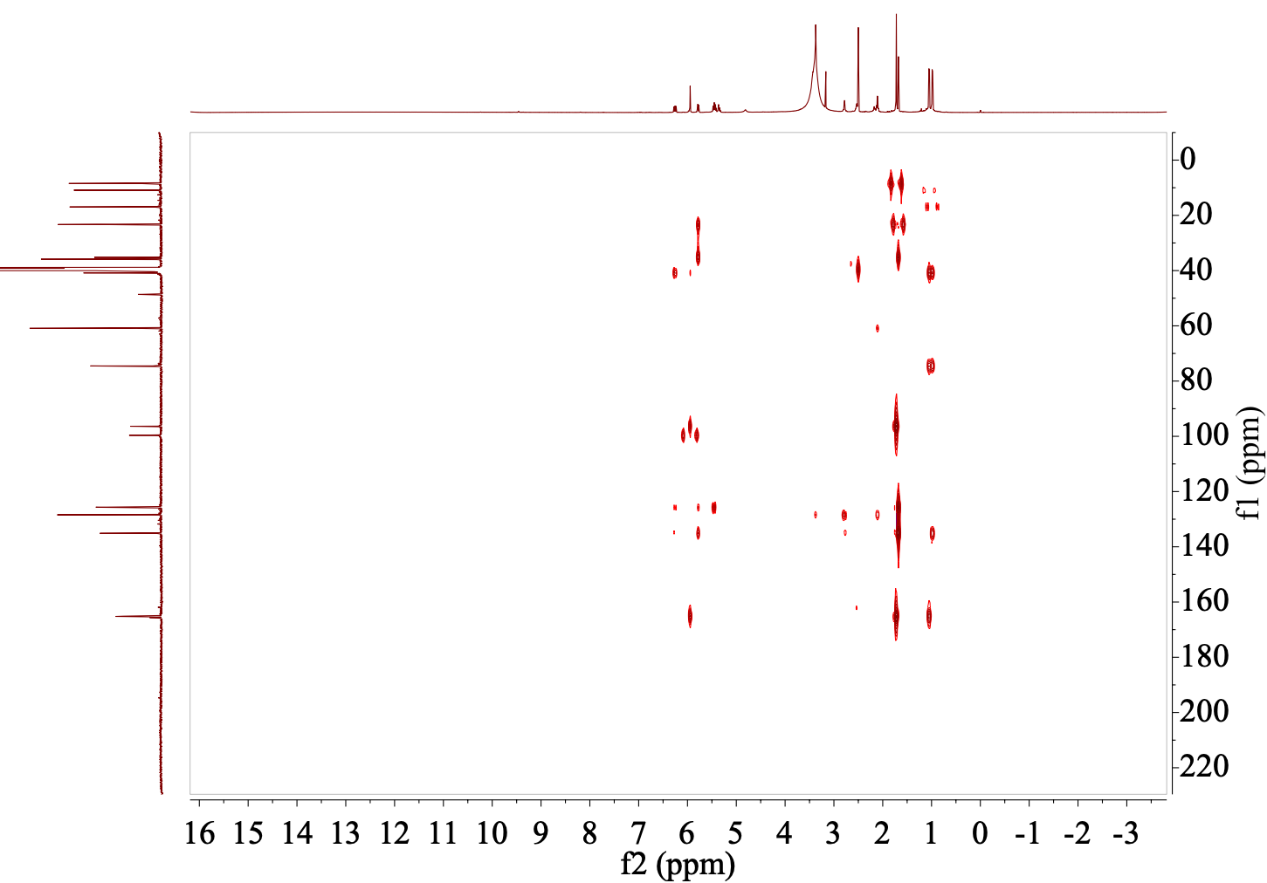


**g.** NOESY of **7**


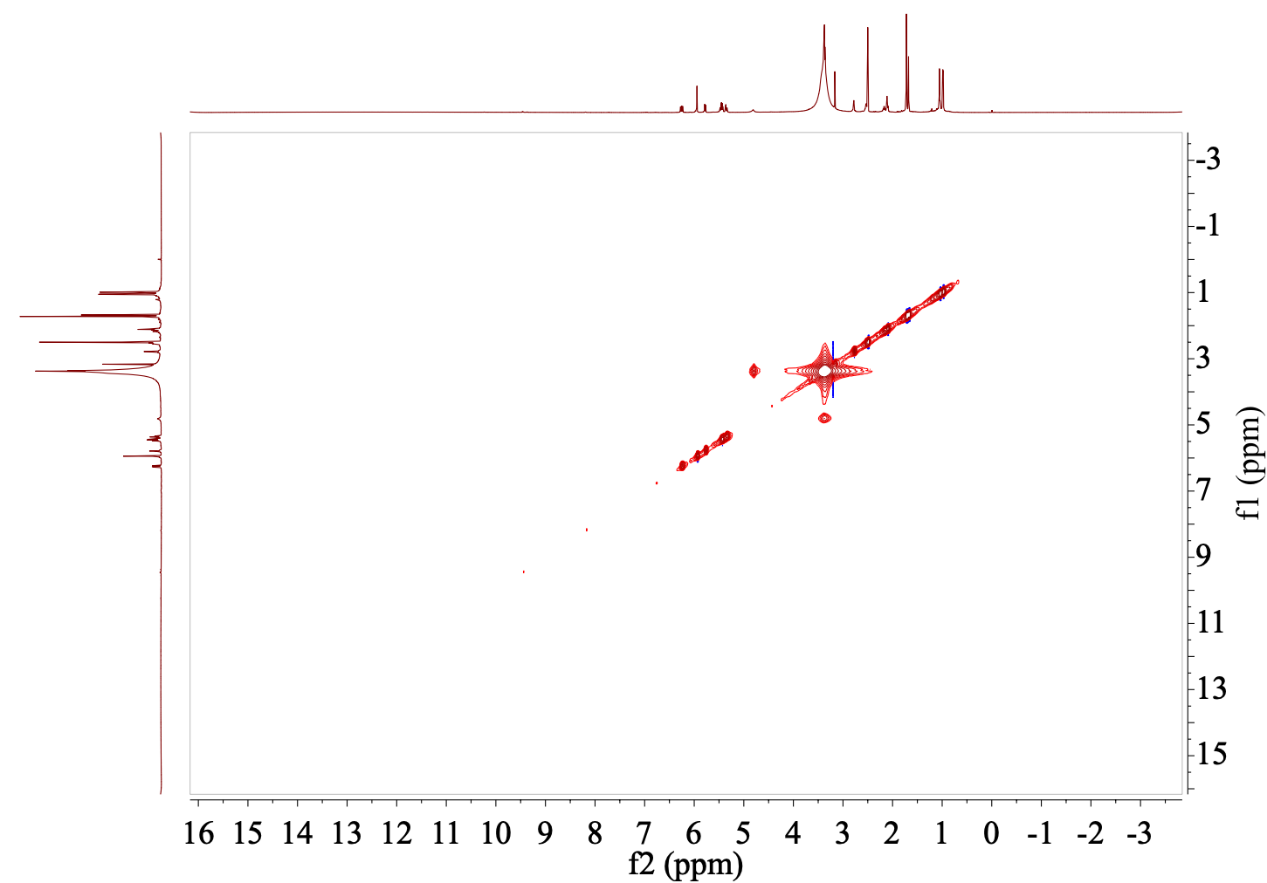


**Fig. S17.** NMR spectra of **8**. (related to Table S10)

**a.** ^1^H-NMR of **8**


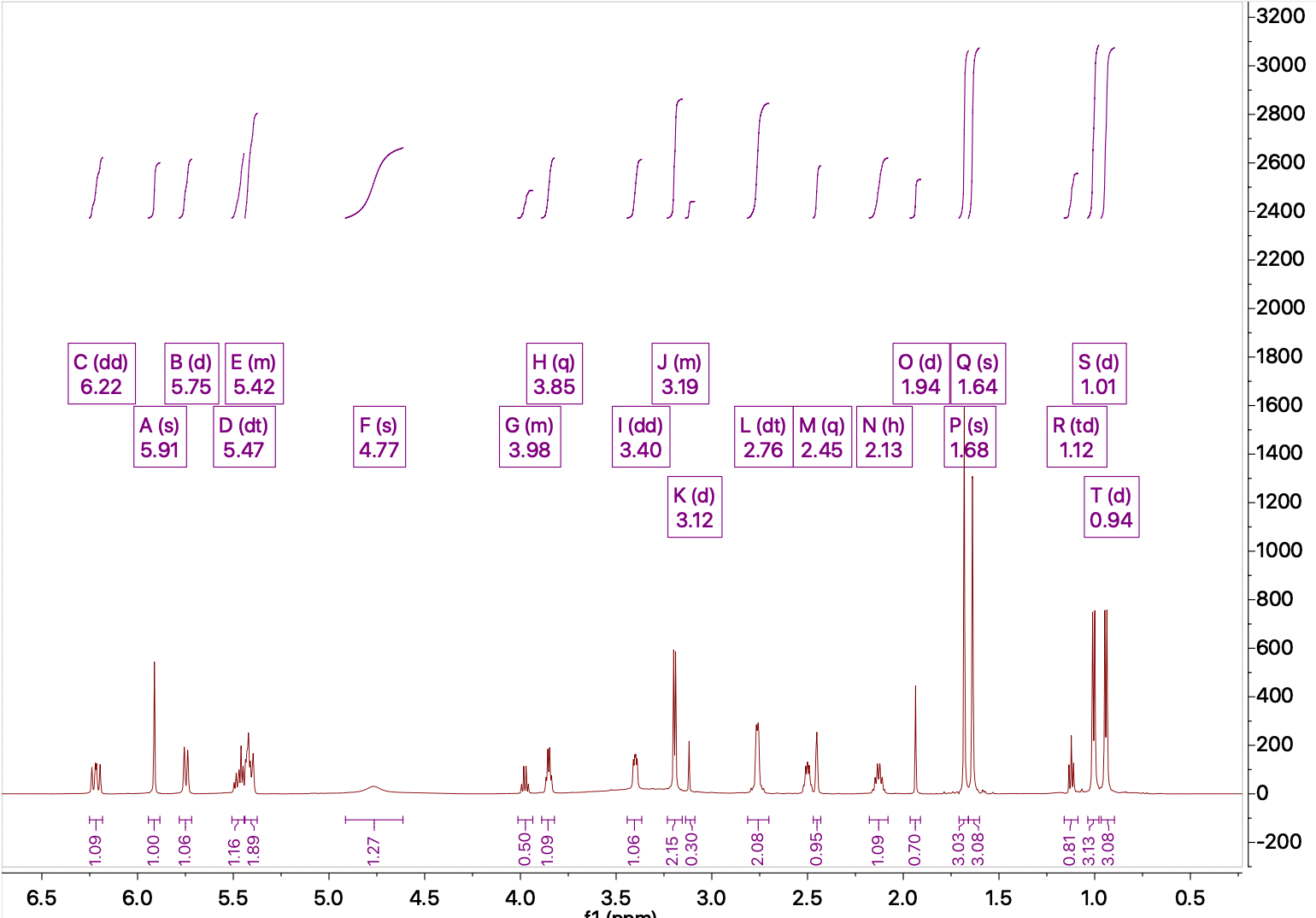


**b.** ^13^C-NMR of **8**


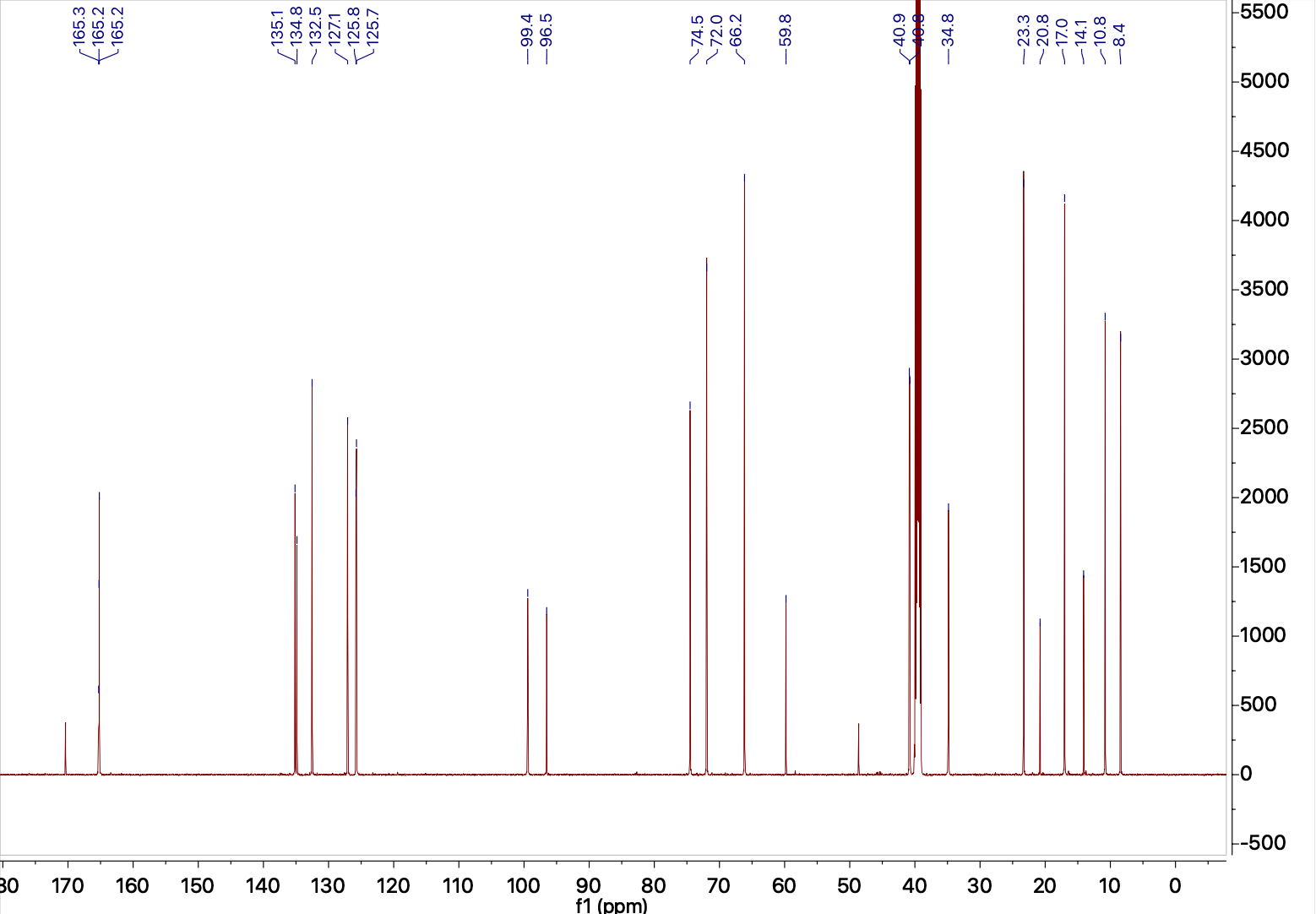


**c.** DEPT-NMR of **8**


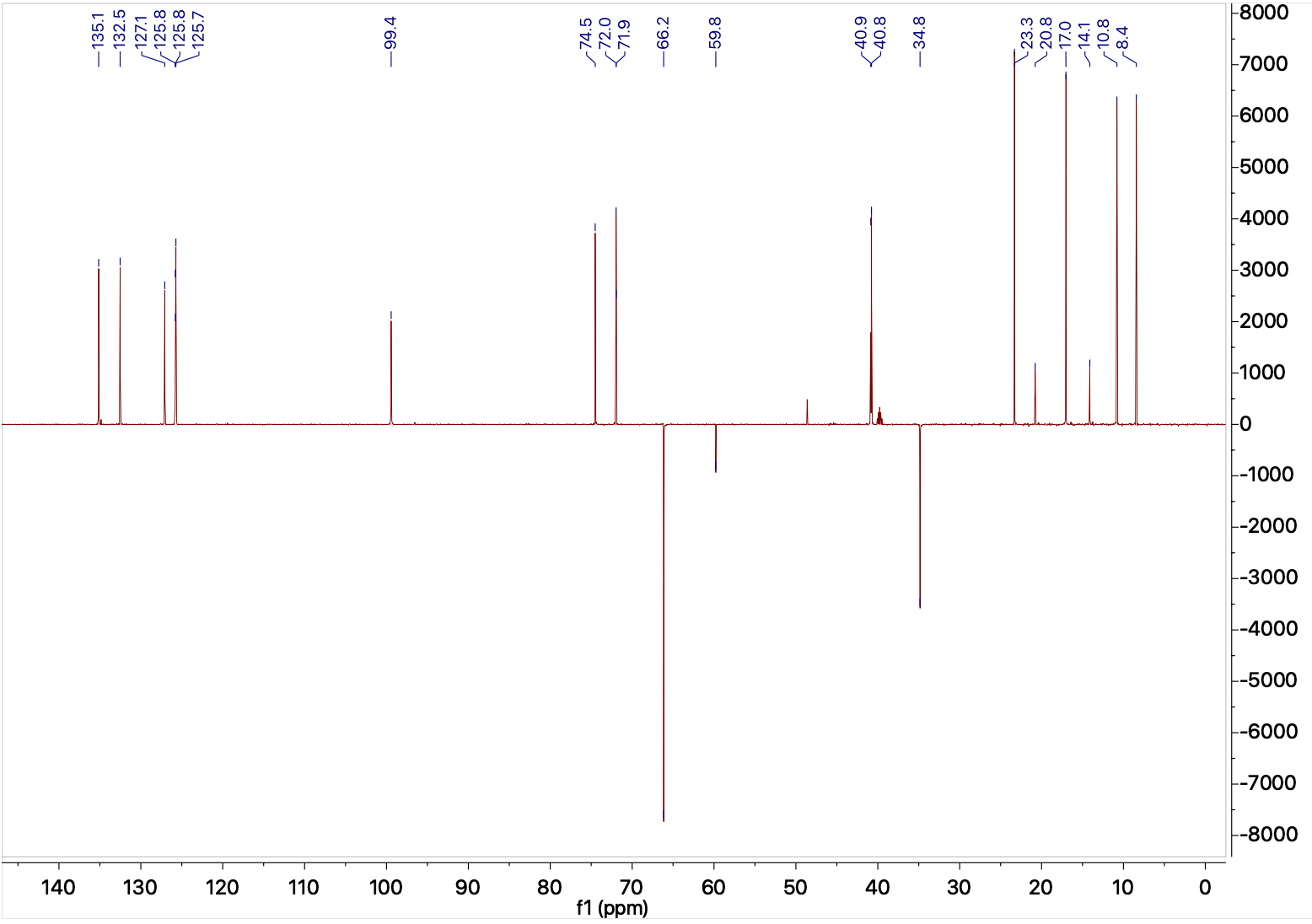


**d.** COSY of **8**


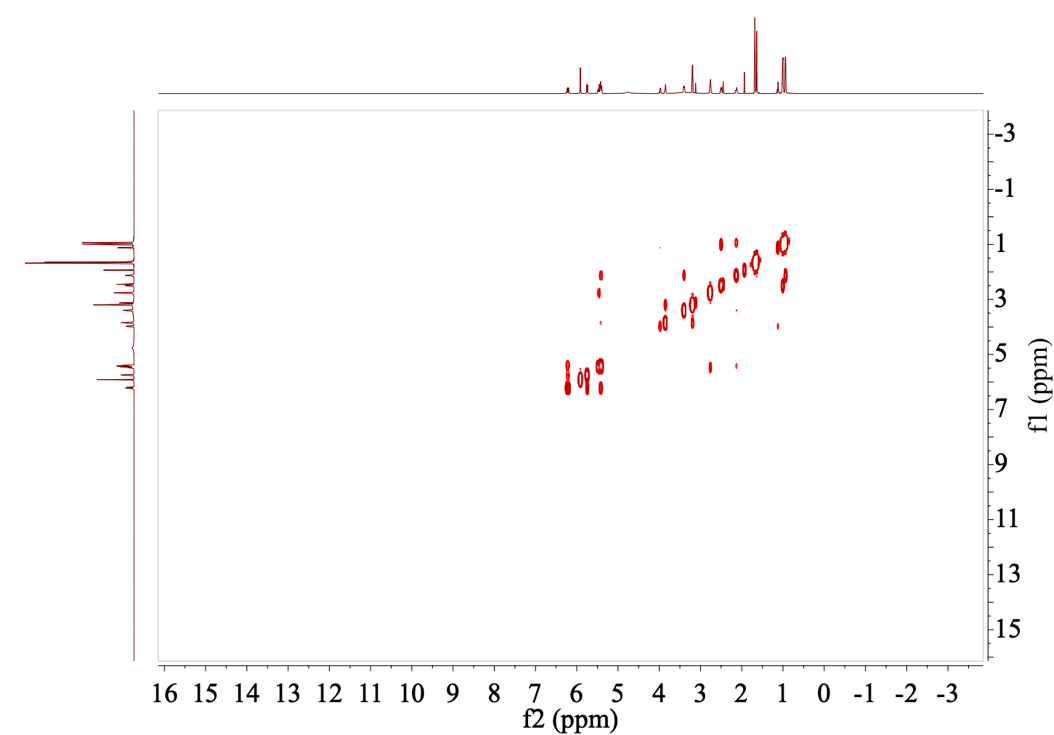


**e.** HSQC of **8**


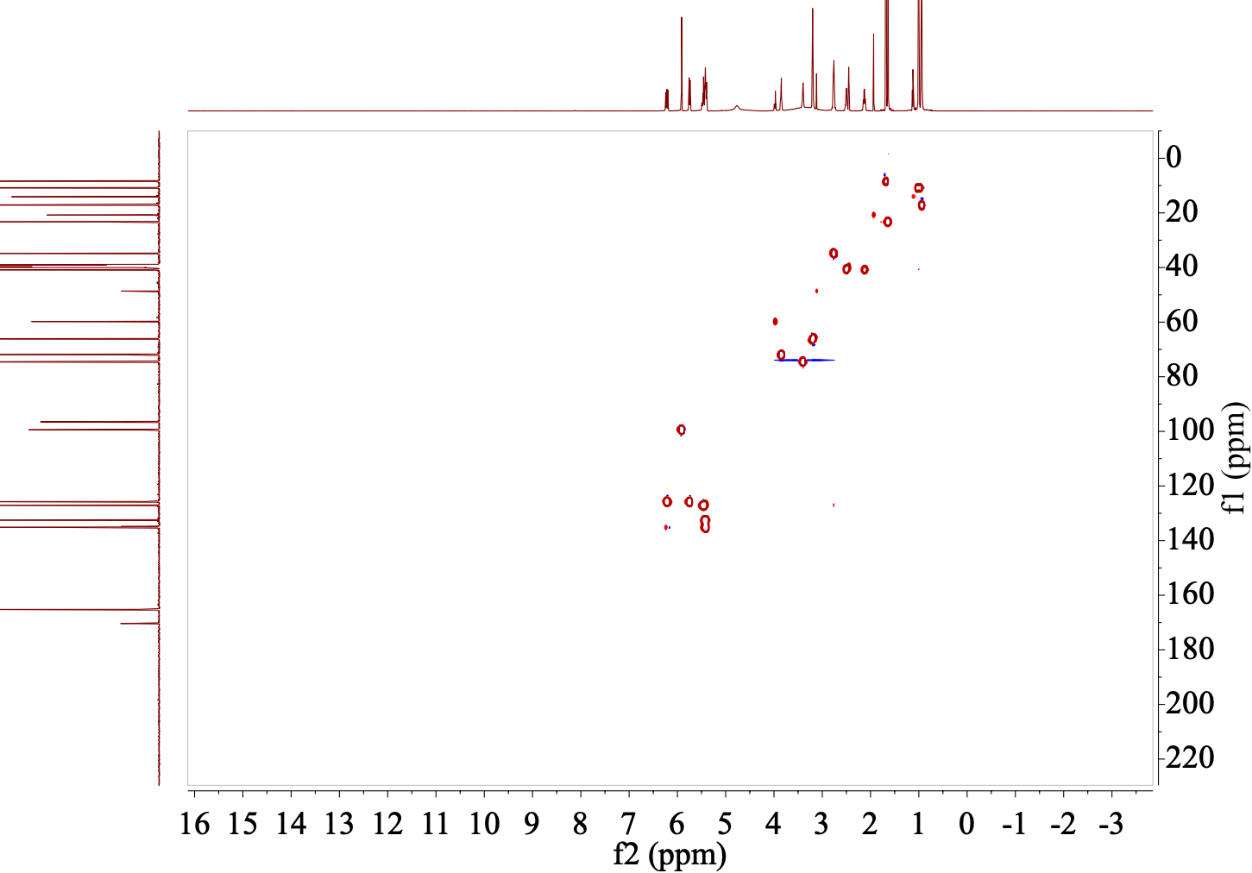


**f.** HMBC of **8**


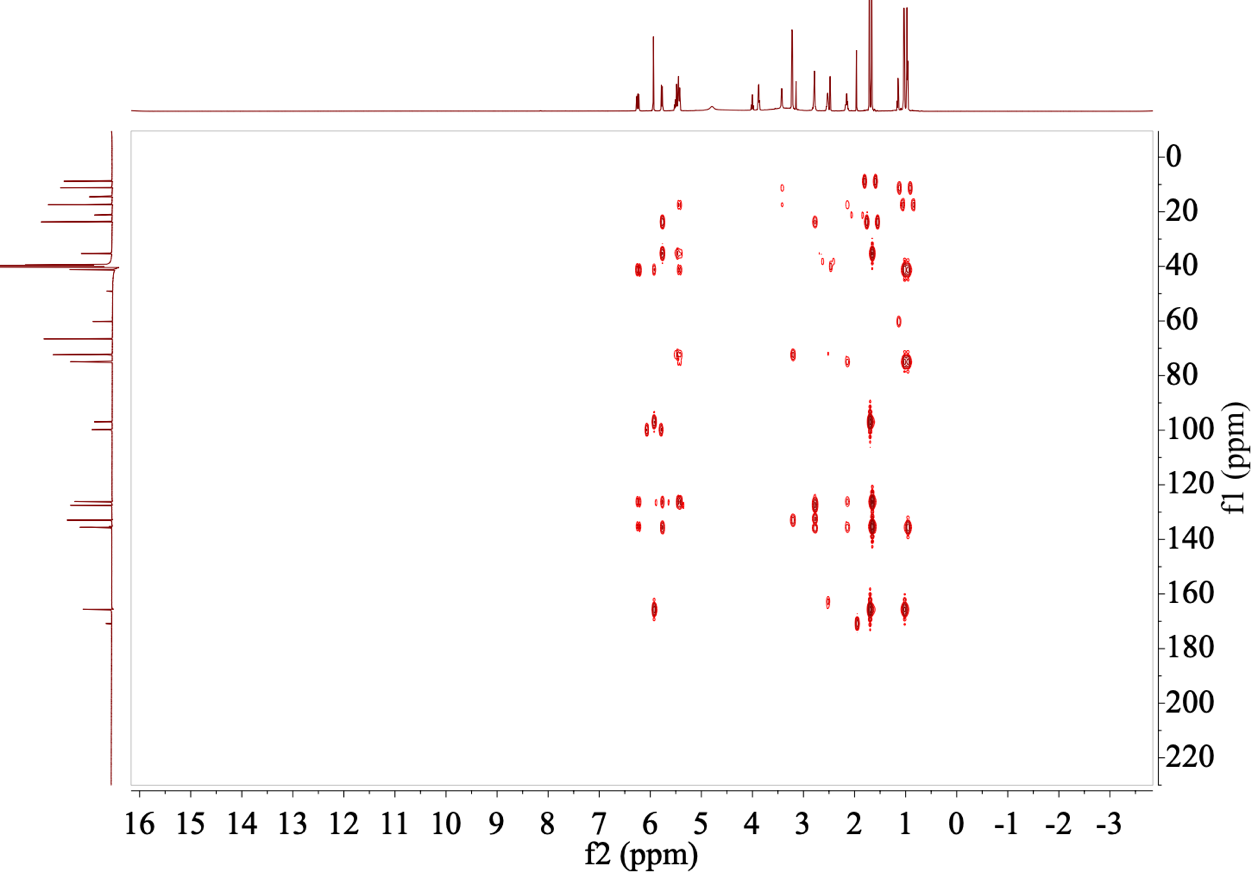


**g.** NOESY of **8**


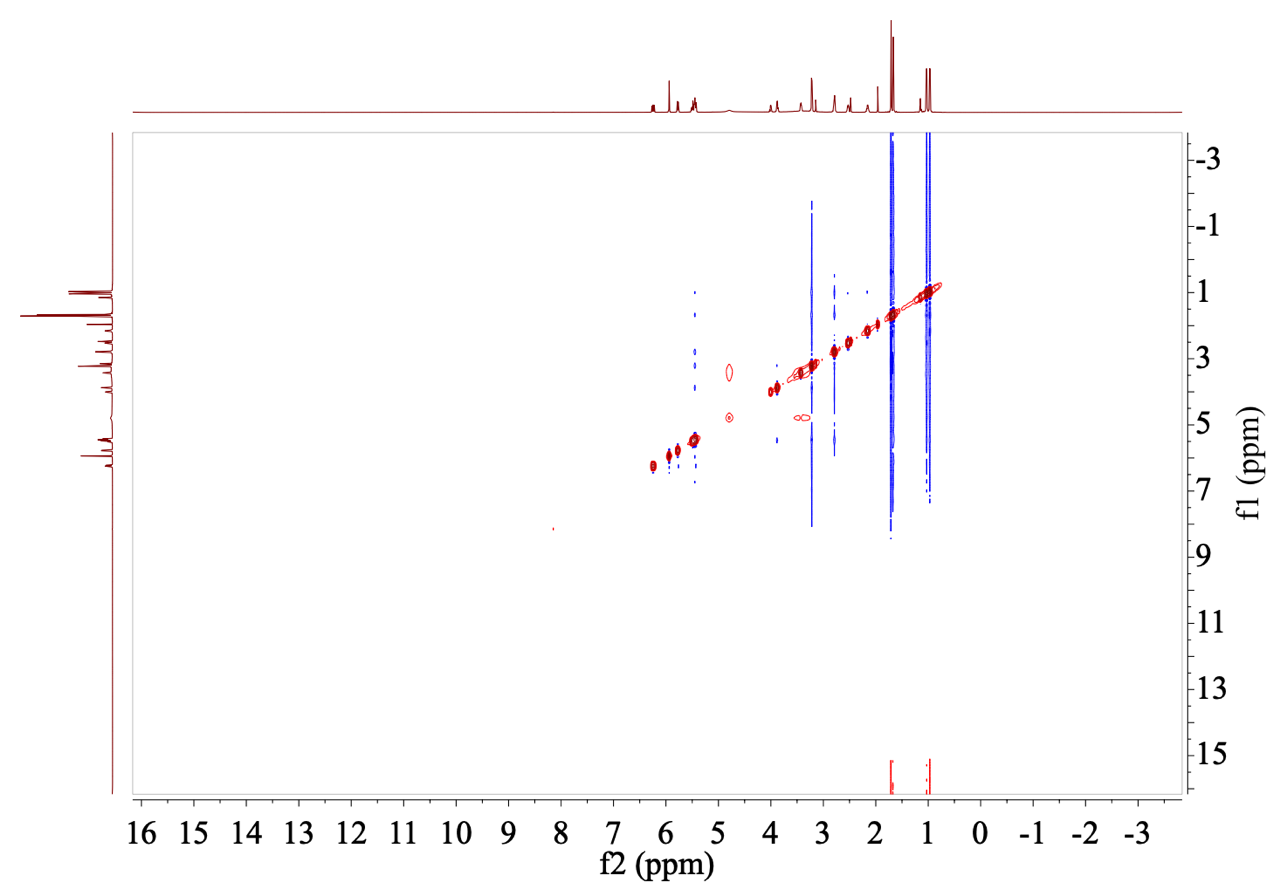

Supplement: Multimedia component 1 [file mmc1.docx]
